# Periplasm-enriched fractions from *Xanthomonas citri* subsp. *citri* type A and *X*. *fuscans* subsp. *aurantifolii* type B present distinct proteomic profiles under *in vitro* pathogenicity induction

**DOI:** 10.1371/journal.pone.0243867

**Published:** 2020-12-18

**Authors:** Flávia S. Zandonadi, Sílvia P. Ferreira, André V. Alexandrino, Carolina M. Carnielli, Juliana Artier, Mariana P. Barcelos, Nicole C. S. Nicolela, Evandro L. Prieto, Leandro S. Goto, José Belasque, Maria Teresa Marques Novo-Mansur

**Affiliations:** 1 Laboratório de Bioquímica e Biologia Molecular Aplicada, Departamento de Genética e Evolução, Universidade Federal de São Carlos, UFSCar, São Carlos, São Paulo, Brazil; 2 Departamento de Fitopatologia e Nematologia, Escola Superior de Agricultura “Luiz de Queiroz”, Universidade de São Paulo, USP, Piracicaba, São Paulo, Brazil; Institute for Sustainable Plant Protection, C.N.R., ITALY

## Abstract

The causative agent of Asiatic citrus canker, the Gram-negative bacterium *Xanthomonas citri* subsp. *citri* (XAC), produces more severe symptoms and attacks a larger number of citric hosts than *Xanthomonas fuscans* subsp. *aurantifolii* XauB and XauC, the causative agents of cancrosis, a milder form of the disease. Here we report a comparative proteomic analysis of periplasmic-enriched fractions of XAC and XauB in XAM-M, a pathogenicity- inducing culture medium, for identification of differential proteins. Proteins were resolved by two-dimensional electrophoresis combined with liquid chromatography-mass spectrometry. Among the 12 proteins identified from the 4 unique spots from XAC in XAM-M (p<0.05) were phosphoglucomutase (PGM), enolase, xylose isomerase (XI), transglycosylase, NAD(P)H-dependent glycerol 3-phosphate dehydrogenase, succinyl-CoA synthetase β subunit, 6-phosphogluconate dehydrogenase, and conserved hypothetical proteins XAC0901 and XAC0223; most of them were not detected as differential for XAC when both bacteria were grown in NB medium, a pathogenicity non-inducing medium. XauB showed a very different profile from XAC in XAM-M, presenting 29 unique spots containing proteins related to a great diversity of metabolic pathways. Preponderant expression of PGM and XI in XAC was validated by Western Blot analysis in the periplasmic-enriched fractions of both bacteria. This work shows remarkable differences between the periplasmic-enriched proteomes of XAC and XauB, bacteria that cause symptoms with distinct degrees of severity during citrus infection. The results suggest that some proteins identified in XAC can have an important role in XAC pathogenicity.

## Introduction

Citrus fruits are one of the most important worldwide crops with production around 47.5 million tons of fresh oranges in the period of 2019/2020 [1]. In Brazil, the largest global producer of citrus fruits, main production is achieved by São Paulo State and represents billions of US dollars in exports, followed by the USA. São Paulo State and Florida State, in the USA, are the main producers of sweet orange juice and both face epidemics of citrus canker. Citrus canker is one of the most important citrus diseases with severe economic impact. This disease is caused by *X*. *citri* subsp. *citri* (XAC), while *X*. *fuscans* subsp. *aurantifolii* type B (XauB) and *X*. *fuscans* subsp. *aurantifolii* type C (XauC) are weaker causative agents of a milder disease, known as cancrosis. While XauC is restricted to Brazil, XauB occurs in Argentina, Paraguay, and Uruguay [2].

Genomic studies have described detailed characteristic differences between the compared genomes of XAC, XauB, XauC, and other *Xanthomonas* spp. [3]. XAC shares 74% homology with both XauB and XauC for protein-encoding genes. Considering only XauB or XauC this value increases to 87% and 84% respectively. Differences were found in genes related to biofilm formation, especially for *rpfN*, one of XAC’s two phosphotransferase systems (PTS) genes encoding for a sugar porin that regulates xanthan gum synthesis. This gene is absent in XauB, which could explain XauB’s need for glutamate in culture medium as an alternative carbon source, and also its fastidious growth rate, which is similar to *Xylella fastidiosa* that also lacks the *rpfN* gene [3].

The success of the infection by many phytopathogenic bacteria often depends on the transport of virulence factors (usually proteins) to the apoplast and to the host’s cytosol by specific secretion systems [4]. The periplasmic fraction is of particular interest in *Xanthomonas* spp. due to its involvement in known virulence mechanisms. In a previous study done by our group a differential proteomic analysis of the periplasmic-enriched fraction was performed between XAC grown in XAM-M, a minimal medium able to induce *hrp* genes and used as a pathogenicity-inducing medium, and NB medium, a pathogenicity non-inducing medium [5]. Proteins, possibly related with substantial alterations in the cellular envelope metabolism, were detected that were related to several cellular processes, from defense against reactive oxygen species to exopolysaccharide synthesis [5]. Here, we have compared the proteomes of periplasm-enriched fractions from XAC and XauB after being cultured in XAM-M (*in vitro* infectious condition) and also in NB (non-infectious condition). Our results show that XAC and XauB differ greatly in their periplasm-enriched proteomes profiles, which can contribute to our biochemical understanding of the disease.

## Methods

### Bacteria strains, media and culture conditions

The XAC strain 306 [6] and XauB strain ICPB11122 [3] were compared in the present study by proteomic analysis after *in vitro* growth in XAM-M and Nutrient Broth (NB), pathogenicity-inducing and non-inducing media, respectively, that were already previously used for proteomic analysis of XAC [5]. Strains were routinely maintained at 28°C on nutrient agar (NA) plates or cultured in NB, which is a nutritionally rich medium composed of 5 g/L peptone and 3 g/L beef extract (Difco). XAM-M is a minimal medium based on XAM-1 medium [7] and is able to induce pathogenicity in XAC *in vitro* [5]. XAM-M is composed of 7.57 mM (NH_4_)_2_SO_4_, 33.06 mM KH_2_PO_4_, 60.28 mM K_2_HPO_4_, 1.7 mM sodium citrate (C_6_H_5_Na_3_O_7_.2H_2_O), 1 mM MgSO_4_, 0.03% (w/v) casamino acids, 10 mM fructose, 10 mM sucrose, and 1 mg/mL BSA (Sigma), at pH 5.4.

Growth curves were performed by inoculating cells into 400 mL of XAM-M, prepared in triplicate, and incubated at 28°C, on a rotary shaker at 200 rpm. The inoculated cells were obtained from pre-culture volumes of OD_595_ ~1 of XAC or XauB, respectively. The OD_595_ was monitored at every hour along 72 h. For proteomic analysis cells were collected at an OD_595_ of about 1.0 in the same culture conditions. Each experiment was conducted in 3 independent biological replicates (n = 3), submitted to the same analysis that will be described below.

### Preparation of periplasm-enriched fractions

Both XAC and XauB cells were harvested from 400 mL of XAM-M (and also NB) culture triplicate. The periplasmic-enriched fractions were prepared according to a previously reported method [5, 8, 9]. Briefly, the bacterial pellet from each culture was washed twice in 10 mM Tris-HCl, pH 8, 20% sucrose, 1mM EDTA and 1 mM PMSF, and centrifuged (10,000 *g* for 20 min at 4°C). Cells were re-suspended in this same solution with the addition of 3 mg/mL lysozyme and incubated for one hour on ice. After another centrifugation step (11,000 *g* for 30 min at 4°C), the supernatant was collected and TCA was added to up to 10%. Proteins were recovered by precipitation on ice for 30 min, followed by centrifugation at 16,000 *g* for 10 min at 4°C, and washed four times with 70% cold ethanol. Protein pellets were air-dried and solubilized in 300 μL of 7 M urea, 2 M thiourea, 4% CHAPS, 40 mM DTT, 1 mM EDTA, 1 mM PMSF, 10 mM Tris-HCl pH 8.0. Protein concentration was determined [10] and 260 μg protein samples were purified (2-D Clean-Up kit, GE Healthcare) and stored at -20°C.

### 2-DE

Proteomes from XAC and XauB periplasmic-enriched fractions were resolved by IEF using 13 cm linear 3–10 pH IPG strips (GE Healthcare) in an IPGphor system (GE Healthcare), according to manufacturer’s instructions. Protein samples of 260 μg were diluted up to 250 μL of rehydration buffer (GE Healthcare) and incubated for 20 h. IEF was conducted at 50 μA per strip at 20°C using the steps: 100 V for 1 h; 500 V for 1 h; followed by a gradient increase to 1000 V for 50 min and 8000 V for 1 h 25 min; 8000 V for 20 min; 100 V for 10 h, with a total of 16,600 Vh. After IEF, the IPG strips were equilibrated for 15 min, first with 3 mL equilibration buffer [50 mM Tris-HCl pH 8.8, 6 M urea, 30% (v/v) glycerol, 2% (w/v) SDS, 10 mg/mL DTT and trace amounts of bromophenol blue] [11, 12], and then with 3 mL of the same equilibration buffer containing 25 mg/mL iodoacetamide instead of DTT. The second dimensional electrophoresis was performed with 12.5% SDS-PAGE gels (16 x 15 cm gel size) in a Hoefer SE600 system (GE Healthcare), using Tris-Glycine as the running buffer [19] and BenchMark^™^ Protein Ladder (Invitrogen, Life Technologies) as molecular mass standard. 2-DE gels were stained with Coomassie Brilliant Blue R-250 (CBB R-250) [13].

Images of 300 dpi were acquired with an ImageScanner (GE Healthcare). Spot intensity (percentage of volume), molecular weight, and isoelectric point were estimated for each spot using ImageMaster 2D Platinum 7.0 software (GE Healthcare). Percentage of spot volume was chosen as the criteria for spot quantification, and automatically calculated by the ImageMaster software, considering 100% as the sum of the volume of all spots detected in each gel. Raw images triplicates for XAC and XauB, shown in S1A and S1B Fig for each of the two growth conditions tested (XAM-M and NB, respectively), were analyzed by the software by matching the spot pools of one gel, chosen as a reference, and the spot pools of each one of the two other gels. The match analysis for the two reference gels for XAC and XauB grown in XAM-M or NB media was finally performed in an automatic mode, followed by one-way ANOVA statistical analysis of the difference of percentage volume for corresponding spots. Protein spots presenting a significant differential abundance (ANOVA, p<0.05) were isolated from gels for protein identification by MS analysis.

### Protein digestion and MS/MS analysis

Spots were excised and in-gel digested with trypsin Gold (Promega) [14]. For protein analysis, a volume of 4.5 μL was briefly dried in a concentrator and resuspended in 100 μL of 0.1% formic acid. An aliquot of 4.5 μL of the peptide mixture from each spot was analyzed according to previous reports [15, 16]. The peptide mixture was separated using C18 (100 μm × 100 mm) on a RP-nanoUPLC (nanoAcquity, Waters) coupled with a Q-Tof Ultima mass spectrometer (Waters) with a nanoelectrospray source at a flow rate of 600 nL/min, voltage was set to 3.5 kV, cone voltage of 30 V and the source temperature was 100°C. The gradient was 2–90% acetonitrile in 0.1% formic acid over 45 min. The instrument methods were set up in data-dependent acquisition (DDA), operated in the “top three” mode, ion Mode and polarity: ES positive (ES+), in which one MS spectrum was acquired; mass range for MS1: 100–2000 (Da), followed by an MS/MS analysis of the top three most-intense peaks detected [17].

### Data analysis

Data were analyzed according to previous works [15]. Spectra were acquired using MassLynx v.4.1 software and raw data files were converted to peak list format (.mgf) by Mascot Distiller v.2.3.2.0 software, 2009 (Matrix Science Ldt.). Homology was searched for XAC or XauB data using Mascot engine v.2.3.01 (Matrix Science Ltd.) against respective genomic databases of XAC strain 306 (Accession Number NC_003919, 5.4 Mb; 43427 sequences; one chromosome and two plasmids) or XauB strain ICPB11122 (Accession Number GenBank ACPX00000000; 4.87 Mb; 3802 sequences; one chromosome and two plasmids) downloaded from NCBI. Parameters for the homology search included carbamidomethylation as a fixed modification, oxidation of methionine as a variable modification, one trypsin missed cleavage and a tolerance of 0.1 Da for both precursor and fragment ions. Identified proteins from XAC were classified into functional categories according to the XAC genome database [6], whereas categories for XauB proteins were assumed to be the same as the XAC homologue protein found by Blast search in the XAC database. Only proteins for which peptides were identified with the highest score value(s) above MASCOT’s threshold value were considered. The Mascot outputs were loaded into Scaffold Q+ (Proteome Software Inc., Portland, OR) [18]. Peptide identifications were accepted if they could be established at a probability higher than 95%, while protein identifications were accepted if they showed a probability higher than 99%. Raw files from mass spectrometry analyses were deposited in the Peptide Atlas data repository and can be accessed through the PASS01335 number.

We have additionally performed BlastN and BlastP database searches to investigate sequence similarity and genomic context of xylose isomerase (XI) genes in the XAC and XauB genome sequences at NCBI.

### Western blot

Validation of PGM and XI differential expression between XAC and XauB was performed by Western blot as described previously [5], with minor modifications, using cells grown in NB (pathogenicity non-inducing medium), XAM-M medium or XAM-X (XAM-M containing 10 mM of xylose). Briefly, equal protein amounts per lane were used from the periplasm-enriched fractions and separated by SDS-PAGE 12 or 15% [19] in duplicate. One gel was stained with CBB R-250 (and destained with 30% methanol, 10% acetic acid) or Silver Blue and the other one was electroblotted onto a nitrocellulose membrane (Hybond-C Extra, GE Healthcare). The blot was stained with 0.5% Ponceau S (Sigma) in 0.1% acetic acid to verify equal loading in each lane, as described by Pedras & Minic (2012) [20]. After destaining in water, the membrane was incubated for 5 minutes in TBST (20 mM Tris pH7.4, 0.5 M NaCl, 0.05% v/v Tween-20) and overnight in 9% defatted milk in TBST, washed three times in this buffer and again incubated with an antiserum (1:5000) raised in rabbit by Proteimax (São Paulo-SP, Brazil) against XAC recombinant PGM [21] or XAC recombinant XI (Nicolela & Alexandrino, unpublished results). After washing for three times in TBST, primary antibodies were detected with 1:3000 dilution of anti-rabbit HRP conjugate (ECL Western Blotting kit, GE Healthcare) in a ChemiDOC MP Imaging System (Bio-Rad). Raw images are shown in S1C and S1D Fig.

## Results

### Growth curves for XAM-M

**Fig 1:**
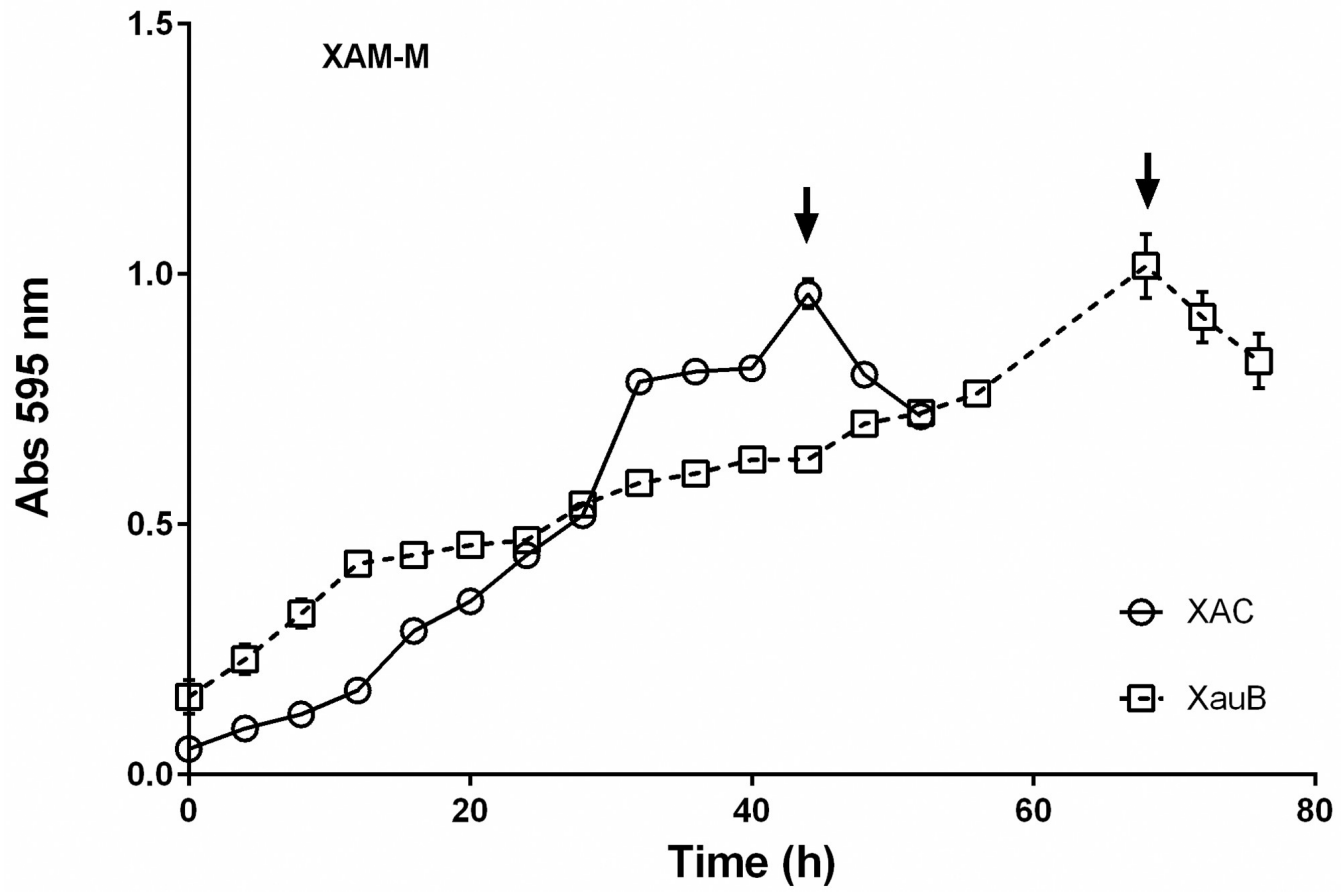
Growth curves of XAC and XauB in XAM-M culture medium by measuring optical density at 595 nm. Error bars indicate standard deviation found within triplicates. XAC and XauB were recovered for periplasmic protein extraction at the points indicated by arrows.

Growth curves were performed to compare the profiles of XAC and XauB when grown in XAM-M (Fig 1). To obtain enough XauB cells for proteomic analysis at the same stage of XAC growth, XAC and XauB cultures were inoculated with cellular proportion of about 1:4, respectively. A maximum OD_595_ of 1.0 was reached after 45 h and 70 h for XAC and XauB, respectively, at which time XAC and XauB cells were harvested for the proteomic analysis (Fig 1).

### Proteomic analysis

**Fig 2:**
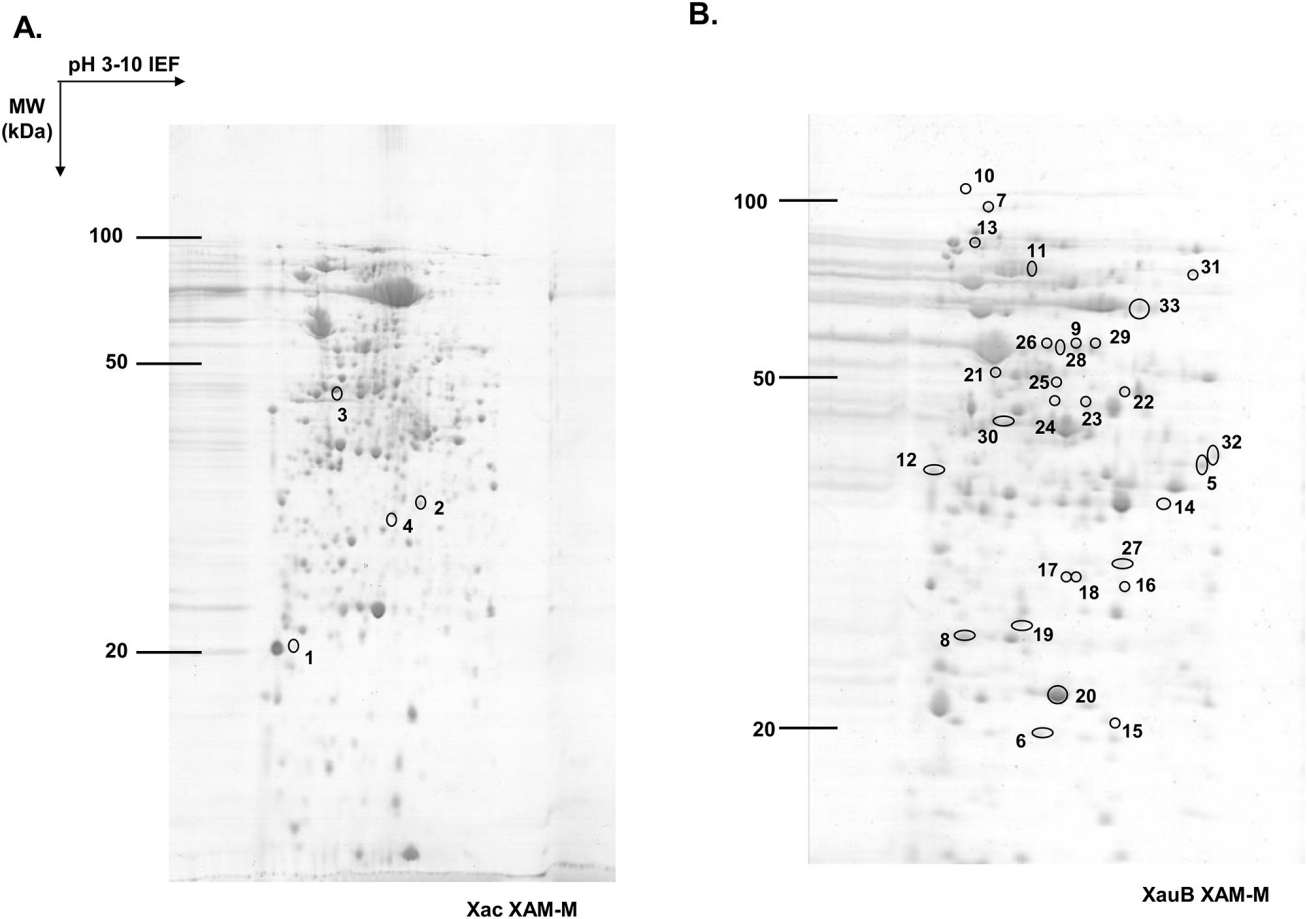
2-DE profiles of periplasm-enriched fractions from XAC and XauB grown in XAM-M pathogenicity-inducing minimal medium. IEF, performed on IPG strips of 13 cm and 3–10 linear pH gradient, was followed by second dimension separation (SDS-PAGE) on 12.5% acrylamide gel. 2-DE gel was stained by Coomassie R-250. Spots presenting a significant differential abundance (ANOVA, p<0.05) were labeled (according to numbers of the Table 1) and excised to be analyzed by MS-MS for protein identification.

Proteome analysis of periplasm-enriched fractions of XAC and XauB was done by comparison of 2-DE patterns after bacteria growth in XAM-M (Fig 2). The number of spots shared between XAC and XauB, based on the position of spots were 756, according to software analysis. Spots that were differential in abundance between the XAC and XauB profiles (statistical ANOVA significance, p<0.05), or were exclusive to XAC or XauB were isolated, digested with trypsin, submitted to MS and identified by homology using respective XAC or XauB genomic data, as presented on Table 1. Among the thirty-three spots with differential abundance between XAC and XauB after growth in XAM-M medium (p<0.05), only four were detected for XAC, from which 12 proteins were identified (Table 1, S1 Data), whereas 29 spots were detected for XauB (Table 1, S2 Data).

**Table 1:** Proteins identified by ESI-Q-TOF in spots that were differential in abundance (p<0.05) between XAC and XauB 2D profiles from cells grown in XAM-M medium.

Spot	Bacterium	% Volume of the unique spots	Spot relative abundance (XAC/XauB)**	ANOVA (p<0,05)	NCBI accession number	Homologue protein^a^ (Exclusive unique peptides count)^b^	Theoretical^c^		Experimental^d^		Mascot Score	Matched Peptide	Sequence Coverage %	Category of XAC protein^e^	Cellular Location^f^
							MW (kDa)	pI	MW (kDa)	pI					
1	XAC	7,72E-02	Unique	0,00138736	XAC0901	Conserved hypothetical protein (4)	20.2	4.8	19.6	4.8	200	8	38	VIII	-
					XAC0223	Conserved hypothetical protein	21.9	4.6			20	1	5	VIII	OM
2*	XAC		1,312	0,0207356	XAC0222	NAD(P)H–dependent glycerol-3-phosphate dehydrogenase (9)	35.9	5.8	33.1	6.2	181	16	41	III	C+
3	XAC	6,11E-01	Unique	0,0298956	XAC3579	Phosphoglucomutase / Phosphomannomutase (10)	49.3	5.2	45.1	5.2	2018	61	65	VII	P
					XAC1719	Enolase (16)	46.0	5.0			161	4	8	I	C
					XACb0007	Lytic murein transglycosylase (2)	46.2	5.9			128	5	14	IV	M+
					XAC0957	Elongation factor Tu (4)	43.3	5.5			88	4	10	III	C
					XAC1776	Xylose isomerase (2)	48.5	5.3			71	3	7	I	C
					XAC1158	Adenylosuccinate synthetase (2)	46.5	5.4			64	2	7	II	C
					XAC3236	Succinyl-CoA synthetase b subunit (3)	41.5	4.9			48	5	14	I	C
					XAC3463	TolC protein (3)	49.6	5.6			37	1	3	VII	OM+
4*	XAC		1,074565525	0,00017655	XAC0680	6-phosphogluconate dehydrogenase(9)	32.9	5.6	28.9	5.8	698	20	43	I	C
5	XauB	3,91E-01	Unique	0,00989754	XAUB_08340	Conserved hypothetical protein (6)	38.3	6.1	39.1	6.8	259	15	20	II(XAC2352)	C
					XAUB_14890	Polyphosphate-selective porin O (2)	43.3	6.3			50	4	6	IV (XAC3472)	OM+
6	XauB	5,76E-02	Unique	0,0486088	XAUB_32290	Adenylate kinase (11)	19.9	5.3	19.3	5.5	620	24	70	II (XAC3437)	C
					XAUB_24740	Poly hydroxyalcanoate granule associated protein (2)	19.8	5.3			50	2	3	I (XAC1643)	C
					XAUB_29250	DNA-binding related protein (1)	20.4	5.7			24	2	12	III (XAC3123)	C
7	XauB	1,58E-01	Unique	0,00277879	XAUB_07390	Carbamoyl-phosphate synthase large subunit (12)	118.1	4.9	109.8	5.0	148	27	15	II (XAC1862)	P/M+
8	XauB	6,75E-01	Unique	0,0150197	XAUB_40430	50S ribosomal protein L3 (7)	22.9	10.2	24.6	4.9	306	15	38	III (XAC0972)	C
					XAUB_08900	NonF-related protein (3)	24.3	4.9			206	10	21	VII (XAC3491)	-
					XAUB_05770	Elongation factor P (3)	20.9	4.8			144	6	15	III (XAC2380)	C
					XAUB_27940	Two component system regulatory protein (1)	25.4	5.2			58	2	4	I (XAC4023)	C
9	XauB	4,07E-01	Unique	5,832,10–9	XAUB_33110	Anthranilate synthase component I (10)	54.2	5.5	55.3	5.8	417	20	28	II (XAC0476)	C
					XAUB_05850	GDP-mannose pyrophosphorylase (6)	51.0	5.6			260	16	32	VII (XAC3580)	C
					XAUB_39520	S-adenosyl-L-homocysteine hydrolase (4)	53.2	5.5			100	8	14	I (XAC0804)	C
10	XauB	4,21E-01	Unique	0,00248762	XAUB_41080	Isocitrate dehydrogenase	35.7	5.4	38.4	4.6	401	15	25	I (XAC1046)	C
					XAUB_40770	Outer membrane protein (4)	39.7	4.6			131	14	30	IV (XAC1012)	OM+
					XAUB_20300	Glyceraldehyde-3-phosphate dehydrogenase (3)	36.2	6.0			40	3	6	I (XAC3352)	C
11	XauB	2,15E-01	Unique	0,00165929	XAUB_12290	TonB-dependent outer membrane receptor precursor (12)	111.0	5.1	96.4	4.9	284	20	18	III (XAC2743)	OM+
					XAUB_17620	TonB-dependent receptor (11)	106.6	5.0			224	21	25	V (XAC4048)	OM
					XAUB_15610	TonB-dependent outer membrane receptor	109.0	5.2			55	4	3	VIII (XAC2312)	OM+
12	XauB	2,47E-01	Unique	4,68 10–4	XAUB_26290	Secreted protein	40.9	6.3	35.3	6.5	460	27	66	VIII (XAC1434)	M+
13	XauB	1,59E-01	Unique	4,65 10–4	XAUB_15870	Conserved hypothetical protein (2)	24.3	6.3	20.6	6.2	197	11	19	VIII (XAC1532)	-
14	XauB	2,00E-01	Unique	0,0176352	XAUB_28440	Conserved hypothetical protein (7)	29.7	5.7	27.9	6.2	172	16	32	VIII (XAC3966)	-
15	XauB	2,20E-01	Unique	0,0176352	XAUB_41570	Carboxyphosphonoenolpyruvate phosphonomutase	32.1	5.4	29.3	5.7	109	4	12	I (XAC1137)	C
					XAUB_08740	UTP-glucose-1-phosphate uridylyltransferase (8)	32.2	5.5			68	7	25	I (XAC2292)	C
16*	XauB		9,31E-01	0,0176352	XAUB_08740	UTP-glucose-1-phosphate uridylyltransferase (8)	32.2	5.5	30.0	5.9	232	13	31	I (XAC2292)	C
					XAUB_09130	Secreted protein (3)	25.2	6.1			47	7	31	VIII (XAC0555)	M+
					XAUB_41570	Carboxyphosphonoenolpyruvate phosphonomutase (3)	32.1	5.4			27	2	4	I (XAC1137)	C
17	XauB	3,92E-01	Unique	0,0341684	XAUB_40240	50S ribosomal protein L25 (6)	23.3	5.2	25.7	5.3	500	12	41	III (XAC0951)	C
					XAUB_17400	Conserved hypothetical protein (11)	29.1	5.2			25	2	5	VIII (XAC0623)	OM+
18	XauB	2,67E-01	Unique	0,0126284	XAUB_17400	Conserved hypothetical protein (11)	29.1	5.2	30.1	4.7	505	23	42	VIII (XAC0623)	OM+
19	XauB	1,43E-01	Unique	0,0212073	XAUB_08010	ATP synthase subunit B (11)	51.0	5.2	50.4	5.1	388	18	38	I (XAC3649)	C
					XAUB_17520	ATP-dependent protease ATP-binding subunit (2)	50.8	5.4			61	10	18	III (XAC0638)	C
					XAUB_15880	dihydrolipoamide dehydrogenase (14)	50.7	5.8			51	1	3	I (XAC1533)	C
					XAUB_16560	cystathionine b-synthase (4)	49.9	5.2			37	2	3	II (XAC3603)	C
					XAUB_12060	histidinol dehydrogenase (2)	45.1	5.0			32	3	6	II (XAC1829)	C
					XAUB_21320	aldehyde dehydrogenase (1)	54.4	5.4			30	1	1	I (XAC1808)	C
20	XauB	1,47E-01	Unique	0,00927151	XAUB_15880	Dihydrolipoamide dehydrogenase (14)	50.7	5.8	50.4	5.1	543	34	47	I (XAC1533)	C
21	XauB	1,67E-01	Unique	0,0143567	XAUB_15890	Dihydrolipoamide acetyltransferase (13)	42.4	5.9	42.3	6.5	57	1	3	I (XAC1534)	C
22	XauB	3,59E-01	Unique	0,0250698	XAUB_15890	Dihydrolipoamide acetyltransferase (13)	42.4	5.9	46.0	5.9	406	27	44	I (XAC1534)	C
23	XauB	3,25E-01	Unique	0,0152133	XAUB_38740	Betaine aldehyde dehydrogenase (8)	52.7	5.4	54.0	5.8	191	19	28	V (XAC0719)	C
					XAUB_30070	Virulence protein (4)	49.0	5.6			149	10	14	VIII XAC1368)	M+
24	XauB	8,55E-02	Unique	0,00430812	XAUB_20360	Pyruvate kinase (12)	54.8	5.6	59.9	5.4	250	13	16	I (XAC3345)	C
					XAUB_14750	Chaperonin GroEL (6)	56.9	5.0			136	12	15	III (XAC0542)	C
					XAUB_05850	GDP-mannose pyrophosphorylase (6)	51.0	5.6			46	2	**3**	VII (XAC3580)	C
					XAUB_10980	Outer membrane receptor for transport of vitamin B (3)	67.7	5.4			24	2	1	IV (XAC3194)	OM
25*	XauB		0,762	1,77 10–4	XAUB_41470	Acyl-carrier-protein S-malonyltransferase (2)	33.0	5.7	33.1	6.2	43	4	14	II (XAC1126)	C
26	XauB	1,18E-01	Unique	0,00747364	XAUB_20360	Pyruvate kinase (12)	54.8	5.6	61.2	5.3	618	31	26	I (XAC3345)	C
					XAUB_08690	Bifunctional GMP synthase/glutamine amidotransferase protein (3)	57.3	5.4			31	5	7	II (XAC2287)	C
27	XauB	2,01E-01	Unique	0,0182525	XAUB_21500	Glucose-6-phosphate isomerase (10)	54.3	5.8	51.8	6.2	278	22	32	I (XAC1788)	C
28	XauB	1,67E-01	Unique	0,0104587	XAUB_09300	Adenylosuccinate lyase (15)	49.9	5.6	48.2	5.9	739	39	43	II (XAC1539)	C
29	XauB	1,94E-01	Unique	0,0572302	XAUB_08180	NADH dehydrogenase gamma subunit (13)	80.1	6.2	78.9	6.7	403	34	38	VIII (XAC2698)	M
30	XauB	2,96E-01	Unique	2,64 10–4	XAUB_30230	Acetyl-CoA C-acetyltransferase (9)	40.1	6.3	40.1	6.9	337	15	34	II (XAC1348)	-
31	XauB	7,82E-01	Unique	0,0249681	XAUB_14870	phosphate acetyltransferase (4)	84.0	5.6	71.4	6.4	413	24	26	I (XAC3470)	C
32	XauB	2,85E-01	Unique	0,0351553	XAUB_38250	Tail-specific protease (14)	80.1	6.0	75.7	6.1	516	42	34	III (XAC0669)	M
33	XauB	5,87E-01	Unique	0,0209188	XAUB_03410	Formate dehydrogenase acessory protein (3)	29.8	6.0	75.6	6,1	28	2	2	I (XAC2487)	C

XAC and XauB were grown in XAM-M medium, and periplasm-enriched fractions, extracted from both bacteria, were resolved on 2-DE (p< 0.05), and proteins from differential spots were identified by ESI-Q-TOF. The cultivation times were 45 h for XAC and 70 h for XauB.

^a^ Proteins identified using Mascot with XAC or XauB databases (NCBI); not all XauB proteins identified were included, only those ones that presented the highest Mascot scores and/or MW and p*I* more compatible with experimental values; spots number with asterisks were isolated for both bacteria in the same condition.

^b^ Exclusive peptide counts determined for some spots using software Scaffold^™^ (Proteome Software Inc., Portland, OR) for 100% protein identification probability;

^c^ Theoretical molecular weight (MW) and isoelectric point (p*I*) of the matched protein obtained from NCBI database;

^d^ Experimental molecular weight (MW) and isoelectric point (p*I*) were calculated by Image Master Platinum software (GE Healthcare) based on the position of the spot on 2-DE;

^e^ Proteins clustering according to “Xanthomonas axonopodis pv. citri Main Chromosome and Plasmid Gene List” at NCBI [6]: I) Intermediary metabolism, II) Biosynthesis of small molecules, III) Macromolecule metabolism, IV) Cell structure, V) Cellular processes, VI) Mobile genetic elements, VII) Pathogenicity, virulence and adaptation, VIII) Hypothetical, IX) ORFs with undefined category;

^f^ Predicted cellular location of proteins by pSortP 3.0 and SecretomeP 2.0. P, M, C, and OM correspond respectively to periplasm, membrane, cytoplasm, and outer membrane location. Signal (+) indicates the presence of signal peptide according to SignalPeptide 2.0.

**Relative abundance (arbitrary units) for matched spots was calculated as the ratio of the volume percentage average of XAC and XauB. ‘Unique’ spots were detected in only one of the two conditions (XAC or XauB).

Unique peptide lists for the identified proteins (Table 1) are presented in S1 and S2 Tables (for XAC and XauB proteins, respectively). The experimental molecular mass and isoelectric point (p*I*) attributed by the software for the spots based on its position in 2D gel mostly matched to the respective theoretical values for the protein identified by Mascot as having the highest score in the spot (Table 1). Differential proteins detected for XAC or XauB were not from XAC- or XauB-specific genes, since all of them were found to be present in both genomes.

Proteins identified from XAC spots were 6-phosphogluconate dehydrogenase (6PGDH, XAC0680), NAD(P)H-dependent glycerol-3-phosphate dehydrogenase (GpsA, XAC0222), conserved hypothetical proteins (XAC0223 and XAC0901), succinyl-CoA synthetase β-subunit (SucCD, XAC3236), adenylosuccinate synthetase (XAC1158), lytic murein transglycosylase (XACb0007), enolase (XAC1719), elongation factor Tu (XAC0957), phosphoglucomutase/phosphomannomutase (PGM, XAC3579), and TolC (XAC3463). Xylose isomerase (XI) was also detected for XAC (XAC1776, Table 1). All these mentioned proteins detected for XAC in XAM-M are also codified in the XauB genome, by the respective ORFs XAUB_38360, XAUB_35470, XAUB_35460 and XAUB_22380, XAUB_19220, XAUB_050050, XAUB_14690, XAUB_27370, XAUB_40270, XAUB_05860, XAUB_32540. These XauB proteins are more than 98% identical to XAC´s homologue proteins. XI is also codified in XauB genome by ORF XAUB_09030.

With the exception for the lytic murein transglycosylase, enolase and elongation factor Tu, the other proteins were not detected as exclusive or enhanced for XAC in pathogenicity non-inducing condition, as shown by an additional proteomic analysis performed in this work between XAC and XauB in NB medium. Using the same extraction, separation protocols, and data analysis described for the XAM-M medium, we identified proteins from differential spots between XAC and XauB in NB medium which are presented in S3 Table.

The numbers of non-redundant proteins identified in XAC and/or XauB differential 2D spots for XAM-M and NB media (Table 1 and S3 Table, respectively) are summarized in S4 Table.

Among the 12 proteins identified from XAC spots in XAM-M, PGM (XAC3579) and TolC (XAC3463) are categorized as belonging to the annotated function Class VII (*Pathogenicity*, *Virulence and Adaptation*) (Table 1). TolC, predicted to be located in the outer membrane, was the only one that presented just one peptide matched, among other seven proteins from the same spot (spot 3, Table 1). PGM, predicted to be located in the periplasmic fraction, presented the highest values for its Mascot score, number of matched peptides, and sequence coverage among all the proteins identified in XAC spots (spots 1–4, Table 1).

### Blast searches for XI genes in XAC and XauB genomes at NCBI

XAC has two ORFs annotated as XI (XAC1776 and XAC4225), which are 99% identical and found to be at distinct genomic contexts and locations (Fig 7). On the other hand, XauB genome presents only a whole XI gene (XAUB_09030), which has 97% identity to the two XAC ORFs. Interestingly, when we performed a more detailed *in silico* analysis of the regions around XAC and XauB ORFs in the genome sequences at NCBI we found that in XAC only ORF XAC4225 has a putative *xyl-box* regulatory sequence located immediately upstream, which is known to be TGGTAGCGCTAACA according to Déjean *et al*. [64] for *X*. *campestris* (Fig 7). A putative *xyl-box* was also found upstream the ORF XAUB_09030 in XauB (Fig 7).

### Validation of PGM and XI differential expression in XAC and XauB by Western blot

**Fig 3:**
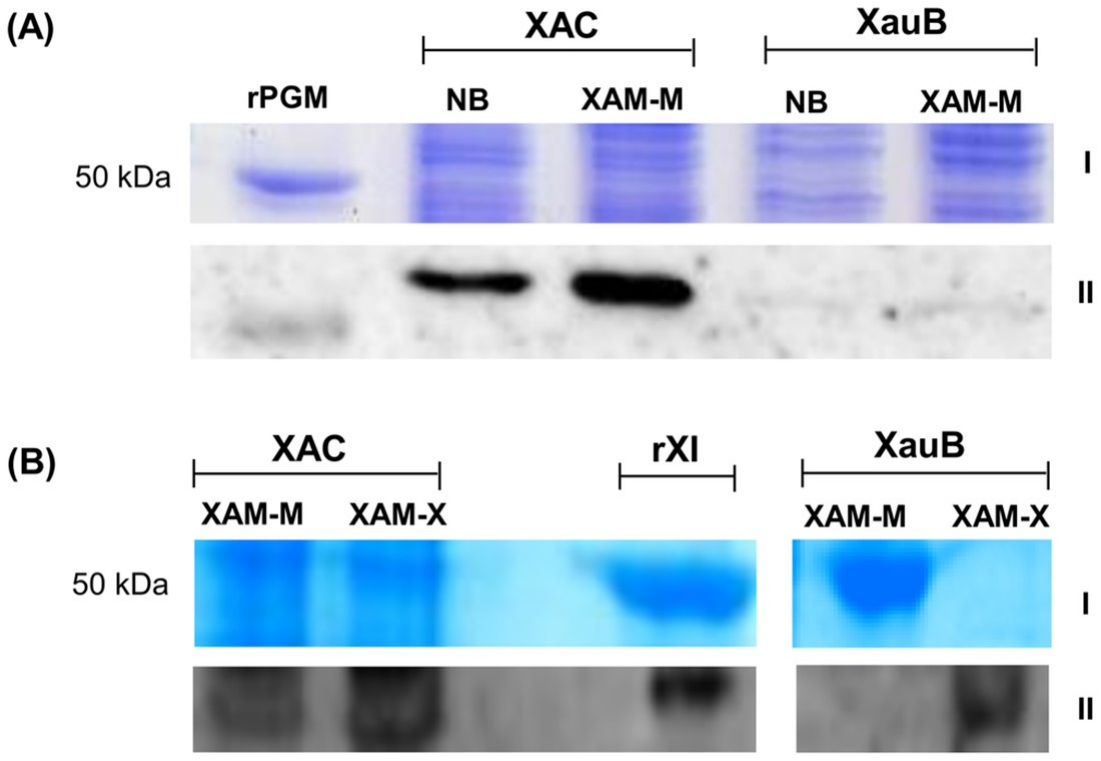
Immunodetection of PGM and XI in *X*. *citri* (type A) and *X*. *fuscans* (type B). Proteins (60 and 20 μg respectively for A and B) from the periplasmic-enriched fraction of XAC and XauB strains grown in NB (pathogenicity non-inducing medium), XAM-M (pathogenicity inducing medium), or XAM-X (XAM-M containing 10 mM of xylose) were separated by SDS-PAGE 12% and expression was analyzed by immunoblot with antibodies raised against XAC recombinant proteins PGM (rPGM) (A) or XI (rXI) (B). I and II correspond to results obtained by SDS-PAGE, after staining with Coomassie (A) or Silver Blue (B), and immunodetection of PGM (A) or XI (B), respectively. Molecular mass marker (M) position is indicated (50 kDa).

A Western blot was performed in order to demonstrate the differential expression of PGM and XI in the periplasmic-enriched fractions of XAC and XauB cells, using polyclonal antibodies raised against XAC recombinant proteins of PGM or XI. The results showed that PGM is constitutively expressed in XAC for the conditions tested (Fig 3A and S1C Fig), being up-regulated and/or more targeted to periplasm upon *in vitro* infectious condition. Results also showed that expression of PGM (Fig 3A and S1C Fig) and XI (Fig 3B and S1D Fig) is preponderant in XAC and that XI expression in XauB is dependent on xylose (XAM-X, see Methods).

## Discussion

To investigate new insights into XAC metabolism in *in vitro* infectious condition we performed differential proteomic analysis of the periplasmic-enriched fractions of XAC and XauB, once XauB causes cancrosis, a milder form of the citrus canker caused by XAC. Comparison of the 2DE profiles under *in vitro* pathogenicity- inducing conditions revealed remarkable differences between the profiles of the two bacteria.

When XAC and XauB were grown in XAM-M, only four differential spots for XAC were detected, whereas at least 4 times more differential spots were detected for XauB, which presented a more complex and wider set of proteins in terms of metabolic pathways (Table 1). This could be an indication that XAC is able to perform a more efficient adaptation to the *in vitro* infectious condition than XauB. Proteins identified from XAC were not detected in XauB (Table 1). Among the proteins identified for XAC were PGM and lytic murein transglycosylase, which were previously found in infectious XAC cells [5]. Succinyl-CoA synthetase subunit α, a protein also identified here for XAC in XAM-M (spot3, Table 1), has been previously found as three times more in infectious XAC cells [5].

Most of the proteins detected here in the periplasmic-enriched fraction are predicted to be cytoplasmic (Table 1). This feature is something expected for an enriched periplasmic fraction and was exhaustively discussed by Artier and co-authors [5], whose extraction methodology was similar to the method utilized in this work. The authors suggested that cytoplasmic proteins having additional or alternative *moonlighting* function in the periplasm may be involved in XAC pathogenicity [5]. Yet, the presence of some proteins in periplasm may be transient, as part of a route from the cytoplasm to the cell surface or even extracellular milieu.

This phenomenon of intracellular/surface moonlighting proteins has been observed widely in bacteria. Bacteria (and other pathogens) commonly use moonlighting cytosolic proteins on the cell surface for forming and maintaining interactions with the host species [22]. Non-classical surface proteins have been reported to function as putative adhesins in *Streptococcus pneumoniae* [23]. An increasing number of works has shown that chaperones and other cytoplasmic proteins involved in central metabolic pathways, such as Hsp60/GroEL, DnaK, glyceraldehyde 3-phosphate dehydrogenase, enolase, and fructose 1,6-bisphosphate aldolase have moonlighting function in the extracellular milieu and/or cell surface [24–28]. This was also suggested for some of them in XAC [23]. Unconventional mechanisms by which some cytoplasmic proteins are transported outside the cell are not yet understood. Recently, it was demonstrated for *Candida albicans* that most of the secreted proteins that lack signal peptide are transported to the extracellular environment by extracellular vesicles (EVs) [29]. As discussed by these authors, this microorganism’s EVs traffic can explain the presence of moonlighting proteins in both the extracellular medium and at the cell wall. However, more investigation is needed for a better understanding of these processes in XAC.

### XAC proteins in XAM-M culture

**Fig 4:**
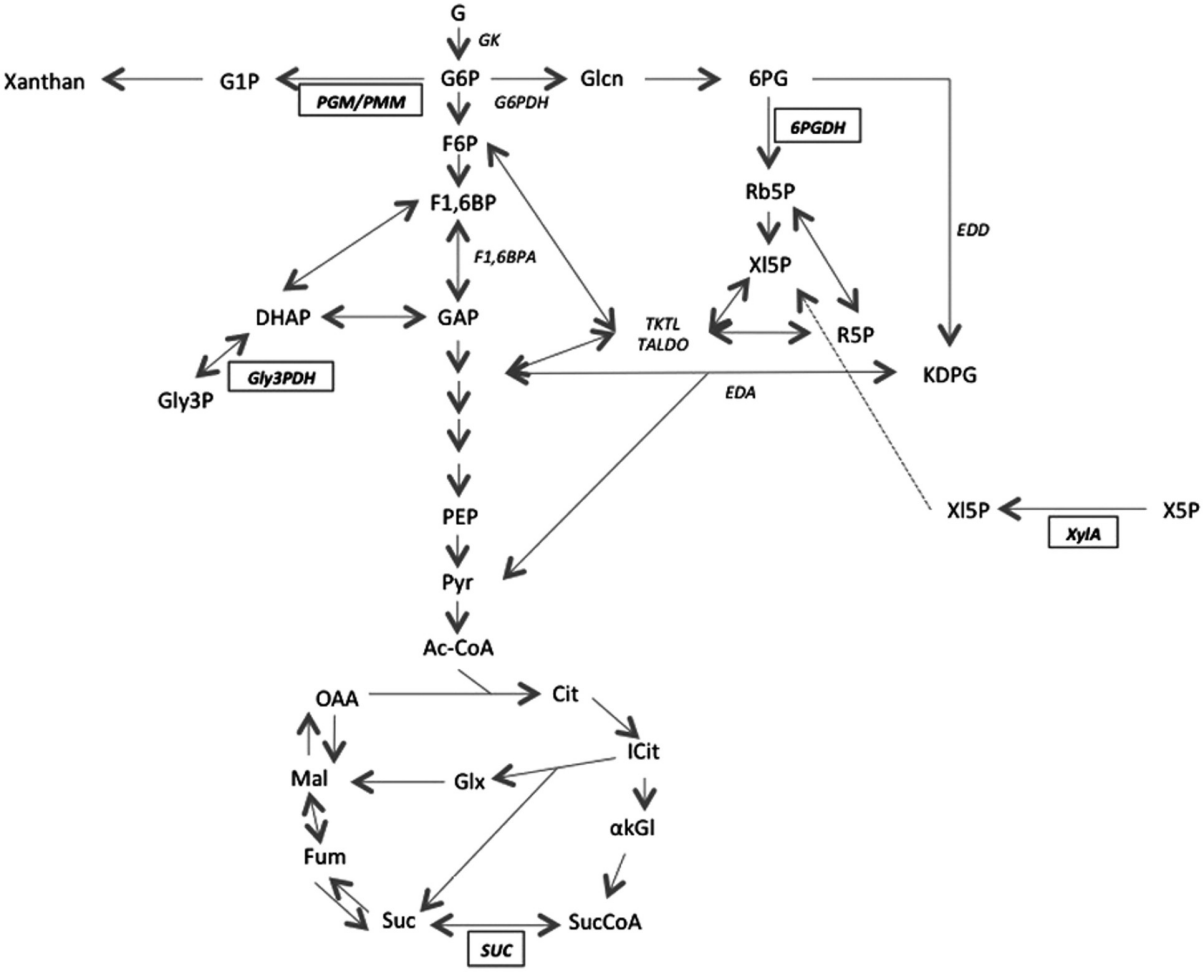
Pathways of the central carbon metabolism related to the proteins differentially identified for XAC in XAM-M relatively to XauB. Representations are according to the KEGG PATHWAY database (Kanehisa Laboratories, Japan). The names of five enzymes found in XAC (PGM/PMM, Gly3PDH, 6PGDH, XylA, and SUC, Table 1) are presented in bold inside a rectangle; other enzyme names are represented in italics. Abbreviations are: Gk–glucose kinase; PGM/PMM–phosphoglucomutase / phosphomannomutase; G6PDH–G6P dehydrogenase; F1,6BPA–F1,6BP aldolase; 6PGDH– 6PG dehydrogenase; Gly3PDH–glycerol-3-phosphate dehydrogenase; EDD– 6PG dehydratase; EDA–KDPG aldolase; XylA–XI; SUC–succinyl coenzyme A synthetase; TKTL–transketolase; TALDO–transaldolase. G–glucose; G6P –glucose-6-phosphate; G1P –glucose-1-phosphate; F6P –fructose-6-phosphate; F1,6BP–fructose-1,6-biphosphate; GAP–glyceraldehyde-3-phosphate; DHAP–dihydroxyacetone phosphate; Gly3P– glycerol-3-phosphate; Glcn–Gluconate; 6PG– 6-phosphogluconate; Rb5P –ribulose-5-phosphate; Xl5P –xylulose-5-phosphate; X5P –xylose-5-phosphate; R5P –ribose-5-phosphate; KDPG– 2-keto-3-deoxy-6-phosphogluconate; PEP–phosphoenolpyruvate; Pyr–pyruvate; AcCoA–acetyl coenzyme A; Cit–citrate; Icit–isocitrate; akGl–alpha-ketoglutarate; SucCoA–succinyl-coenzyme A; Suc–succinate; Fum–fumarate; Mal–malate; OAA–oxaloacetate.

A proposed metabolic scheme regarding some identified XAC proteins is presented in Fig 4 and is mainly related to their known classical functions, as discussed below. We cannot discard the possibility that these proteins maintain their enzymatic activity independently of the cellular compartment in which they are localized.

**Glucose-6-phosphate dehydrogenase** (6PGDH) (XAC0680) is encoded by the *gndA* gene and catalyzes the conversion of 6-phosphogluconate (6PG) into ribulose-5-phosphate entering PPP (Pentose Phosphate Pathway) in the cytoplasm. The uptake of extracellular glucose in *X*. *campestris* pv. *campestris* (Xcc) relies on two systems: one cytoplasmic, starting with glucose kinase (GK) and followed by G6PDH, and another periplasmic starting with NAD(P)+ independent glucose dehydrogenase (GDH), which directly produces gluconate. In the first pathway, G6P was reported to be catabolized also for exopolysaccharide production in Xcc [30]. Gluconate produced in both pathways is phosphorylated by gluconate kinase (GlcK) into 6-phosphogluconate (6PG) and then it may enter into the PPP through 6PGDH or directly into the Entner-Doudoroff (ED) pathway to be converted into glyceraldehyde-3-phosphate (GA3P) and pyruvate [30]. The ED pathway is the primary route for glucose catabolism in *X*. *oryzae* pv. *oryzae* (Xoo) [31] and is obligatory in other bacteria like *Vibrio cholera*, besides being determinant of its pathogenicity [32]. Schatschneider and colleagues also confirmed the prevalent glucose catabolic role of the ED in Xcc and minor fluxes of carbon through PPP and Embden-Meyerhof-Parnas (EMP) pathways. Although smaller, an important carbon flux through PPP was assessed in minimal medium [33]. Therefore, considering the classical role of 6PGDH, its presence in the periplasm-enriched fraction of XAC cells grown in XAM-M suggests a possibly more active PPP in XAC than in XauB.

**Fig 5:**
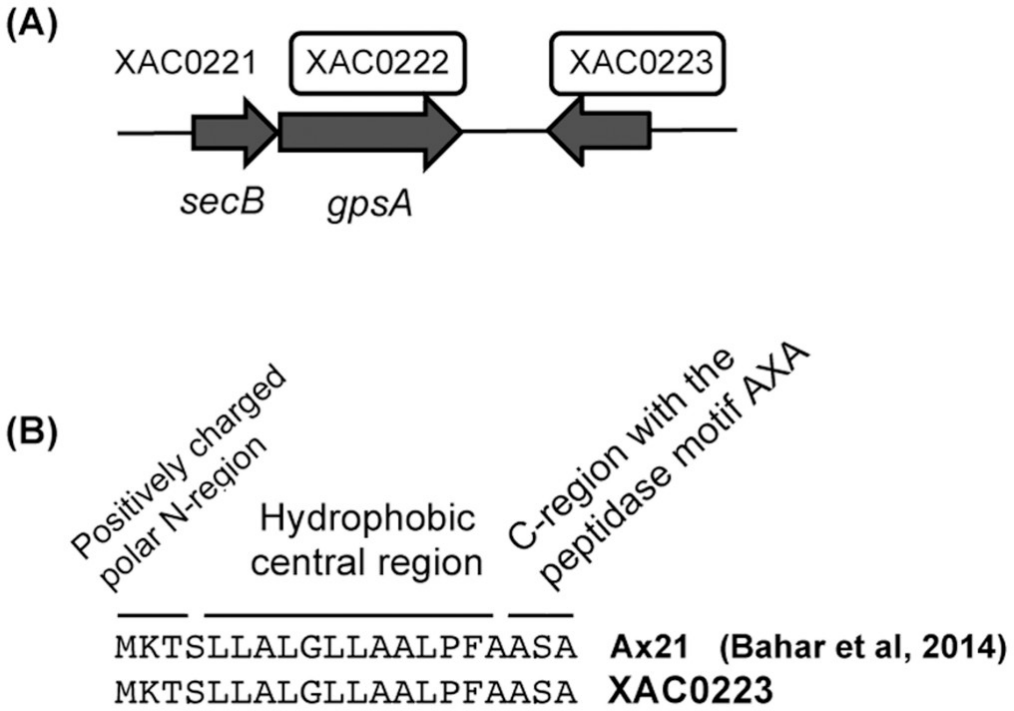
XAC genomic organization for NAD(P)H-dependent glycerol-3-phosphate dehydrogenase (GpsA) and XAC0223 hypothetical protein. GpsA (XAC0222) and XAC0223 hypothetical protein (underscored by boxes) were both identified in the periplasm-enriched fraction of XAC grown in XAM-M. The sequence of preprotein translocase subunit secB, designated as XAC0221, is shown in close association with *gpsA* **(A)**. Also, a signal peptide found in XAC0223 **(B)** is the same as Ax21 protein from *Xoo*.

**The NAD(P)H-dependent glycerol-3-phosphate dehydrogenase** (XAC0222) (GpsA) is the first committed enzyme of polar lipid metabolism, catalyzing the interconversion between dihydroxyacetone phosphate (DHAP) and glycerol-3-phosphate (G3P) in the cytoplasm of both bacteria and eukaryotes [34] (Fig 4). Although one copy of *gpsA* is present in both XAC and XauB genomes (with 98% identity), GpsA was detected in periplasm-enriched fraction only for XAC cells grown in XAM-M. The presence in periplasm is expected due to a predicted signal peptide of XAC0222 ORF (Table 1). The close proximity of *gpsA* (XAC0222) and *secB* (preprotein translocase subunit SecB gene, XAC0221) (Fig 5A) in both the XAC and XauB genomes (only 16 and 18 nucleotides separates the two sequences in the respective genomes) suggests that, if both sequences are part of an operon, GpsA could have a *secB*-dependent translocation. Curiously, the coding region of *gpsA* has been found to begin within the termination codon of *secB* in *Escherichia coli* [35]. The fact that GpsA has been detected in XAC’s periplasm in XAM-M culture suggests that lipid metabolism pathway at periplasm may be altered in infectious XAC cells.

**Hypothetical protein XAC0223,** a low molecular mass protein of approximately 20 kDa (Table 1), located near *secB* and *gpsA* in both the XAC and XauB genomes (but on the complementary strand, Fig 5A), has a nucleotide sequence that shares 94% identity with the Xoo Ax21protein (PXO_03968) [36] and codifies an N-terminal Sec signal peptide [37] (Fig 5B). Sec-targeted proteins are delivered to the periplasm by SecB, a secretion specific chaperone [38]. Ax21 protein has been actually found in the culture medium of *Xoo* as a mature protein, but not as a soluble secreted protein [37]. In Xoo, Xcc, and *Xanthomonas euvesicatoria* (Xcv), Ax21 was associated exclusively with the outer membrane vesicles (OMV), suggesting that the secretion of Ax21 via the OMV secretory pathway is conserved among these *Xanthomonas* species; however, the biological function of Ax21 remains to be determined [37].

A large number of Gram-negative bacteria, including *Xanthomonas* spp., constitutively secrete OMVs [39, 40], which requires constant polar lipids synthesis. Here, the protein encoded by XAC0223 was identified by MS in cells of XAC grown in XAM-M. Taking into account all these considerations, it seems very probable that XAC0223 is an outer membrane protein (Table 1), a homolog of the Ax21 protein, targeted to the periplasm by the Sec system, and released in OMVs by XAC. To confirm this hypothesis further investigation is required. The XAC0223 protein has recently been reported as a XAC secreted protein under *in vitro* pathogenicity induction by XAM-1 medium and characterized as a PAMP (Pathogen- Associated Molecular Patterns) [41]. For all these reasons, investigation about the involvement of XAC0223 protein in the XAC pathogenicity is underway in our research group. Interestingly. the genomic organization of the ORFs XAC0221, XAC0222, and XAC0223 in the XAC genome is similar to the organization found for XauB’s genome, which presents all the respective homologous ORFS (XAUB_35480, XAUB_35470, and XAUB_35460) (not shown).

**Hypothetical protein XAC0901** is another low molecular mass protein of approximately 20 kDa (Table 1) detected in XAC grown in XAM-M (Table 1). One copy of XAC0901 was found in both the XAC and XauB genomes (96% identity). A Blast search has also revealed it has strong homology with peptidases of *Xanthomonas* spp. (not shown). However, its biological role in XAC remains not understood.

**Succinyl-CoA synthetase** (SUC) β-subunit (SucD) (XAC3236), encoded by the *sucD* gene (presented as one copy in both the XAC and XauB genomes with 98% sequence identity, and in close proximity with the *sucC* gene), was also only found in an exclusive spot for XAC in XAM-M (Table 1). Although the α-subunit of the enzyme was not detected in this work (XAC *vs* XauB in XAM-M), it was observed in the periplasm-enriched fraction of XAC cells at almost three times higher when XAC cells were grown in XAM-M than in NB [5]. This tricarboxylic acid (TCA) cycle enzyme, a 140 kDa SucCD heterotetramer in Gram-negative bacteria, catalyzes the conversion of succinyl-CoA into succinate (reversible in the presence of coenzyme A) in cytoplasm (Fig 4), the only TCA cycle step where nucleotides are generated through phosphorylation at the substrate level. The reaction renders either ATP or GTP, together with succinate. While α-subunits of succinyl synthetases (Scs) heterotetramers in Gram-negative bacteria become transitorily phosphorylated in the reaction, β-subunits has specificity for ADP or GDP as phosphate receptors [42]. Intracellular concentrations of ADP and GDP modulate the interconversion of ATP and GTP by Scs [43]. In this work, the reason for the presence of SucD in the periplasm-enriched fraction of XAC in XAM-M, but not for XauB, is not clear. However, it is worth noting that the conversion step of succinyl-CoA into succinate is able to regulate purine biosynthesis directly through regulation of ATP to GTP ratio, synthesizing GTP that is directly used for the conversion of aspartate into adenylosuccinate by adenylosuccinate synthetase, an enzyme also found in XAC (Table 1) and discussed as follows.

**Adenylosuccinate synthetase** (S-AMPS) (XAC1158) is encoded by *purA* and plays an important role in *de novo* purine biosynthesis in the cytoplasm, producing adenylosuccinate (S-AMP) by linking GTP hydrolysis to condensation of inosine monophosphate (IMP) with L-aspartate (Asp) [44]. The purine biosynthesis pathway is tightly connected to several points of the central metabolism, especially to the TCA cycle, through GTP at the level of Scs, and is tightly regulated [45]. Simultaneous detection of Scs and S-AMPS in XAM-M for only XAC seems to indicate the dependence of infectious XAC cells on *de novo* purine biosynthesis, probably due to the scarcity of these compounds in the host apoplast. Mutants of *Salmonella enterica* Serovar Typhimurium, incapable of converting α-ketoglutarate into succinyl-CoA and unable to convert malate into pyruvate and oxaloacetate, have been reported to be avirulent and immunogenic in mice [46].

**Lytic murein transglycosylase** (LMT) (XACb0007), encoded by the *mlt* gene, was only detected in XAM-M for XAC (Table 1). LMT expression in XAC has been reported to be highly increased under *in vitro* pathogenicity induction [5]. LMT is probably an inner membrane-targeted protein as predicted by cellular location analysis that also evidenced the presence of a signal peptide (Table 1). LMT has been found in multiple distinct spots [5], evidencing the possibility of isoforms. This enzyme is involved in peptidoglycan metabolism and could be responsible for rearrangements of the XAC cell wall during host infection [5].

**Enolase** (ENO) (XAC1719) is classically known to catalyze the reversible conversion of glycerate-2-P into phosphoenolpyruvate (PEP) at the near end of the glycolytic pathway, and is therefore expected to be found in the cytosol (Table 1). In this work, enolase was exclusively detected in XAC (Table 1). Although it has been reported as a glycolytic enzyme with participation in host-pathogen interactions in other organisms [47–50], it is still not possible to establish their relation with XAC pathogenicity and/or its possible periplasmic location in this bacterium. Increasing evidences for ENO as a *moonlighting* protein has been reported in several organisms [51]. Additionally, Artier and co-authors detected a decrease of enolase-phosphatase (XAC1838) in XAM-M in relation to NB medium, which could suggest the involvement of enolase and a role of post-translational modifications of this enzyme during XAC infection process [5]. ENO has conserved phosphorylated residues from *Archaea* to humans, as well as EF-Tu and PGM/PMM [52].

**Elongation factor Tu** (EF-Tu) (XAC0957) is involved in the elongation of nascent polypeptides during protein translation but is also involved in catalyzing disulfide formation and reduction, like thioredoxin [53], and presents some chaperone properties. Under *in vitro* pathogenicity induction, EF-Tu was only found in XAC (Table 1). EF-Tu has been reported to be transported from *E*. *coli’s* cytoplasm to periplasm upon osmotic shock through the large-conductance mechanosensitive channel (MscL) remaining trapped there. As reported by Ferreira and co-authors, EF-Tu, together with the above discussed Ax21 protein, has been characterized as PAMP, being capable of inducing a PTI (PAMP -triggered immunity response), which can increase the synthesis of chorismate, involved in the ROS response and induction of plant defense [41]. A diversity of additional features has been attributed to this protein including association to outer membrane vesicles [54], interaction with a specific protein receptor of *Arabidopsis* [55], and involvement in XAC biofilm [56]. Ferreira *et al*. [41] hypothesized that the secretion of this protein may be associated with T3SS in *Xanthomonas*. Ef-Tu has also been reported as a moonlighting protein [57].

**Phosphoglucomutase/phosphomannomutase** (PGM/PMM) (XAC3579), encoded by *xanA*, was found in a XAC unique spot (Table 1). This enzyme diverts hexose 6-phosphates (like G6P) from central metabolism to G1P for the biosynthesis of xanthan (Fig 4) and lipopolysaccharides (LPS) in Xcc [58]. PGM/PMM could work like a valve, rerouting the metabolic flux originating from hexose phosphates either toward the biosynthesis of LPS or xanthan, or the generation of energy or building blocks, such as amino acids, for cellular growth [33]. It also catalyzes other reactions related to central carbon metabolism, namely conversion of mannose-6-phosphate (M6P) to mannose-1P (M1P); ribulose-5P (R5P) from PPP to R1P, which is deviated to purine metabolism. However, PGM/PMM is considered a key enzyme in nucleotide sugar synthesis, as mutations in the *xanA* gene cause defects in the synthesis of both xanthan and LPS [59]. Its exclusive detection in XAC suggests that it may play a singular role in XAC performance. Furthermore, PGM/PMM was also previously detected at higher levels in XAC grown in XAM-M relatively to NB [5]. Its functional characterization and involvement with XAC pathogenicity have recently been reported by comparing *in vivo* infectiveness of a XAC mutant, obtained by PGM/PMM deletion, to the XAC wild strain [21].

In this work, the differential proteomic analysis between XAC and XauB periplasmic-enriched fractions detected PGM in XAC. Western blot analysis using antibodies raised against the XAC recombinant PGM showed that PGM expression is predominant in XAC (Fig 3A). Although XauB also has a PGM gene with 98% identity to the XAC homolog, it seems that PGM expression is more prominent in XAC than in XauB (Fig 3A).

**TolC** (XAC3463), an important low-abundance protein in the outer membrane of gram-negative bacteria, functions as a component of multidrug resistance (MDR) efflux systems in the removal of toxic chemicals from the cell [60]. Here, TolC was detected only for XAC in XAM-M, being one of only two proteins classified as belonging to Class VII. In XAC, it is predicted to be an outer membrane protein and to have a putative signal peptide (Table 1). TolC has been reported to be essential for phytopathogenesis since it is involved in resistance to antimicrobial plant chemicals in the plant pathogenic bacteria *Erwinia chrysanthemi* [60]. TolC was shown to be both functional and necessary for pathogenicity and, probably, *in planta* survival of *X*. *fastidiosa*, since mutagenesis of the single tolC gene in the Pierce´s disease strain of Temecula resulted in a total loss of pathogenicity on grapes. Additionally, *tolC* mutant strains were not recovered after inoculation into grape xylem, strongly indicating that multidrug efflux is critical for the survival of this fastidious pathogen [61].

#### The special case of XI

XI (XAC1776) was detected in the periplasm-enriched fraction of XAC grown in XAM-M (Table 1). This enzyme catalyzes the conversion of D-xylose to D-xylulose. D-xylose is a monomer that composes xylan and xyloglucans, major hemicelluloses of the plant cell wall. The enzyme product, D-xylulose, can be phosphorylated by xylulokinase and enters PPP [62] (Fig 4). Plant pathogenic bacteria are known to express enzymes with xylanolytic activity that helps them to break plant cell walls in order to obtain nutrients during host invasion.

**Fig 6:**
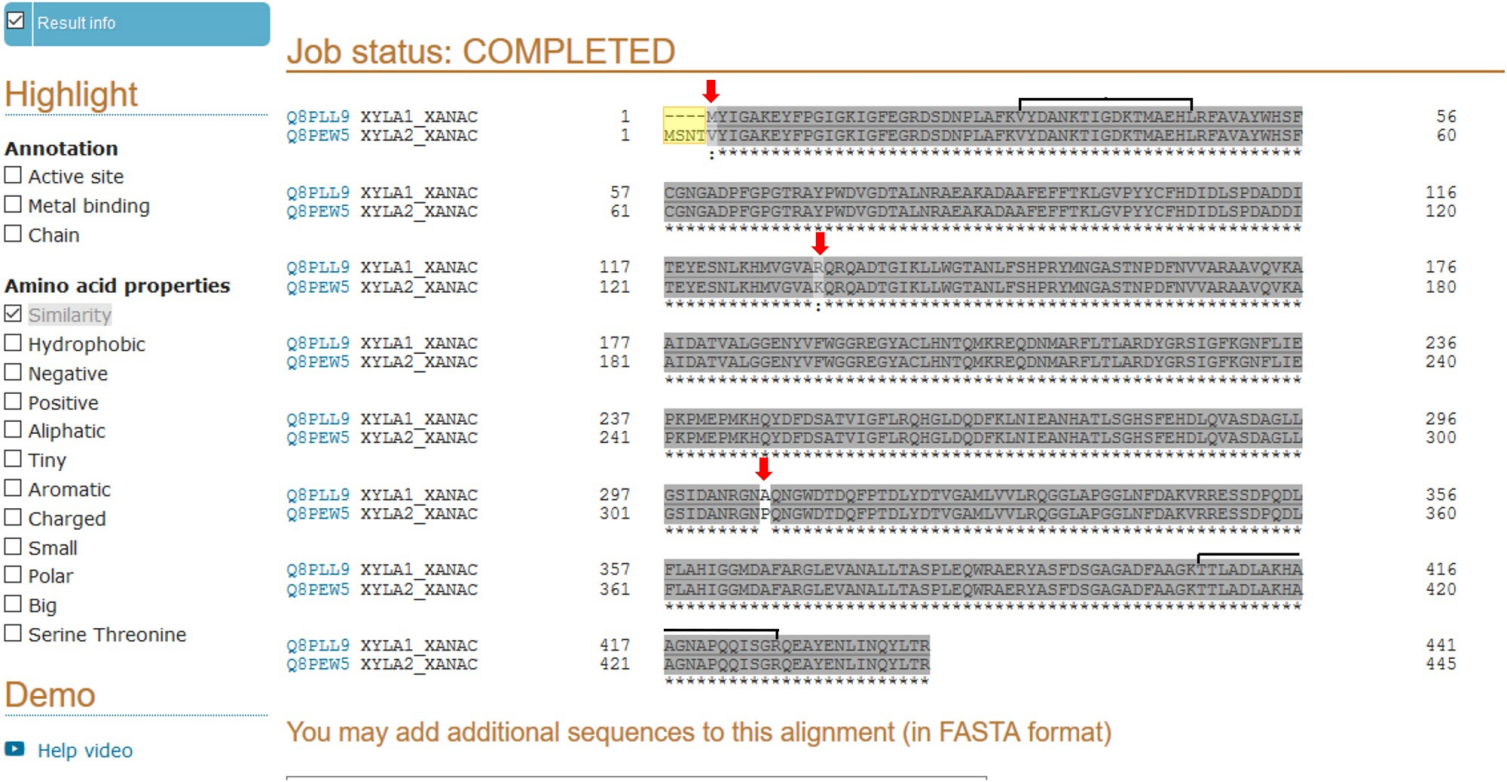
Sequences alignment between the two proteins of xylose isomerase codified by the ORFs XAC1776 (Q8PLL9) and XAC4225 (Q8PEW5) in XAC 306 according to the genome sequence and annotation at NCBI. Alignment was performed by the Uniprot tool [63]. Differences between them are indicated at N-terminal (in yellow) and by red arrows, and the regions of sequence identity of the two peptides obtained by MS-MS in this work (S1 Data), and utilized by Mascot for the xylose isomerase identification, are indicated by horizontal brackets.

As previously mentioned, XAC4225, another XI present in the XAC genome, is 99% identical to XAC1776. Fig 6 shows the alignment performed using the Uniprot tool [63] between the proteins codified by the ORFs XAC1776 and XAC4225 from XAC 306 database, where only slight differences between them were found. Two peptides, sequenced by MS-MS for xylose isomerase identification (S1 Data), showed identical sequences for the two proteins (Fig 6). Therefore, the two proteins codified by the ORFs annotated as xylose isomerase (*xyl*A) in the XAC306 genome at NCBI (XAC1776 and XAC4225) could not be distinguished unambiguously by the MS-MS analysis performed in this work. Investigation is ongoing in our lab in order to understand if these ORFs are differentially regulated in XAC.

**Fig 7:**
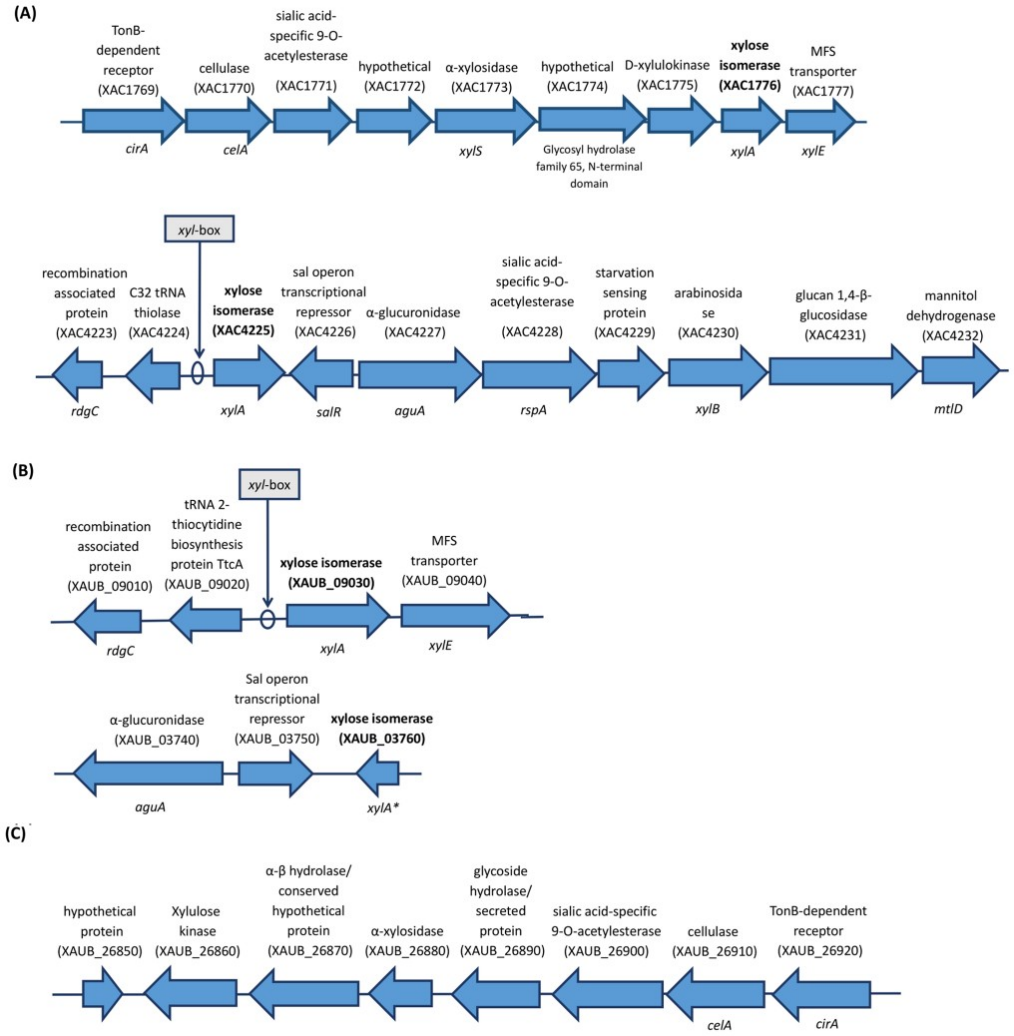
Representation of the annotated genes in XAC 306 and XauB ICPB11122 genomes at NCBI for the genomic contexts of *xylA* genes (in bold). The indicated putative *xyl-box* sequences were found by *in silico* analysis performed in this work. Genomes database investigated were from: A) XAC; B) and C) XauB.

The genomic contexts of the xylA genes in XAC are very distinct, as observed for their regions in XAC genome at NCBI, represented in Fig 7A. A XI gene is also annotated in XauB genome at NCBI (XAUB_09030, Fig 7B), but unlike XAC the correspondent protein was not detected for XauB in XAM-M (Table 1). At NCBI, the genome of XauB contains one additional ORF annotated as a XI, XAUB_03760, whose gene product was also not detected by the proteomic analysis reported here. Blast searches showed that only XAUB_09030 is similar to the two XAC ORFs (97% identity), whereas XAUB_03760 (xylA*, Fig 7B) merely corresponds to a partial sequence of XAUB_09030 (697–1338 nucleotide position). These two XauB ORFs are also inserted in distinct genomic contexts, as shown in the XauB genome sequence at NCBI (Fig 7B). Finally, one more XI-related sequence was found in the XauB genome (XAUB_26850, Fig 7C). It is annotated as a hypothetical protein, however, a BlastN analysis revealed this sequence to correspond to a partial sequence (first 393 nucleotides) of XAUB_09030. XAUB_26850 is inserted in a gene cluster (Fig 7C) very similar to the one found in XAC for XAC1776, except for MFS transporter gene (XAC1777, Fig 7A) which is missing in the XauB cluster. It is not clear if XAUB_26850 is actually a truncated XauB XI or if these traits could be attributed to incomplete information due the draft genome database status of XauB [3].

*Xanthomonas campestris* pv *campestris* (Xcc) also presents two XI encoded by the *xylA1* and *xylA2* genes located in two loci, *xylE* and *xylR*, respectively [64]. The expression of the *xylR* locus was reported as specifically induced by xylo-oligosaccharides [65], due to a *xyl*-box motif sequence (TGGTAGCGCTAACA), unlike the *xylE* locus, which does not present a *xyl*-box, even imperfect or degenerated [64]. The expression of *Xcc*’s *xylA2* is repressed by XylR because it possesses a *xyl*-box, being the only XylR-repressed gene in the *xylR* locus. Similarly, *in silico* analysis of the XAC and XauB gene sequences performed in this work revealed that only one of the two XI ORFs found in either XAC or XauB has a *xyl*-box sequence: XAC4225 in XAC (Fig 7A) and XauB_09030 in XauB (Fig 7B). Further investigation is underway in our group in order to perform functional characterization of the two XI ORFs found in XAC.

Additionally, the results of the Western Blot showed that XI expression in the periplasm-enriched fraction of cells grown in XAM-M was detectable only for XAC, unless xylose is added (XAM-X, Fig 3B).

Taking all these considerations together it is a possibility that XAC is capable of an efficient adaptation to the infectious condition by switching a few strategic points of its carbon metabolism in order to prepare the cell to take advantage of the additional carbon sources from the host environment, however, more investigation is necessary to support this hypothesis. Xanthan synthesis and other carbon-demanding processes, for example OMV synthesis, may also be associated with the increased virulence of XAC, whereas these features seem to be less efficient or even absent in XauB.

## Conclusion

Comparison of the periplasmic-enriched proteomes of XAC and XauB was a useful approach to gain insight into the pathogenicity of XAC. Periplasmic-enriched fractions of XAC and XauB present very distinct proteomic profiles and some differential proteins were exclusive to or enhanced for XAC only in infectious conditions. Whereas exopolysaccharide synthesis (mediated by phosphoglucomutase) and other processes like lipid and protein metabolism for OMV synthesis are probably required during the infectious condition, XAC seems to also have the ability to take advantage of organic sources such as xylose, available from the host during plant invasion. Other features could also have a role in XAC pathogenicity, such as efflux systems for toxic compounds, the involvement of *moonlighting* proteins, and post-translational modifications, whereas these features seem to be of little or no effect in XauB.

## Supporting information

S1 Fig **(A-B)** Protein profiles of the periplasmic-enriched fraction from XAC and XauB after separation by two-dimensional electrophoresis. The raw images correspond to each biological gel triplicate from cells grown in (A) pathogenicity inducing medium (XAM-M), and (B) pathogenicity non-inducing medium (NB) from Xac 306 (I) and XauB ICPB11122 (II). The gels were run using IPG strips of 13 cm and p*I* linear gradient of 3–10, as indicated, and were stained with Coomassie Brilliant Blue R-250. Molecular weight standard (Benchmark, Invitrogen) are indicated at the left side of the gels. The gels XAC XAM-M 44186 and XauB XAM-M 15563 correspond to the gels shown in Fig 2. **(C-D)** Immunodetection of PGM and XI in *X*. *citri* (type A, XAC) and *X*. *fuscans* (type B, XauB). Proteins (60 and 20 μg respectively for C and D) from the periplasmic-enriched fraction of XAC and XauB strains grown in NB (pathogenicity non-inducing medium), XAM-M (pathogenicity inducing medium), or XAM-X (XAM-M containing 10 mM of xylose) were separated by SDS-PAGE 12% and expression was analyzed by immunoblot with antibodies raised against XAC recombinant proteins PGM (rPGM) (C) or XI (rXI) (D). I and II correspond to results obtained by SDS-PAGE, after staining with Coomassie (C) or Silver Blue (D), and immunodetection of PGM (C) or XI (D), respectively. Molecular weight (MW) markers were High Range Rainbow RPN76E (C) and Precision Plus Protein^™^ Dual Color Standards (BIO-RAD) (D). Photo documentation was made using ChemicDoc^™^ Imaging System BIO-RAD. The rectangles delimit areas that correspond to the gels and blots shown in Fig 3.
(PDF)

S1 Data XAC proteins identified by mass spectrometry (p< 0.05) in XAM-M medium based on the XAC306 database at NCBI and presented in Table 1. Matched peptides are in bold/underlined. In parenthesis is the number of different peptides with the same sequence. Proteins that had a score above the required minimum score for identity or extensive homology (p<0.05) are shown here.
(PDF)

S2 Data XauB proteins identified by mass spectrometry (p< 0.05) in XAM-M medium based on the XauB database at NCBI and presented in Table 1. Matched peptides are in bold/underlined. In parenthesis is the number of different peptides with the same sequence. Proteins that had a score above the required minimum score for identity or extensive homology (p<0.05) are shown here.
(PDF)

S1 Table XAC proteins identified by mass spectrometry (p< 0.05) in XAM-M medium based on XAC306 database at NCBI and presented in Table 1 and S1 Data. The .dat files were open on Scaffold^™^ software to group the peptides identified according to Mascot parameters.
(XLSX)

S2 Table XauB proteins identified by mass spectrometry (p< 0.05) in XAM-M medium based on database at NCBI and presented in Table 1 and S2 Data. The .dat files were open on Scaffold^™^ software to group the peptides identified according to Mascot parameters.
(XLSX)

S3 Table Proteins identified by ESI-Q-TOF in spots exclusively presented by XAC or XauB cells grown in pathogenicity non-inducing medium (NB) for 25 h or 40 h, respectively, from periplasm-enriched fraction resolved on 2-DE (p< 0.05). XAC and XauB were grown in NB medium, and periplasm-enriched fractions, extracted from both bacteria, were resolved on 2-DE (p< 0.05), and proteins from differential spots were identified by ESI-Q-TOF. The cultivation times were for XAC 25 h and for XauB 40 h. ^a^ Proteins identified using Mascot with XAC or XauB databases (NCBI); not all XauB proteins identified were included, only those ones that presented the highest Mascot scores and/or molecular weight (MW) and isoelectric point (p*I*) more compatible with experimental values; ^b^ Exclusive peptides count determined for some spots using Scaffold^™^ software (Proteome Software Inc., Portland, OR) for 100% protein identification probability; ^c^ Theoretical MW and p*I* of the matched protein obtained from the NCBI database; ^d^ Experimental molecular weight (MW) and isoelectric point (p*I*) calculated by Image Master Platinum software (GE Healthcare) based on the position of the spot on 2-DE; ^e^ Proteins clustering according to “Xanthomonas axonopodis pv. citri Main Chromosome and Plasmid Gene List” at NCBI [6]: I) Intermediary metabolism, II) Biosynthesis of small molecules, III) Macromolecule metabolism, IV) Cell structure, V) Cellular processes, VI) Mobile genetic elements, VII) Pathogenicity, virulence and adaptation, VIII) Hypothetical, IX) ORFs with undefined category; ^f^ Predicted cellular location of proteins by pSortP 3.0 and SecretomeP 2.0. P, M, and C correspond respectively to periplasm, membrane, and cytoplasm location. Signal (+) indicates the presence of signal peptide according to SignalPeptide 2.0.
(PDF)

S4 Table Numbers of non-redundant proteins identified by MS-MS analysis in differential 2DE spots of the periplasm-enriched fractions of XAC and/or XauB after *in vitro* growth in XAM-M (pathogenicity-inducing) and NB (pathogenicity non- inducing) culture media. (PDF)

## Abbreviations

2-DE: 2-D electrophoresis
DDA: data-dependent acquisition
dpi: dots per inch
NB: Nutrient Broth
PGM: phosphoglucomutase/ phosphomannomutase
XAC: *Xanthomonas citri* subsp. *citri* type A
XauB: *Xanthomonas fuscans* subsp. *aurantifolii* type B
XauC: *X*. *fuscans* subsp. *aurantifolii* type C
XI: xylose isomerase
