# Supplementary figures and images for: Periplasm-enriched fractions from Xanthomonas citri subsp. citri type A and X. fuscans subsp. aurantifolii type B present distinct proteomic profiles under in vitro pathogenicity induction
Source: PLoS One. 2020 Dec 18;15(12):e0243867. doi: 10.1371/journal.pone.0243867 (PMC7748154; doi:10.1371/journal.pone.0243867)

A.

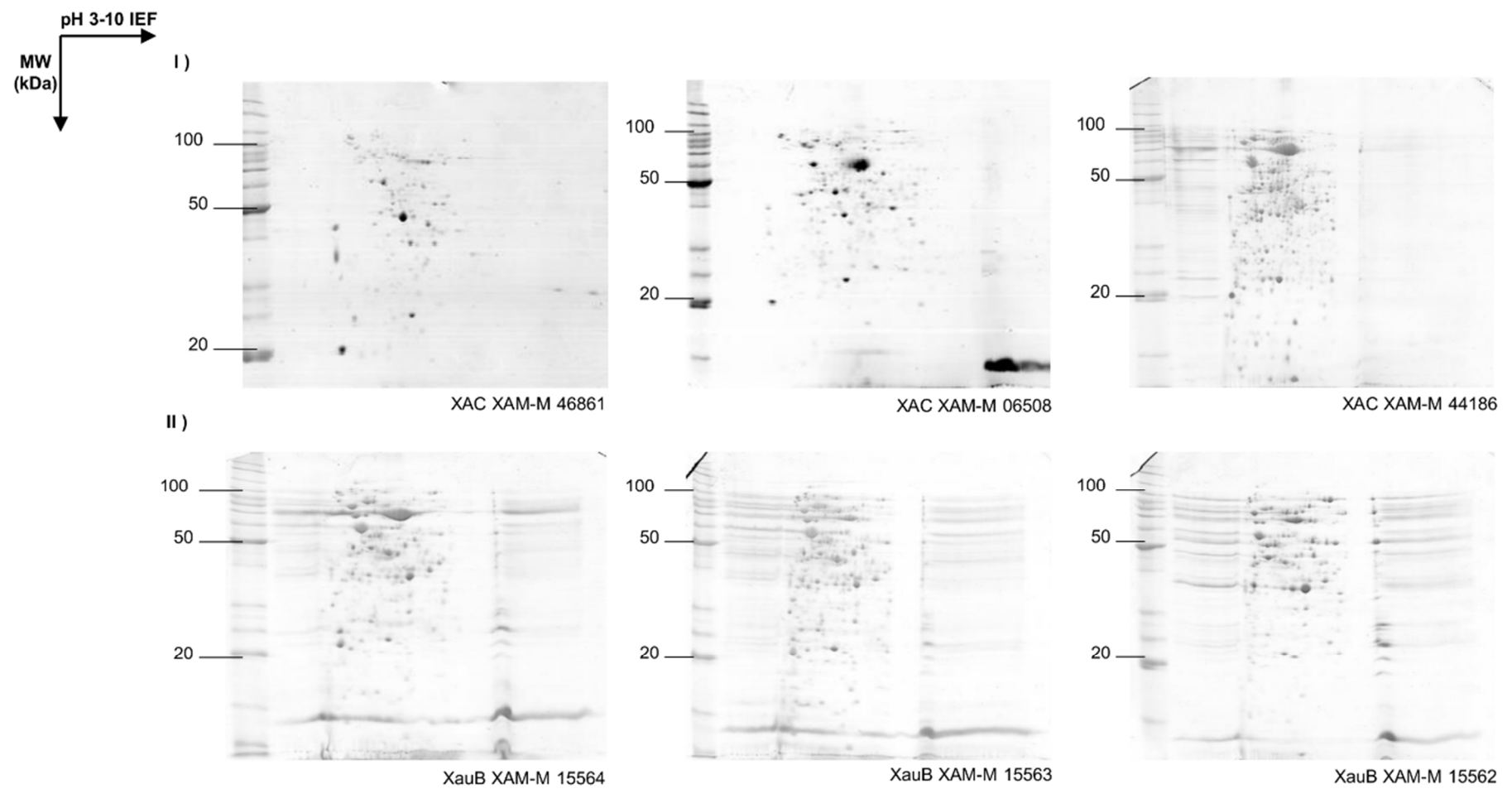

**B.**

**I)**

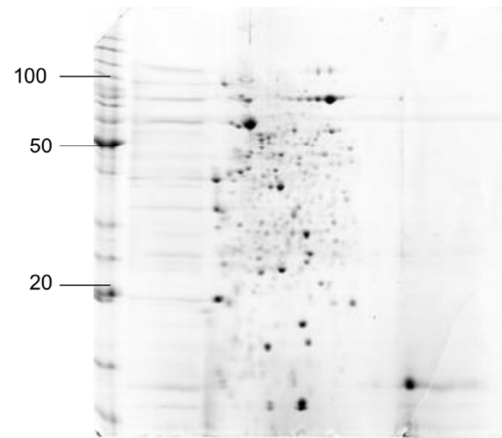

XAC NB 46856

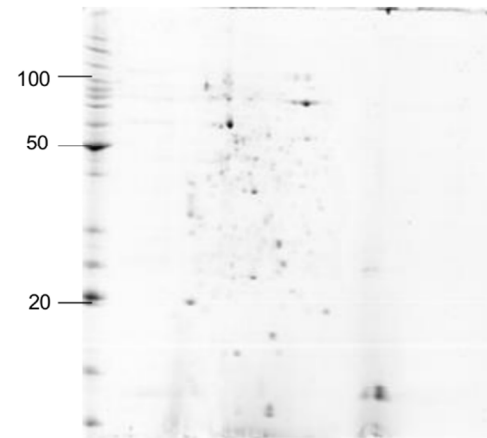

XAC NB 46832

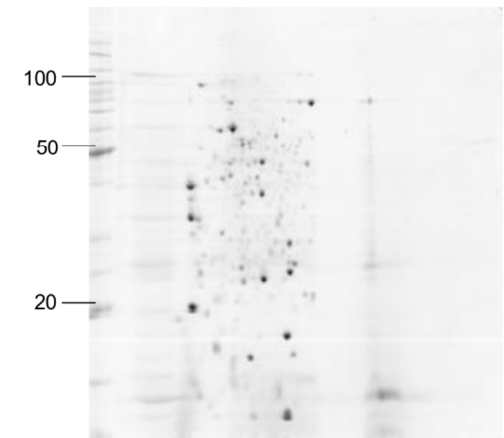

XAC NB 46860

**II)**

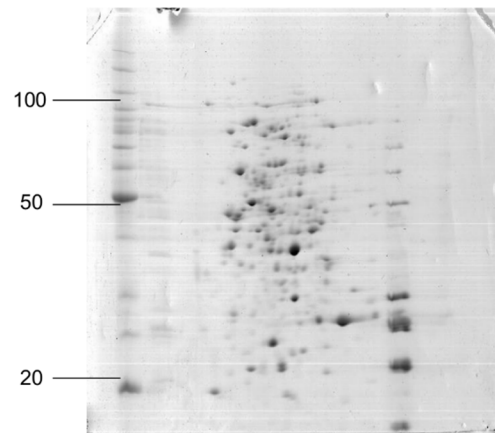

XauB NB 48290

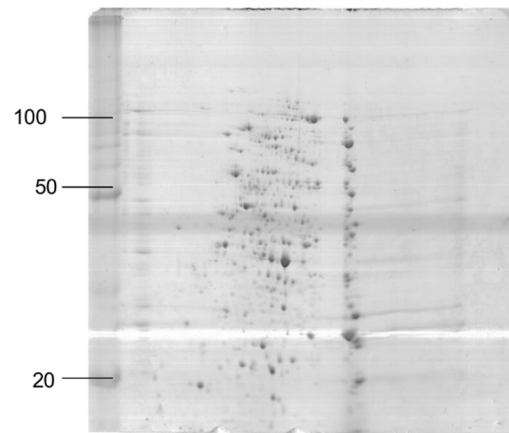

XauB NB 46824

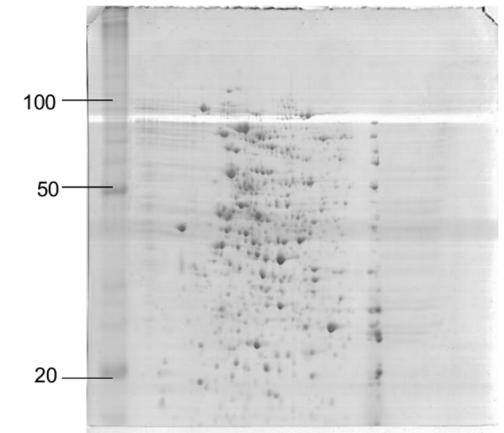

XauB NB 46823

**C.**

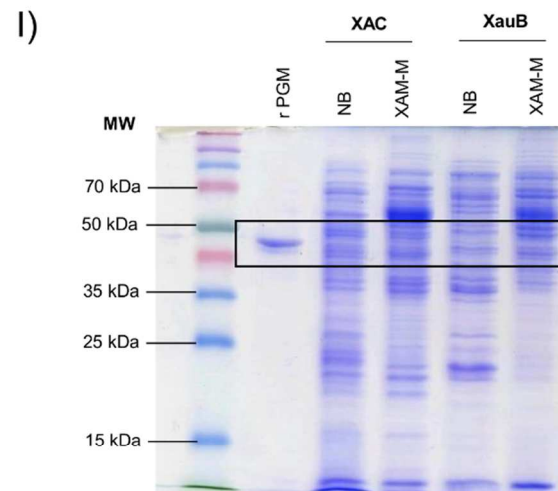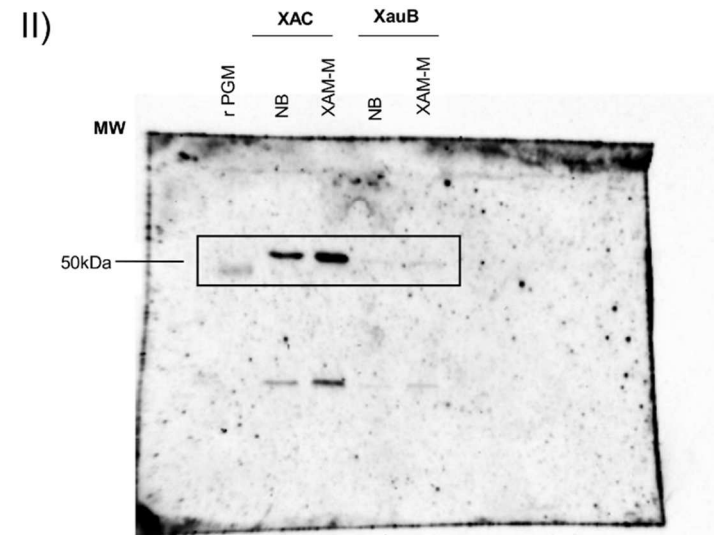

**D.**

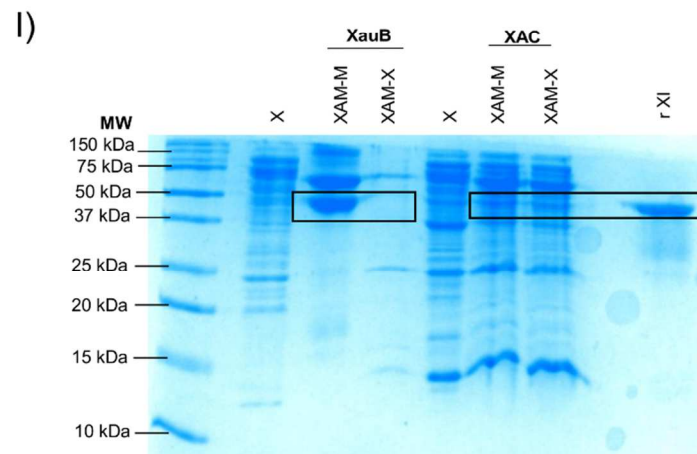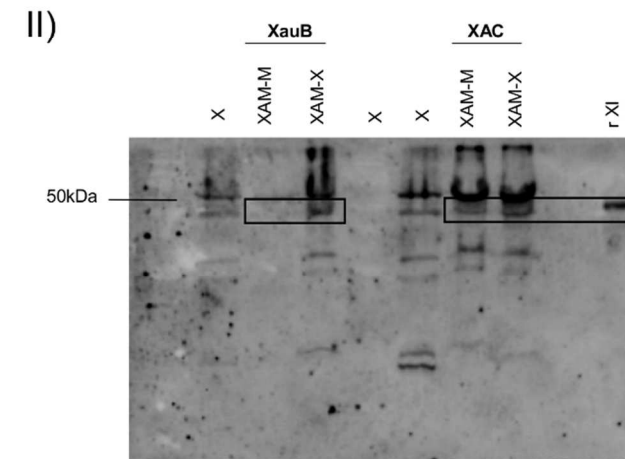

Supplement: S1 Fig — (A-B) Protein profiles of the periplasmic-enriched fraction from XAC and XauB after separation by two-dimensional electrophoresis. The raw images correspond to each biological gel triplicate from cells grown in (A) pathogenicity inducing medium (XAM-M), and (B) pathogenicity non-inducing medium (NB) from Xac 306 (I) and XauB ICPB11122 (II). The gels were run using IPG strips of 13 cm and pI linear gradient of 3–10, as indicated, and were stained with Coomassie Brilliant Blue R-250. Molecular weight standard (Benchmark, Invitrogen) are indicated at the left side of the gels. The gels XAC XAM-M 44186 and XauB XAM-M 15563 correspond to the gels shown in Fig 2. (C-D) Immunodetection of PGM and XI in X. citri (type A, XAC) and X. fuscans (type B, XauB). Proteins (60 and 20 μg respectively for C and D) from the periplasmic-enriched fraction of XAC and XauB strains grown in NB (pathogenicity non-inducing medium), XAM-M (pathogenicity inducing medium), or XAM-X (XAM-M containing 10 mM of xylose) were separated by SDS-PAGE 12% and expression was analyzed by immunoblot with antibodies raised against XAC recombinant proteins PGM (rPGM) (C) or XI (rXI) (D). I and II correspond to results obtained by SDS-PAGE, after staining with Coomassie (C) or Silver Blue (D), and immunodetection of PGM (C) or XI (D), respectively. Molecular weight (MW) markers were High Range Rainbow RPN76E (C) and Precision Plus Protein™ Dual Color Standards (BIO-RAD) (D). Photo documentation was made using ChemicDoc™ Imaging System BIO-RAD. The rectangles delimit areas that correspond to the gels and blots shown in Fig 3. (PDF) [file pone.0243867.s001.pdf]
