# Supplementary material for: Periplasm-enriched fractions from Xanthomonas citri subsp. citri type A and X. fuscans subsp. aurantifolii type B present distinct proteomic profiles under in vitro pathogenicity induction
Source: PLoS One. 2020 Dec 18;15(12):e0243867. doi: 10.1371/journal.pone.0243867 (PMC7748154; doi:10.1371/journal.pone.0243867)
Supplement: S1 Data — Matched peptides are in bold/underlined. In parenthesis is the number of different peptides with the same sequence. Proteins that had a score above the required minimum score for identity or extensive homology (p<0.05) are shown here. (PDF) [file pone.0243867.s002.pdf]

Data S1. XAC proteins identified by mass spectrometry (p< 0.05) in XAM-M medium based on the XAC306 database at NCBI and presented in Table 1. Matched peptides are in bold/underlined. In parenthesis is the number of different peptides with the same sequence. Proteins that had a score above the required minimum score for identity or extensive homology (p<0.05) are shown here.

Spot1

XAC0901. Conserved hypothetical protein

MANIAVVMVDGVADWEIGVVLPAAR**EWFGDQVAIASIDGQPLQSIGGLR**ITPEFALSDLA
PLEADLWILPGSER**WQAGEIPGLSGLLVER**VQQQRPVAAICGATLAFAYAGLLDERAHTS
NALAFLQEHVVPYAGAAHYRHEK**VVSADGVITAPGTSPVGFALACMR**QLHPER**TDTLAQL**
RGMFAGEFV

Matched Peptides: EWFGDQVAIASIDGQPLQSIGGLR,TDTLAQLR (2X), VVSADGVITAPGTSPVGFALACMR (2X), WQAGEIPGLSGLLVER (2X).

XAC0223. Conserved hypothetical protein

MKTSLLALGLLAALPFAASAAENLSYNFVEGDYVRTPTEGR**DADGWGVKASYAIAPNFHV**
FGDYSKQNADDNNNVFENTDSDFQQWGVGVGFNHEIATSTDFVARVAYR**KLDLDTPNINF**
DGYSVEAGLRNAFGEHFEVYALAGYEDFSKKR**GIDIGDNFYGR**LGAQVK**LNQNWGINGDI**
RMDGDGNKEWSVGPRFSW

Matched Peptides: DADGWGVKASYAIAPNFHVFGDYSK, KLDLDTPNINFDGYSVEAGLR, GIDIGDNFYGR LNQNWGINGDIRMDGDGNKEWSVGPR

Spot2

XAC0222. NAD(P)H–dependent glycerol-3-phosphate dehydrogenase

MSDLTHKIAVLGAGSWGTALAALLARHGHPTVLWGRDAAMVDTIDRTHENAR**YLPGIALP**
DSLRATTDLQAAVADATWILVVVPSHAFTETIR**LIAPLRPPGAGVAWATK**GFEPGSGRFL
HEVARDILGPSVPLAVVTGPSFAKEVTLGLPTAITVHGDDAAFAQVVADAMHGPTFRAYT
GDDMVGAELGGAMK**NVLAVATGVADGMQLGLNAR**AGLITRGLNEMLRLAAVIGARPETLM
GLAGLGDLVLTCTGDLSRNRRLGLALGR**GQSLDEAIREIGQVVESVQTADEVMRQAEHHG**
IELPISNAVRAVLHGEITPEAGLKELLARERKPEYPQTLFT

Matched Peptides: AVLHGEITPEAGLK, DILGPSVPLAVVTGPSFAK, EIGQVVESVQTADEVMR, FLHEVAR, GQSLDEAIR, LIAPLRPPGAGVAWATK, NVLAVATGVADGMQLGLNAR, QAEHHGIELPISNAVR, YLPGIALPDSLR (2X).

Spot3

XAC3579. Phosphoglucomutase / Phosphomannomutase

MPMTLPAFKAYDIRGRVPDELNEDLAR**RIGVALAAQLDQGPVVLGHDVRLASPALQEALSAGLR**ASGRDVIDIGLCGTEEVYFQTDYLKAAGGVMVTASHNPMDYNGMKLVR**EQARPISSDTGLFAIRDTVAADTAAPGEPTASEQSRTDKTAYLEHLLSYVDR**STLKPLKLVVNAGNGGAGLIVDLLAPHLPFEFVR**VFHEPDGNFPNGIPNPLLPENR**DATAKAVK**DNGADFGIAWDGDFDR**CFFFDHTGRFIEGYYLVGLLAQAILAKQPGGK**VVHDPRLTWNTVEQVEEAGGIPVLCKSGHAFIK**EKMRSENAVYGGEMSAHHYFREFAYADSGMIPWLLIAELVSQSGR**SLADLVEAR**MQK**FPCSGEINFK**VADAKASVAR**VMEHYASLSPELDYTDGISADFGQWR**FNLR**SSNTEPLLRLNVETR**GDAALLE**TRTQEISNLLRG**

Matched Peptides: DNGADFGIAWDGDFDR (7X), IGVALAAQLDQGPVVLGHDVR (5X),LASPALQEALSAGLR (5X), CFFFDHTGR, DTVAADTAAPGEPTASEQSR (3X), EQARPISSDTGLFAIR (2X), FPCSGEINFK (3X), GRVPDELNEDLAR (3X), LNVETR (2X), LTWNTVEQVEEAGGIPVLCK (4X), RIGVALAAQLDQGPVVLGHDVR

XAC1719. Enolase

MTTIAKILAR**EILDSR**GNPTLEAEVTLDDGSFGR**AAVPSGASTGTKEAVELR**DGDKTRYLGKGVRHAVDNVNGTIAETLK**NFDAADQQGLDR**R**LIDLDGTENK**GR**LGANALLGVSLAAAHAVAASR**KQPLWQYLSTITEADVALPVPMMNIINGGAHADNNVDFQEFMVLPVGCSSFSEALR**AGTEIFHSLK**SVLK**GHGLSTAVGDEGGFAPDFRSNVEALDTILEAIGK**AGYTAGEDILLGLDVASSEFYDNGK**YNLVGENK**RLTSEQFVDFLADWVAQYPIISIEDGLAEDDWAGWKLLTDRVGK**KVQLVGDDLFVTNPK**IFK**QGIDSGTANAILIK**VNQIGTLTETLEAIAMAHAANYASIVSHRSGETEDTTIADIAVATTATQIKTGSLCRSDRVAK**YNQLLRIEQALGSGAR**YAGR**DAFVSIK**R

Matched Peptides: AAVPSGASTGTK (2X), AGTEIFHSLK (2X), DAFVSIK, EAVELR, EILDSR, GHGLSTAVGDEGGFAPDFR, IEQALGSGAR, KVQLVGDDLFVTNPK, LIDLDGTENK, NFDAADQQGLDR, QGIDSGTANAILIK (2X), VQLVGDDLFVTNPK (4X), YNLVGENK, YNQLLR, LGANALLGVSLAAAHAVAASR, SNVEALDTILEAIGK (2X)

XACb0007. Lytic murein transglycosylase

MIMPSRLLRLTLGVSVCVAATSVAAQAIAPETTASSATDAGGQQQDPAPTSSFEQWLADF
RQRALAAGIGATTLDNALAGVTPDPAVHELDQRQPEFTQYLWDYLDARVTPSAIQEGQQL
LISQHALFEKLRQHYGVDPGILTAIWSMESGYGK**QIGDFYVIRSLATLAHEG**RRTTYGNT
QLLAALQILQTEKSIDRSQLVGSWAGAMGQTQFIPSTYRDYAVDEDGDQKRDVWNSKADA
LGSAANYLKQNNWTSAVPWGQEVQLSAGFDYAQADLTIKK**TVAEWQRLGVAP**RRPIAPAL
AQQLASVLLPTGYRGPAFLVFDNFRSILRYNNSTAYALAVGLLADGYAGRAGVKQPWPKD
DPPLNSTAQITELQQRLTDKGFDVGGIDGVLGAQTRQGIRAFQRSQQLPQDGYASTSLLA
RLRAP

Matched Peptides: QIGDFYVIRSLATLAHEG, TVAEWQRLGVAP (2X)

XAC0957. Elongation factor Tu

MAKAKFERTKPHVNVGTIGHVDHGKTTLTAALTKIGAERFGGEFKAYDAIDAAPEEKARG
ITISTAHVEYESPTRHYAHVDCPGHADYVKNMITGAAQMDGAILVCSAADGPMPQTREHI
LLSRQVGVPHIVVFLNK**ADMVDDAELLELVEMEVR**ELLSKYDFPGDDTPIIHGSARLALD
GDQSDIGVPAILKLVEALDSFIPEPTRDVDRPFLMPVEDVFSISGRGTVVTGRIERGIIK
VGDEIEIVGIRDTQKTTVTGVEMFRKLLDQGQAGDNAGLLLRGTKRDDVERGQVLCKPGS
IKPHTEFEAEVYVLSKDEGGRHTPFFKGYRPQFYFRTTDITGACQLPEGVEMVMPGDNVK
MVVTLINPVAMDEGLRFAIREGGRTVGAGVVAKIIK

Matched Peptides: ADMVDDAELLELVEMEVR (2X), VGDEIEIVGIR (2X)

XAC1776. Xylose isomerase

MYIGAKEYFPGIGKIGFEGRDSDNPLAFK**VYDANKTIGDKTMAEHL**RFAVAYWHSFCGNG
ADPFGPGTRAYPWDVGDTALNRAEAKADAAFEFFTKLGVPYYCFHDIDLSPDADDITEYE
SNLKHMVGVARQRQADTGIKLLWGTANLFSHPRYMNGASTNPDFNVVARAAVQVKAAIDA
TVALGGENYVFWGGREGYACLHNTQMKREQDNMARFLTLARDYGRSIGFKGNFLIEPKPM
EPMKHQYDFDSATVIGFLRQHGLDQDFKLNIEANHATLSGHSFEHDLQVASDAGLLGSID
ANRGNAQNGWDTDQFPTDLYDTVGAMLVVLRQGGLAPGGLNFDAKVRRESSDPQDLFLAH
IGGMDAFARGLEVANALLTASPLEQWRAERYASFDSGAGADFAAGK**TTLADLAKHAAGNA**
PQQISGRQEAYENLINQYLTR

Matched Peptides: TTLADLAKHAAGNA, PQQISG

XAC1158. Adenylosuccinate synthetase

MGQSVVVLGAQWGDEGKGK**IVDLLTEEIGAVVR**FQGGHNAGHTLVINGKKTVLHLIPSGI
LRDDALCLIGNGVVISPAALIKEISELEDAGVEVRSRLKISPAAPLIMPYHIALDQAREK
AAGGKAIGTTGRGIGPAYEDKVARRGIRIADLHYPPQLEELLRTALDYHNFVLTK**YLGVE**
AVDFQKTYDEALAFGEYVQPMKSDVAGILHDLRKQGKRVLFEGAQGALLDIDHGTYPYVT
SSNTTVGGALAGTGVGADAIDYVLGIAKAYATRVGGGPFPTELDDEVGQGIRDRGAEYGA
STGRPRRCGWMDIVALKRAVAINGISGLCITKLDVLDGMEKLKICIAYEYHGKRTEYAPL
DAQGWEECTPVYLEFPGWSENTHGITVWDDLPPAARAYLRALEELAGCPISIVSTGPDRD
HTMVLQDPFA

Matched Peptides: IVDLLTEEIGAVVR, YLGVEAVDFQK

XAC3236. Succinyl-CoA synthetase β subunit

MNFHEYQSKQLLAEYGIPVPAGKVAATPDEAVEVANSLGNGPWMVKAQIHAGGRGKAGGV
KFCKTTDDVKAAAAKMLGTKMSTYQTAGVELPINLVLVTTAGEIVKELYLSILVDRGTKT
ITYIASSEGGVEIEQVAAETPELIHALNVDFVEGVQGYHGRDFGFKLGLNAKQAGQFASI
MVNLYKLFNEKDLALVEINPLAILDDGNLYALDGK**FDSDDNAAFR**QKQLVAMRDKTQEDE
TEVTASELDINYVTMDGNIGCMVNGAGLAMATMDVIKLNGGEPANFLDVGGGANKQRVIE
AFKLILSSDKVEGIFVNIFGGIVRCDMIAEGIIAAVKEVGVKVPVVVR**LEGTNVEEGK**QL
LRDSGMAIIPADNINDGAKKVVEAVKNAA

Matched Peptides: FDSDDNAAFR, LEGTNVEEGK, MNFHEYQSK

XAC3463. TolC protein

MIRRSLVLALAAALSPMAAHATDLLQVYEMARNGDPQLAVAESTRLVNREGQVQARAALL
PQLDGSAGYTQSHRELEGVDGRSTTK**QRQYAIQGSQTIFNWAQFSNLRAQ**REVAKAADFT
LASANNDLITRTSAAYFQVLVGIESLAAAETNEAAAKKQFDYADKRLEVGLAPITDVHEA
RAQYDQARADTITARNTLK**DYYQALTELTGQPVVGL**RALPDEFRPEVPAAYSNVDQLVAT
AIADNPALKAQQLQVSAAEAQVSAARAGHLPTLSLGANVGRSNSWGGQGTVDEAAGNFTT
AGRNIDTDSVGITLTIPIFAGGATQSAVRQALSQRDIQQDTYEQQKRALDRNTRNAYQTV
VAGISEVEARRLAVVSAQAAYDASQVGLEVGTRTVLDVVQNQRTLFQAQLNYAQSRYNFL
QNRLLLGQAIGKLDITDLQDVNRLLSQDAESK**LQGSGSLQ**

Matched Peptides: QRQYAIQGSQTIFNWAQFSNLRAQ, LQGSGSLQ (2X), DYYQALTELTGQPVVGL

Spot4

XAC0680. 6-phosphogluconate dehydrogenase

MELGMVGLGRMGANMAERLVNGGHR**VHGYDPGANAR**TSAQAK**GIVTADALAALVSALPSP**
RVVWLMVPAGKIVDDTLAQLLPLLQAGDIVIDGGNSYYKDSQRRAALLQASGIAFVDCGT
SGGIWGLQEGYSLMVGGDEAAVTGLHPILATLAPAPDKGWGR**VGPSGAGHFTKMVHNGIE**
YGMMQAYAEGFALMQHKADFALDLHQVAEIWRDGSVVRSWLLDLTADALTHNPTMAGIAP
FVADSGEGRWTVAEAIDLEVSAPVITLSLMERLRSR**DKDSFTDK**LLAAMRNQFGGHAVMT
TTSAAPTIGSKDA

Matched Peptides: DKDSFTDK (2X), GIVTADALAALVSALPSPR (4X), MELGMVGLGR, MGANMAER (2X), MVHNGIEYGMMQAYAEGFALMQHK (3X), NQFGGHAVMTTTSAAPTIGSK, VGPSGAGHFTK (2X), VHGYDPGANAR, VVWLMVPAGK
