# Supplementary material for: Periplasm-enriched fractions from Xanthomonas citri subsp. citri type A and X. fuscans subsp. aurantifolii type B present distinct proteomic profiles under in vitro pathogenicity induction
Source: PLoS One. 2020 Dec 18;15(12):e0243867. doi: 10.1371/journal.pone.0243867 (PMC7748154; doi:10.1371/journal.pone.0243867)
Supplement: S2 Data — Matched peptides are in bold/underlined. In parenthesis is the number of different peptides with the same sequence. Proteins that had a score above the required minimum score for identity or extensive homology (p<0.05) are shown here. (PDF) [file pone.0243867.s003.pdf]

Data S2. XauB proteins identified by mass spectrometry (p< 0.05) in XAM-M medium based on the XauB database at NCBI and presented in Table 1. Matched peptides are in bold/underlined. In parenthesis is the number of different peptides with the same sequence. Proteins that had a score above the required minimum score for identity or extensive homology (p<0.05) are shown here.

Spot5

XAUB_08340. Conserved hypothetical protein

MSLKHFLNTQDWSR**AELDALLTQAALFK**RNKLGSELKGK**SIALVFFNPSMR**TRTSFELGAF
QLGGHAVVLQPGKDAWPIEFNLGTVMDGDTEEHIAEVARVLGR**YVDLIGVR**AFPKFVDWSK
DREDQVLKSFAKYSPVPVINMETITHPCQELAHALALQEHFGTQDLRGKKYVLTWTYHPKP
LNTAVANSALTIATRMGMDVTLLCPTPDYILDQRYMDWAAQNVAESGGSLQVSHDIDSAYA
GADVVYAKSWGALPFFGNWEPEKPIR**DQYQHFIVDER**KMALTNNGVFSHCLPLRRNVKATD
AVMDSPNCIAIDEAENRLHVQK**AIMAALVGQGSRD**S

Matched Peptides: AELDALLTQAALFK (4X), AIMAALVGQGSR (4X), DQYQHFIVDER (2X), DREDQVLK (4X), SIALVFFNPSMR (6X), YVDLIGVR (6X)

XAUB_14890. Polyphosphate-selective porin O

MRLNLLVLALAAAIVPAAANAATSIENWPTKYTFGDGTELGLTGNYAYDDNNFSGDDRLED
RNDFRRKEFGATIKKKGVYDAMVYYDFESKLWLDVFYRFETK**ALFGQDYGR**VRLGYMKVPV
GLEAVQSSRAGSFMELGLPIQAVFQGRRTGAEWTLERQQYLLQAGAYGGKDLQGDNPGTTQ
AVHAAWTPSKAEGDVLHLGIAGSIENPRGYSDGRGVSFSPRVRLRARPEAGLTDVR**LIDTG**
TILDVDHVIRTGLEAVWIHGPFSLQSEALRAEVARNANQPHFIAQGQYVYGTWTLTGESRS
YAGGVPGNIKPSHDYGAVELTARYSRLNLEDNNVHGGRQHDTTIGANWYLTSHFKFQANYS
WVDSSRNGVHETPHVMELRAQVQF

Matched Peptides: ALFGQDYGR (2X), LIDTGTILDVDHVIR (4X)

Spot6

XAUB_32290. Adenylate kinase

MR**LVLLGPPGSGK**GTQATR**LKDTFDIPHISTGDLLRAEVAAGSPLGLK**AKEVMAR**GDLVSD DILLGMLEARLGQADVAKGFILDGYPRNVAQANALDELLGK**IGQPLDAVVQLDVASELLVE RIAGRAK**AEGREDDNPESVR**KR**LQVYTDSTAPVIGFYEQR**GKLARVDGVGSLDEVLERISK ALGR

Matched Peptides: AEGREDDNPESVR (2X), AEVAAGSPLGLK (6X), EDDNPESVR (2X), GDLVSDDILLGMLEAR (4X), GFILDGYPR (6X), LGQADVAK (4X), LKDTFDIPHISTGDLLR (4X), LQVYTDSTAPVIGFYEQR (2X), LVLLGPPGSGK (8X), NVAQANALDELLGK (2X), NVAQANALDELLGK (6X)

XAUB_24740. Poly hydroxyalcanoate granule associated protein

MTTGYDQNGNRGNAAQGFQAQAEQISRRLGESAQTVWLAGLGALGRVQNEGSKLFDSLVRE GAAYERTGQRRAAESVDELREEVETQFEQARDTAVRGWDKLGKAFDERVK**GVLRTLNIPEQ EELENL**RREVESLKAQVRANTAATKRANRTANQAAQAASETTGGGAGTGAASSSGTPGAFD PE

Matched Peptides: GVLRTLNIPEQEELENL (2X)

XAUB_29250. DNA-binding related protein

MSKKKNANKPVVVSDALPVAAASAPHIDIGIKDSDRKQISDGLARYMADAFTLYLKTHNFH WNVTGSMFNSLHTMFETQYTEQWAALDEVAERIRALGYNAPGSYREFVALTSIPEEPGLSD SADWREMVRQLVSGNEAVCRTARK**VLGTADDAGDDPTVDLLTQ**RLQTHEKYAWMLRSLLQ

Matched Peptides: VLGTADDAGDDPTVDLLTQ

Spot7

XAUB_07390. Carbamoyl-phosphate synthase large subunit

MPKRTDLKTILIIGAGPIVIGQACEFDYSGAQACKALRDEGYRVVLVNSNPATIMTDPNMA DAVYIEPINWQTVEKIIAKEKPDALLPTMGGQTALNCALDLADHGVLEK**YNVELIGAK**REA IRMAEDRELFRVAMGEIGLDCPTAAVAHTLEEALEIQTRVGYPTIIRPSFTLGGSGGGIAY NREELIEIVGRGLELSPTTEVLVEESVLGWK**EFEMEVVR**DTADNCIIVCAIENLDPMGVHT GDSITVAPAQTLTDKEYQRLRDASIAVLRKIGVDTGGSNVQFGISPTTGRVVVIEMNPRVS RSSALASKATGFPIAKVAAKLAVGYTLDELKNEITGGLTPASFEPSIDYVVTKIPRFAFEK FPQADARLTTQMKSVGEVMAMGR**TFQESLQK**ALRGLETGKIGLDPTGLDLSSEDDIATLKR

ELKAPGPER**LFYVADAFR**AGMTVADVYALSFIDPWFLDQIEEIISHEQQLADDGMAALDAP RLRMLKRAGFSDARMAQLIGSNEESVRTLRRALK**LRPVYK**RVDSCAAEFGTSTAYLYSTYE DECEALPTDRDK**IMILGGGPNR**IGQGIEFDYCCVHAALALRDDGFETIMVNCNPETVSTDY DTSDRLYFEPLTLEDVLEIVELEKPKGVIVQYGGQTPLKLARALEANGVPVIGTSPDSIDL AEDRERFQQLVDKLGLKQPPNRIAR**NAEEALVLAR**EIGYPLVVRPSYVLGGRAMEIVYGES DLARYVRDAVK**VSNDSPVLLDR**FLDNAVEVDVDIIADKDGNVLIGGVMEHIEEAGVHSGDS SCSLPPYSLSPQTQAELRRQVVMLAEGLNVVGLMNTQFAVQVNEAGDDIVYLLEVNPRASR TVPFVSK**AIGMPLAK**IAARCMAGK**TLAEQGATKEIVPDYYSVKEAIFPFAKFQGVDPILGP EMRSTGEVMGVGRSFSAAFAR**AQEAGGIKAPPLGKAFVSVRDPDKQR**VLPVAQALVER**GFT LVATRGTGAWLQQNGLSCEIVNKVAEGRPHIVDSIKNGEIVYIVNTTEGR**AAISDSFSIR**R EALQHR**VTYSTTVAGAK**ALVQSLEFRGTGPVWSLQELHKELEA

Matched Peptide: YNVELIGAK, EFEMEVVR, TFQESLQK, LFYVADAFR, LRPVYK, IMILGGGPNR, NAEEALVLAR, VSNDSPVLLDR, AIGMPLAK, TLAEQGATKEIVPDYYSVKEAIFPFAKFQGVDPILGPEMR, STGEVMGVGRSFSAAFAR, VLPVAQALVER

Spot8

XAUB_40430. 50S ribosomal protein L3

MTKK**YSLGFVGR**KAGMSRVFTEDGR**SVPVTLIEATPNR**IAQIKTVEVDGYSAVQVTVGARR AALVNKPAAGHFAKAKVEAGR**GLWEFRVEDAQLGDFAVGGEIKADIFEVGQK**VDVQGVTKG KGFQGTIKRYNFR**MGDATHGNSLSHR**APGSLGQRQTPGRVFPGKKMSGHMGAVQQSTQNLE VVKVDVERGLIAIR**GAVPGAAGGDVIVRPASK**A

Matched Peptide: YSLGFVGR, SVPVTLIEATPNR, GLWEFRVEDAQLGDFAVGGEIKADIFEVGQK, MGDATHGNSLSHR, GAVPGAAGGDVIVRPASK

XAUB_08900. NonF-related protein

MKVLMVLTSHDQLGDTGKKTGFWLEEFAAPYYVFK**DAGADITLVSPKGGQPPLDPK**SDEPD AQTDATKRFKQDSEAQQALASTHRLADVKADDFDVLFYPGGHGPLWDLAEDADSIALIEAF AKADKPLGLVCHAPGALRRVKGSDGKPLVNGR**RVTGFTNREEEGVGLAK**IVPFLVEDVLTE LGGNYEKGADWGVYVVTDGTLVTGQNPASSEK**AAETLLAMAK**R

Matched Peptide: DAGADITLVSPKGGQPPLDPK, RVTGFTNREEEGVGLAK, KAAETLLAMAK

XAUB_05770. Elongation factor P

M**ATVGMNDVK**NGMKILVNNEPAVITETEYVKPGKGQAFTRMKYRFIKSGRVVEMTMKATDD
VEVADVVDTDMRYLYSDGEYWHFMDPETFEQVQTDKAGMGGADKWLKGEEDCIVTLWNGTP
IWVQPPNFVELK**ITETDPGVR**GDTSGGGGKPATLETGAVVR**VPLFVNQDEIIK**VDTRSGEY
SARVK

Matched Peptide: ATVGMNDVK, ITETDPGVR (2X), VPLFVNQDEIIK

XAUB_27940. Two component system regulatory protein

MR**ILLVEDEAPLR**ETLAARLKREGFAVDAAQDGEEGLYMGREVPFDVGIIDLGLPKMSGME
LIKALRDEGKKFPVLILTARSSWQDKVEGLKQGADDYLVKPFHVEELLARVNALLRRAAGW
SKPTLECGPVALDLAAQTVSVNGANVDLTSYEYKVLEYLMMHAGELVSKADLTEHIYQQDF
DRDSNVLEVFIGRLRKKLDPDGELKPIETVRGRGYRFAIPRTEG

Matched Peptide: ILLVEDEAPLR

Spot9

XAUB_33110. Anthranilate synthase component I

MITAEQFQRQAAEGHTRIPVVR**EVLSDLDTPLSVYLK**LADGAYTYLFESVEGGERFGR**YSI
IGLPAR**RVYSFRAHTLEVSEHGEVVETR**EVADPLAEVDALR**AEHSVPQLEGLPGFTGGLVG
WFGFECIQYIEPRLGSGDKPDELGTPDILLMLSEELAVFDNLKGR**LYLIVHADPR**QPQAYV
RANRRLDELAHRLRQGGAGYPQAQISDAIDEADFHSSFTREEYHAVVRKAQEYVRAGDIFQ
VVPSQRLRVPFRARPVDVYRALRALNPSPYMYFLDVGGTQVVGSSPEILARLRDGV

VTVRPIAGTRPRGATPELDK**ALEAELLADPK**ER**AEHVMLIDLGR**NDVGR**VAEPGTVKVGEQ
FVIERYSHVMHIVSEVTGTLKAGLNYSDVLRATFPAGTVSGAPK**IR**ALEIIRELEPVKR**NV
YSGAVGYIGWHGDADTAIAIRTAVIQDGYLYVQAGGGVVYDSDPDLEWQETINKGRALFRA
VAQAAKGL

Matched Peptide: EVLSDLDTPLSVYLK, VAEPGTVKVGEQFVIER, YSHVMHIVSEVTGTLKAGLNYSDVLR, ATFPAGTVSGAPK, ALEIIRELEPVKR, YSIIGLPAR, EVADPLAEVDALR, LYLIVHADPR, ALEAELLADPK, AEHVMLIDLGR

XAUB_05850. GDP-mannose pyrophosphorylase

MSDVLPIILSGGSGTRLWPLSRESYPK**QFLPLVGDKSMLQSTWL**RAAPVAGHAPIVVANEE HRFMAAEQLQQLGVK**PSAILLEPKGRNTAPAIAVAALEAT**RDGADPLLLVLPSDHVIGNEA AFQAAVKVAAEAAAQGKLVTFGIKPTAPETGYGYIKAGAGTAASAVERFVEK**PDLATAQSY LASGEYYWNSGMFLF**RASRYLEELRKFHPAIADACQKAWENGKRDADFTRLDK**DAFAASPS DSIDYAVME**KTADAVVVPLDAGWNDVGSWSSLLDVSNQDAQGNAHHGDVIQLDCQNTYAYG SRLIAMVGLEDVVVVETPDAVLVGHRDRIQEVK**DVVSQIKTAG**RSEATWHRKVYRPWGAYD SIDMGQRHQVKRITVKPGAVLSLQMHHHRAEHWIVVSGTAEVTRGEEVLLLTENQSTYIPL GVTHRLRNPGK**LPLELIEVQSGSYLGEDDIVRFEDTYGRA**

Matched Peptide: QFLPLVGDKSMLQSTWL (2X), LPLELIEVQSGSYLGEDDIVRFEDTYGRA, DAFAASPSDSIDYAVME (2X), PDLATAQSYLASGEYYWNSGMFLF, PSAILLEPKGRNTAPAIAVAALEAT, DVVSQIKTAG (2X)

XAUB_39520. S-adenosyl-L-homocysteine hydrolase

MNAVTKITPHTDYK**IADISLADWGR**KELDIAEHEMPGLMSIRRK**HAQTKPLKDVR**ITGSLH MTIQTAVLIETLKDIGADVRWASCNIFSTQDHAAAAIAATGTPVFAWKGETLEEYWDCTLD ALTFTLPDGTLTGPELVVDDGGDVTLLIHKGYELENGSTWVDEPASSHEEGVIKALLKRVA VERPGYWGRVVKDWKGVSEETTTGVHRLYQIAEAGK**LLIPAINVNDSVTK**SKFDNLYGCR**E SLADGLKRAMDVMLAGK**VAVVCGYGDVGKGSAASLRAYGARVIVTEIDPICALQAS MEGFEVNTIESTLGRADIYVTTTGNKDIITVEHLQAMKDQAIVCNIGHFDNEIQVDALKAL KDVQKINIKPQVDKYVFPNGNAIFLLADGRLVNLGCATGHPSFVMSNSFANQTLAQIDLWE KRDTYEKK**VYILPKHLDEEVAR**LHLEKIGVKLTTLTKDQADYLGVDVAGPYKPDHYRY

Matched Peptide: IADISLADWGR, HAQTKPLKDVR, LLIPAINVNDSVTK, ESLADGLKRAMDVMLAGK, VYILPKHLDEEVAR

Spot10

XAUB_41080. Isocitrate dehydrogenase

MTQTITVIRGDGIGPEIMDATLFVLDALQAGLTYEYADAGLVALEK**HGDLLPESTLASITK** NKVALK**SPLTTPVGEGFSSINVAMR**RKFDLYANVRPAKSFPNTKSR**FADGVDLITVR**ENTE GAYLSEGQTVSEDGETAFSGTRITRKGSERIVRYAFELAKSTGRKKVTAVHKANIIKSTSG LFLKVARDVAAQYPEIEFQEMIVDNTCMQLVMRPEQFDIIVTTNLFGDIISDLCAGLVGGL GLAPGANIGLDAAIFEAVHGSAPDIAGQGKANPCALLLGAAQMLDHIGQPQNAERLR**EAIV ATLEAKDSLTPDLGGTGNTMGFAK**AIASRL

Matched peptide: DSLTPDLGGTGNTMGFAK (4X), EAIVATLEAK (2X), FADGVDLITVR (4X), HGDLLPESTLASITK (6X), SPLTTPVGEGFSSINVAMR (6X)

XAUB_40770. Outer membrane protein

MNKKILTAALLGGLAVAQAASAQEFDDRWYLTGSAGFNFQDSDRLTNDAPFVTLGLGKFVS
PNWSIDGELNYQNPNFDANQDLNWSQYGISFDLRRHFIQEGRGWNPYLLFGLGYQR**SEEEF
DATPNPVSPGQQK**DGNFAAKAGVGLQTTFDKRVAVRAELAYRADFNDQSVAAPQEDWFGDV
LASVGVVIPLGPAPSTAPPPAPAPVAPSCADLDDDGDGVNNCDDKCPNSQPGQTIGPDGCP
VPVSIDLKGVNFDFNKSTLRPDAVSILSEATEILKRYPDLK**VEVAGHTDSK**GTDAYNQKLS
ERR**ATTVYDYLTK**NGVDASRL**VGPIGYGESRPIAPNTNPDGSDNPEGR**AKNRRTELNVQN

Matched Peptide: ATTVYDYLTK (2X), VGPIGYGESRPIAPNTNPDGSDNPEGR (2X), SEEEFDATPNPVSPGQQK (2X), VEVAGHTDSK (4X)

XAUB_20300. Glyceraldehyde-3-phosphate dehydrogenase

MAIKVGINGFGRIGRNVLRSAVQNFANDIEIVAINDLLEPDYLAYMLQYDSVHGRFKADVS
VDGNTLIVNGKKIRLTQERDPANLKWDAVGADVVIESTGLFLTKETAQK**HIDAGAK**KVILS
APSKDDTPMFVYGVNDKTYKGEAIISNASCTTNCLAPLAKVINDKWGIKRGLMTTVHAATA
TQKTVDGPSNKDWRGGR**GILENIIPSSTGAAK**AVGVVIPELNKKLTGMSFRVPTSDVSVVD
LTVELEKPATYAEICAEVK**AQSEGALK**GVLGYTEDKVVATDFRGETCTSVFDADAGIALDS
TFVKLVSWYDNEWGYSNKCLEMVRVVAK

Matched Peptide: AQSEGALK (4X), GILENIIPSSTGAAK (4X), HIDAGAK (2X)

Spot11

XAUB_12290. TonB-dependent outer membrane receptor precursor

MSNQFRRQVLKRTALAVVLGACLTNGAVYAQSTTGSIYGSAPSEAGSTIVVQSDTGLSRTI
TVDANGRYNLGSLPVGAYTVTLKRGDQVVDTRKNVQLRVGSGTEVSFAGAAASGGNADATT
LGAITVTAANAPK**IDVSSTSARSVITSEQLATLPLGR**SAEAIALLAPGAVSGAGAFNNGSR
SVVSFGGSGVTENAYYINGFNVSNPLSNLGGVSLPYGAIDQQETYTGGYSAKYGRSTGGVI
NQLGKRGTNEWHFGVQTVWEPDSLASSRGDVWFPNATLPAGYKYDTPDQPGTLYRAGKDNK
QTR**TVYSAYAGGPLIEDR**LFIFVAGESEKVDGVSTNASSDSIQAR**NNYEYSTPK**FYGKLDW

NINDSNIVEYTRIQNTDRR**SGYYTSFDYDGLVGGDRTGTYPDTYK**IKDTYDVFKYTGYITD
DLTLNATWGRSTQHNQQFNPFISDLPFLGSVTSQNPAITGGTPIRNNQATNR**AKADDPINK**
SRSLRLELNYR**LGDHDLTAGVDNMYFNAYDEGVR**TTGPGYQWIYGRAADEQTAVRPGLGVG
VTGPGSNGYYAQQRIFTTTTSMAVEQKAYYLEDRWQVNDRWLLTLGIRNDQFTNYNSDHVE
YVDSGDQWAPR**FGASWDVFGDSSLK**VFANLGR**YYLALPNSVAIR**GASASTYTDEYFTYTGI
DANGEPTGLTPIGPGPVSSNGEYGQAPDPNAFAPTDLK**SQYQDEFILGFEKTLGESWNSGA**
KFTYRKLQSAIDDVCDTAKIADKLTASGIDADAVEIPGCVMFNPGKTNTYDLANADGSGYT
QVRMSQQDWGFTDKAKRSYVSVDLFLEHPFNEKWYGRVDYTWSHSYGNTEGQVKSDLGQAD
VSKTQDWDAAALMYYAGGSLANDRRHQLKAFGAYQISPEWMASATLRVMSGTPRTCLGYFE
DGLDPISYGSAYHYCGGQPSRPGDAGRTPWIKNLDLGVTYRPSFADHKLAVGLQVFNVLND
RSANRVDGVYETDPGLVSNTYGIGLQDYSYNTPRYLRLSASYDF

Matched Peptide : AKADDPINK (2X), FGASWDVFGDSSLK (2X), IDVSSTSAR (2X), LGDHDLTAGVDNMYFNAYDEGVR (2X), NNYEYSTPK (2X), SGYYTSFDYDGLVGGDR (4X), SQYQDEFILGFEK (4X), SVITSEQLATLPLGR (2X), TGTYPDTYK (2X), TLGESWNSGAK (2X), TVYSAYAGGPLIEDR (4X), YYLALPNSVAIR (4X)

XAUB_17620. TonB-dependent receptor

MSTPLAAQTAPAPQSASTVPGSSQADPATLDTVQVSGIR**GSLTSSMNVK**RDAQGIVDGIVAEDI
GKFPDTNLAESLQR**ISGVSIDR**SLGEGSRVTVR**GVGPDFNLVLLNGRQMPGASIEESNASNSR**A
FDFANLASESISGIEVFKTSRASTPTGGIGATINIK**TARPLDNPGMHANVGLKGVHDSSNENLP**
GRLQGDSLTPEISGIFSNTSADGRFGVSLSGSYQER**DFGYSQVGVPNGWR**AFRGDSTAYGTIPQ
PGAPGSENIVNRPGPNDIYSVPQNLNYRVVGVERQRTNGQLTLQYKPLDNITTTLDYTYSENKI
QQQRNEMSVWFNYGPSASAWTK**GPVAGPITYSEIVNPPTSDLATAGSQAATR**NQNKSLGFNVDW
AVNDQFK**LNFDIHRSTAEAGADSPYGSSNSLGVSGFYRGTSVVDFSK**DFPVLQQQLGFGLNGLD
PSR**TLVTGSAFR**NSYMKSEIDQAQVNGDFTFENYSQLK**FGIGSTEVK**NR**SAFSNVQR**DTWGGNG
TAADYADDLWIPSSFAQYFDAIDGSGNPAQFNQLFLFDFERVR**QAAAQAAGDESLYR**ISPVFTT
DRRVTEKSKNAYLQWGNSWDDLR**VPISLAAGVR**YEETKVQAQALVPVAVGIDWVANNELPIRLA
DSAFSGGSGKYEYWLPSLDLSFKLREDLVLR**GSYGETIGRPGWGDIQGGQTLNQIGR**IEGGSGQ
EGNPGLKPLLSHNIDLSLEWYYGEASYASVGFFRKNIDNYVGVTTRNDTSLGLHTPVGGAYWNQ
ALANGCATADLTCIRNYIFR**NFAGQPGVARGTDDTNGNATGTISGQPGDPVANFSITAPANQR**S
ASLDGWEFNVQHMFGQSGFGVSANYTKVDSGLTYNNYVIGEQFALEGLSDSANLVGFYEKGQWQ
VR**AAYNWRDEFLAAR**FDGSGLPNPVYTEAYGQLDLNIGYKWTDNLSLSLEAINLTNEIQRQHGR
QKNEIIYATQTGPRYMLGLRYKFW

Matched peptide: FGIGSTEVK (2X), GPVAGPITYSEIVNPPTSDLATAGSQAATR (4X), GSYGETIGRPGWGDIQGGQTLNQIGR (2X), GTSVVDFSK (2X), GVGPDFNLVLLNGR (4X), ISGVSIDR (2X), LQGDSLTPEISGIFSNTSADGR (2X), NFAGQPGVAR (2X), QAAAQAAGDESLYR (2X), STAEAGADSPYGSSNSLGVSGFYR (2X)

XAUB_15610. TonB-dependent outer membrane receptor

MRHHHPHSARPARKLLSCALASCLLLGAAPAFAQSTAATIRGQVTVDAAPAAQAQVTATNLATG
LTRTVQVSNGGYSVGGLPPGSYRIDVTANGQTSSQNVTVQVGQTATLNLGVGGEPATAAGGNAT
TLDAVQVKAPPVLVETRTSENATYISNVQIQNLPRATRNFLELADTVPNVQFTREANGTTKVRT
GATSAEGTNVYIDGVSQKNYVLTGGVSGQDSSRGNPFPQSAIGEYK**VITSNYK**AEFDQVSGAAI
VASSKSGTNDFHGSFFWDTSNDSWREESPLEKKAGVRDDFEETQYGATFSGPILKDKAHFFIAY
EAKEYTTPNVVIPGSIYSDRVDQLPAQLQPLVVTSSTPFKEDLYFGKIDWTIGENNLFELTGKY
RKEDELNDVGRTSTYEHGSINGQEEKRANLRWQYSGANFLNEANLSYESAFWNQAPINNGNGYI
LSYAPVRGNETDILAAGAGSSFQRKGQKGWTFQDDLTLNSLEWHGAHTVKMGVKFKSIDLDSTQ
FNPANPQYYYNILTDVETPYRVR**FGAPLVEGGGSVVSK**NKQYGIYLQDDWEVNEHWTLNLGVRY
DYEETPAFLDFVTPSDVASALQNWPNLRNANYNINDFISTGNNRKAFKNAWQPRLGASYDLFGD
QAHVIYGGAGRAYDRNIFDYLALEQLNNSFKSYSYYFTSANNPTCLGDPCTAWNPAYNSQDGLN
TLTANSTGGGGREVYLIDNNLKTPYSDQFSIGMRNMVPLWGQDWFTDVTLSRIESHDGFAFVRG
NRLPDGSFFRPGTTSGVPTDGPNGYSAIVLGTNGVETRNNQLALQVEKPFDEESGWGLTVAYTY
SDAKENRQFGEHYALDRERIQDYGWREAGGIPKNR**LVVTGIYELPYEIK**LSGKLSLASQTARYG
QNCLAGNDQCEIIQFKPDGTLGYKEFSIAANKEWDTGGGVKFNVRADILNVFNWVNYATFNGDT
GTLQDLNTAYGTPTGVLASPMRTFRLAFGLNW

Matched Peptide: VITSNYK (2X), FGAPLVEGGGSVVSK, LVVTGIYELPYEIK

Spot12

XAUB_26290 Secreted protein

MWSLAPHIALLPPLTGIRMPLLARSVSALLPLMFATACSAAAPAK**SGTPDAPVAACTAKVR**
PGQDLQKAIDKLPQSDKPTVLCLEKGEFPLNGLVSIHRGNFTLRGQGPSTIVR**MADGVQQP**
VLVVGDYENQQPTGVIRNVSIEDMQIVASTGDK**EFMPERPYLSNSAVVVR**SGQGIR**LAGLQ**
VNKCR**SACLLSEYDAREITIENNDVSGAIWDGVSFNR**TAKVTMVNNYIHDNVAAGLTTEHL
EDSEILNNRFERNGSQGIYLSDARRNRFSNNQFDGNK**VAGVFLACAIR**YRTPEILCWDNSM

SQDNIFENNR**FANTPFTYTIGVDRAANCTAADFKPNLWRNNQADMAGVDIDPQR**YGYCVRH
EQ

Matched Peptide: AANCTAADFKPNLWR (4X), AIDKLPQSDKPTVLCLEK (2X), EFMPERPYLSNSAVVVR (2X), EITIENNDVSGAIWDGVSFNR (4X), FANTPFTYTIGVDR (6X), LAGLQVNK (2X), MADGVQQPVLVVGDYENQQPTGVIR (4X), NNQADMAGVDIDPQR (2X), SACLLSEYDAR (8X), SGTPDAPVAACTAK (2X), VAGVFLACAIR (4X), VRPGQDLQK (4X)

Spot13

XAUB_15870. Conserved hypothetical protein

MTSTHRAAAFTNPHSRIPTPMKSICVYCGSNAGNKPAYVERAIALGDRIAKQGLRLVYGGG
NVGLMGTVANAVLAAGGEVTGVIPQQLADWEVAHR**GLTTLEIVGSMHER**KMRMFELSDAFV
ALPGGFGTMEEIFEMLTWRQLGIGNKPCAFLDIEGFYAPLIGMIDRMVEERFLHPDQRADL
WYGADIEQMLEWMR**HYTPAQASK**WIDEKRRSTLV

Matched Peptide: GLTTLEIVGSMHER (4X), HYTPAQASK (7X)

Spot14

XAUB_28440. Conserved hypothetical protein

M**SNLDKQER**KEEARDLNRDPISGAPGSHPVGVGVGGIAGGAAAGALAGTVFGPLGTLIGAA
AGVVAGAAAGKGVAER**LDPTVETEYWR**QEHRNRPYYK**EGTDYDRDYATVYGFGLQAR**ETRP
TSTWEETEATLAGEWPRNRGQSRLEWDEARLAARDAWERADR**THTVYRDSDTHYAGR**FDSA
SYRDADYSYDDYRPAYRYGMQARQQHAGRQWDDHLER**DLGDGWDR**FKANSRLSWEKAKHAV
REAFDSEHHDAAAR**HPTDPR**V

Matched Peptide: DLGDGWDR, DSDTHYAGR (3X), EGTDYDRDYATVYGFGLQAR, HPTDPR, LDPTVETEYWR, SNLDKQER, THTVYR

Spot15

XAUB_41570. Carboxyphosphonoenolpyruvate phosphonomutase

MTFSSTPSAGARFRAALAAESPLQVIGAINANHALLAQRAGYQAIYLSGGGVAAGSLGLPD
LGINTLEDVLIDVRRITDVCELPLLVDVDTGFGPSAFNIERTIKSLIKAGAGGCHIEDQVG

AKRCGHRPGKEIVSQGEMVDRVKAAADAK**TDAAFFLIAR**TDAIQMEGVEAAIERAIACVEA
GADGIFAEAAYDLDTYRRFVDAVR**VPVLANITEFGK**TPLLTRDQLAQAGVAIQLFPLSAFR
AANKAAEAVYTAIRRDGHQQGVLDSMQTREELYERIGYHEYEQR**LDTLFAR**KGA

Matched Peptide: LDTLFAR, TDAAFFLIAR, VPVLANITEFGK (3X)

XAUB_08740. UTP-glucose-1-phosphate uridylyltransferase

MSQRIR**KAVFPVAGLGTR**FLPATKTVPKEMLPIIDKPLIQYAVDEAIQAGCDTLIFVTNRY
KHSIADYFDKAYELEQKLER**AGKLEQLELVR**HALPEGVR**AIFVTQAEALGLGHAVLCAK**AV
VGNEPFAVLLPDDLMWNR**GDAALTQMANVAEASGGSVIAVEDVPHDKTASYGIVSTDAFDG**
RKGRINAIVEKPKPEVAPSNLAVVGRYVLSPKIFDLLEATGAGAGGEIQLTDAIAELLKEE
QVDAFRFEGRRFDCGAHIGLIEATVHFALEHEKHGGPAKEILREALAQADARG

Matched Peptide: AGKLEQLELVR, AIFVTQAEALGLGHAVLCAK, AVFPVAGLGTR, GDAALTQMANVAEASGGSVIAVEDVPHDK, KAVFPVAGLGTR (2X), LEQLELVR, TASYGIVSTDAFDGR (2X)

Spot16

XAUB_08740. UTP-glucose-1-phosphate uridylyltransferase

MSQRIR**KAVFPVAGLGTR**FLPATKTVPKEMLPIIDKPLIQYAVDEAIQAGCDTLIFVTNRY
KHSIADYFDKAYELEQKLER**AGKLEQLELVR**HALPEGVR**AIFVTQAEALGLGHAVLCAK**AV
VGNEPFAVLLPDDLMWNR**GDAALTQMANVAEASGGSVIAVEDVPHDKTASYGIVSTDAFDG**
RKGRINAIVEKPKPEVAPSNLAVVGRYVLSPKIFDLLEATGAGAGGEIQLTDAIAELLKEE
QVDAFRFEGRRFDCGAHIGLIEATVHFALEHEKHGGPAKEILREALAQADARG

Matched Peptide: AGKLEQLELVR, AIFVTQAEALGLGHAVLCAK, AVFPVAGLGTR, GDAALTQMANVAEASGGSVIAVEDVPHDK, KAVFPVAGLGTR (2X), LEQLELVR, TASYGIVSTDAFDGR (2X)

XAUB_09130. Secreted protein

MKTTHKLLLPLALTLAIAACSKPAENTAAPAAETPAAAATPADAAAAPAPAPAAAASNAPA
VEVASGTYTLDPSHTDVLAQWSHFGFSNPSAHFGNVDGTLVYDAADVTK**STVQVTLPLSGL**
NSFTAKFDEHLK**SGDFFDAAK**FPTATFKSTK**VEAAGANK**LTVTGDLTIKGQTKPVVLDVTL
NGAGEHPMKKVPAAGFDATTTIKRSDFGLGQYAPNVSDEVKIRITTEALQAKAGDAAAKDA
AAK

Matched Peptide: SGDFFDAAK, STVQVTLPLSGLNSFTAK, VEAAGANK

XAUB_41570. Carboxyphosphonoenolpyruvate phosphonomutase

MTFSSTPSAGARFRAALAAESPLQVIGAINANHALLAQRAGYQAIYLSGGGVAAGSLGLPD
LGINTLEDVLIDVRRITDVCELPLLVDVDTGFGPSAFNIERTIKSLIKAGAGGCHIEDQVG
AKRCGHRPGKEIVSQGEMVDRVKAAADAK**TDAAFFLIAR**TDAIQMEGVEAAIERAIACVEA
GADGIFAEAAYDLDTYRRFVDAVR**VPVLANITEFGK**TPLLTRDQLAQAGVAIQLFPLSAFR
AANKAAEAVYTAIRRDGHQQGVLDSMQTREELYERIGYHEYEQR**LDTLFAR**KGA

Matched Peptide: LDTLFAR, TDAAFFLIAR, VPVLANITEFGK (3X)

Spot17

XAUB_40240. 50S ribosomal protein L25

MAKTHEIKVERRADEGKGASRRLRHAGVIPAIVYGGELEPVSIQLNHEQIWLAQQNEWFYS
SILDLNLNGGVQQVLLRDMQRHPFK**QLIMHIDFQR**VSANEKLSAAVPLHFINEASSPAGK**S**
SEVVVTHELNEVQVVCLPKDLPEFIEVDLGALEVGNVIHLSEIKLPAGVEIPELKLGK**EHD**
VAVVAAKHVR**IEEDDAAGEEGSEGAETK**

Matched peptide: EHDVAVVAAK, IEEDDAAGEEGSEGAETK (2X), LPAGVEIPELK, LSAAVPLHFINEASSPAGK (4X), QLIMHIDFQR (2X), SSEVVVTHELNEVQVVCLPK

XAUB_17400. Conserved hypothetical protein

MSRRVPLPALLPLLLITPSVWAQAVAPAPADPAAVVLPSPWSGSSGELGYAAAHGNSTTDS
LNGRVRLR**YTDGDWIHSLDATALRSSSEYTNTNDDGSTTR**ERQTTAER**YTGSVGSALQLGE**
HRQLTATGRYEHDDFATYDR**LATFGIGYGTRLIDADRFYLDAQVGPGVR**R**AHNSDEDRNET**
GLIGRGLFDLKYTVTDNTDLINTLLVESGEYNTYAQNDFGVQVSMNSHFALKAAWQMRHNS
DVSDGDKK**TDTLTTVNLVYTFK**

Matched peptide: AHNSDEDRNETGLIGR, FYLDAQVGPGVR, LATFGIGYGTR (3X), LIDADR, LIDADRFYLDAQVGPGVR, QLTATGR (2X), SSSEYTNTNDDGSTTR, TDTLTTVNLVYTFK (6X), YTDGDWIHSLDATALR, YTGSVGSALQLGEHR (4X)

Spot18

XAUB_17400. Conserved hypothetical protein

MSRRVPLPALLPLLLITPSVWAQAVAPAPADPAAVVLPSPWSGSSGELGYAAAHGNSTTDS LNGRVRLR**YTDGDWIHSLDATALRSSSEYTNTNDDGSTTR**ERQTTAER**YTGSVGSALQLGEHRQLTATGR**YEHDDFATYDR**LATFGIGYGTRLIDADRFYLDAQVGPGVR**R**AHNSDEDRNETGLIGR**GLFDLKYTVTDNTDLINTLLVESGEYNTYAQNDFGVQVSMNSHFALKAAWQMRHNS DVSDGDKK**TDTLTTVNLVYTFK**

Matched peptide: AHNSDEDRNETGLIGR, FYLDAQVGPGVR, LATFGIGYGTR (3X), LIDADR, LIDADRFYLDAQVGPGVR, QLTATGR (2X), SSSEYTNTNDDGSTTR, TDTLTTVNLVYTFK (6X), YTDGDWIHSLDATALR, YTGSVGSALQLGEHR (4X)

Spot19

XAUB_08010. ATP synthase subunit-β

MSQGKIVQIIGAVVDVEFPRNEVPKVYRALKVDGTEITLEVQQQLGDGVVRTIALGSTDGL KRNLVATNTER**AISVPVGAGTLGR**IMDVLGRPIDEAGDVQASDHWEIHR**GAPSYEDQSSSTELLETGIK**VIDLMCPFAKGGK**VGLFGGAGVGKTVNMMELINNIAK**AHSGLSVFAGVGERTR EGNDFYHEMKDSNVLDK**VAMVYGQMNEPPGNR**LR**VALTGLTMAEYFR**DEKDASGKGKDVLL FVDNIYR**YTLAGTEVSALLGR**MPSAVGYQPTLAEEMGVLQERITSTKSGSITSIQAVYVPA DDLTDPSPATTFAHLDSTVTLSR**NIASLGIYPAVDPLDSTSR**QMDPLVIGHEHYDTAQR**VQQTLQK**YKELK**DIIAILGMDELSEEDK**QSVSRARKIER**FFSQPFHVAEVFTGSPGK**YVSLKD TIRGFKAICDGEYDHLPEQAFYMVGSIEEAVEKANKMSAKA

Matched Peptide: AISVPVGAGTLGR, DIIAILGMDELSEEDK, FFSQPFHVAEVFTGSPGK, GAPSYEDQSSSTELLETGIK (2X), NIASLGIYPAVDPLDSTSR, TVNMMELINNIAK, VALTGLTMAEYFR, VAMVYGQMNEPPGNR, VGLFGGAGVGK, VQQTLQK (4X), YTLAGTEVSALLGR

XAUB_17520. ATP-dependent protease ATP-binding subunit

MPNPDSSTMTPREIVQELDRHIVGQHDAKRAVAIALRNRWRRMQLPEELRNEVMPKNILMI GPTGVGKTEIARRLATLVNAPFVKVEATRFTEVGYVGKDVEQIIRDLADTAVKLYREQAKV RVRNQAEERAEDRILDALLPRRATGIGFDPEAARNEPSSQDNDTRIKFRRMLRNGELDERE IELEVAVNASMDIMTPPGMEEMGQQLRQMFSNLGSGKSQKRKLTIKAARPLLIEEEAGKLV NEDDVRTAAIEACEQHGIVFIDEIDKVAKR**GEAGSSGGDVSR**EGVQR**DLLPLVEGSNVSTK**

YGTVKTDHILFIASGAFHLAKPSDLIPELQGRFPIRVELTALTKADFVRILTEPKAALIKQ
YEALLQTEGVALTFASDAVDRLAEIAAQVNERQENIGARRLHTVLERLLDVLSYEAPDRDG
QSVTVDAAYVDAQLGELVQDPDLSRYIL
Matched Peptide: DLLPLVEGSNVSTK, GEAGSSGGDVSR

XAUB_15880. dihydrolipoamide dehydrogenase

M**SEQEQFDVVVIGAGPAGYHAAIR**AAQLGMKVACIDAALGKDGKPALGGTCLRVGCIPSKA
LLDSSRQFWNMGHLFGDHGISFNDAKMDVPTMIGRKDKIVK**QFTGGIAMLFK**ANK**ITPYYG
FGQLQPGNIVK**VTQPEGGEIELKGTNVILAAGSESIELPFAK**FDGDTIVDNVGGLDFTAVP
K**RLAVIGAGVIGLELGSVWKRLGAEVTILEALPDFLALADAEVAKTALKEFK**KQGLDIK**LG
AKVGK**TEITGSGDAK**QVVLSYTDAAGEQTLTVDK**LLVAVGR**KAATKNLLAEGTGVKVTDRG
QIEVDGHCHTGVDGVWAIGDCVRGPMLAHKGFEEGIAVVELIAGLPGHVNFDTIPWVIYTE
PEIAWVGKTEQQLK**AEGVAYKAGSFPFAAIGRAVAMGEPAGFVK**VIADAETDRVLGMHLVG
VGVSELVHEGVLTMEFNGSADDLARICHAHPTLSEAIHDAAMAVSKRAIHKAN

Matched Peptide: AEGVAYK (2X), AGSFPFAAIGR (2X), AVAMGEPAGFVK, FDGDTIVDNVGGLDFTAVPK, ITPYYGFGQLQPGNIVK, KQGLDIK, LLVAVGR (2X), QFTGGIAMLFK (3X), SEQEQFDVVVIGAGPAGYHAAIR (4X), TEITGSGDAK (6X), VACIDAALGK (2X), VIADAETDR, VTQPEGGEIELK, AGSFPFAAIGR, AGSFPFAAIGR

XAUB_16560. cystathionine β-synthase

MAIHSSVLELIGNTPIVK**AQRLDTGVCELFL**KLEANNPGGSIKDRIGLSMIEAAERRGDLK
PGATLVEGTAGNTGLGLALVAQQKGYQLILVVPDKMSREKIFNLK**AMGANVVLTRSDVA**KG
HPEYYQDLAARIAAETPGAYFINQFGNPDNPAAHEFGTGPEILAQMDGRLDAIVFGCGSSG
TMTGLSRAFASASPHTELVLADPVGSILTQYIEEGTVSEKSGSWLVEGIGEDFLPDISDFS
RVKKAYSISDAESFHTARELLAKEGILGGSSTGTLLAAALKYCREQTMPKRVLVFVCDTGN
K**YLSKMYNDYWMLDNGFLE**RPQHGDLRDLILRPYNKRDTVVVGPKDLLTTAYQRMKLYDVS
QLPVIDDGELVGIVDESDVLLHVYGDEARFRDPISTAMVSKLDRLDVASPIEALLPVFDR**G
QVAIVMDGTQFLGLITRIDLLNYLRRRVQ**

Matched Peptide: AQRLDTGVCELFL, AMGANVVLTRSDVA, YLSKMYNDYWMLDNGFLE, GQVAIVMDGTQFLGLITRIDLLNYLRRRVQ

XAUB_12060. aldehyde dehydrogenase

MKILDWSQLDGAAR**TDALTRPVQTVAARTRDAVAALIADV**RTRGDAALREITARFDGMSLD SFAVSEAEFVTAEAAVPPELRQAMQDAVARIDTFHRAGMSQGYAVETAPGVVCEKIVRPIG RVGLYVPAGSAPLPSTALMLGVPARLAGCREVVLCTPPRKDGSVDPAVLVAAQLTGVRRVF KLGGAQAIAAMAYGTESLPSCDKLFGPGNSYVTEAKQQVAQSGAAAIDMPAGPSEVLVIAD AGAQPAFVAADLLSQAEHGPDSQVLLLSDSDALIDAVQAQLEVQLAQLSRADIARQALAQS RLIK**VQTLDEAFAISNRYAPEHLILALR**EPRAWLEHVEAAGSVFLGDYTPEALGDYCSGTN HVLPTSGAARAYSGVSVASFQNMVSVQAASKAGIDGIGECALLLARAEGLDAHANAVALRM GAAA

Matched Peptide: TDALTRPVQTVAARTRDAVAALIADV, VQTLDEAFAISNRYAPEHLILALR (2X)

XAUB_21320. aldehyde dehydrogenase

MDGPGGRHPRLQQARVAAGAGSIRLLRRSDCHPCIRRTACAPQRWPRACGAGAGWRGRRNH ALERAAGIAVLVAKPSPETPIDAYILAECISAAGVPDGVFNLLPAGREVGEQLIRHPHVDK **VSFTGSTQAGRSIGIACAE**RLARVGLELGGKSAAIVLEDADLAKMLPTLVPYSMPIAGQVC FSLTRILVPTQRREEILQAYCTALGAVKLGDPFAADTGMGPLALGRQLERVQSYIAKGKAE GARLVMGGSRPAHLSRGFFVEPTVFAEVTPDMAIAREEIFGPVVSFIDYHDEADLIAKANA SDYGLHGTIYSEDAERAYRIARRVRSGSHAINGMWVDISMAFGGFKHSGIGREGGIEGLHA FLETRTLYLS

Matched Peptide: VSFTGSTQAGRSIGIACAE (3X)

Spot20

XAUB_15880. Dihydrolipoamide dehydrogenase

M**SEQEQFDVVVIGAGPAGYHAAIR**AAQLGMKVACIDAALGKDGKPALGGTCLRVGCIPSKA LLDSSRQFWNMGHLFGDHGISFNDAKMDVPTMIGRKDKIVK**QFTGGIAMLFK**ANK**ITPYYG FGQLQPGNIVK**VTQPEGGEIELKGTNVILAAGSESIELPFAK**FDGDTIVDNVGGLDFTAVP K**RLAVIGAGVIGLELGSVWKRLGAEVTILEALPDFLALADAEVAKTALKEFK**KQGLDIK**LG AKVGK**TEITGSGDAK**QVVLSYTDAAGEQTLTVDK**LLVAVGR**KAATKNLLAEGTGVKVTDRG QIEVDGHCHTGVDGVWAIGDCVRGPMLAHKGFEEGIAVVELIAGLPGHVNFDTIPWVIYTE PEIAWVGKTEQQLK**AEGVAYKAGSFPFAAIGRAVAMGEPAGFVK**VIADAETDRVLGMHLVG VGVSELVHEGVLTMEFNGSADDLARICHAHPTLSEAIHDAAMAVSKRAIHKAN

Matched Peptide: AEGVAYK (2X), AGSFPFAAIGR (2X), AVAMGEPAGFVK, FDGDTIVDNVGGLDFTAVPK, ITPYYGFGQLQPGNIVK,

KQGLDIK, LLVAVGR (2X), QFTGGIAMLFK (3X), SEQEQFDVVVIGAGPAGYHAAIR (4X), TEITGSGDAK (6X), VACIDAALGK (2X), VIADAETDR, VTQPEGGEIELK, AGSFPFAAIGR, AGSFPFAAIGR

Spot21

XAUB_15890. Dihydrolipoamide acetyltransferase

MATEVKVPVLPESVSDATIASWHKKAGEAVKRDENLVDLETDKVVLEVPSPVDGVLKEIKF
DTGSTVTSNQILAIIEEGAVAAAAPAEEK**QAAAPAATAAAPAAAPAPAAAAAPAAASK**S**AA**
DSLPPGARFSAITQGVDPSQVEGTGRR**GAVTKEDIVNFAK**AGGVGK**ASGARPEER**VPMTRV
RKTIAKRLMESK**NSTAMLTTFNEVNLAK**VSAAR**KELQDEFQK**AHGIK**LGFMSFFVK**A**AANA**
LQRFPLVNASIDGDDIIYHGYSDISIAVSTDK**GLVTPVLR**NVER**QSFADVEQGIADYAAK**A
RAGKLGLDDLQGGTFTITNGGTFGSLLSTPIINPPQSAILGMHAIKERPIAENGQVVIAPM
MYLALSYDHRIIDGKDSVQFLVDIK**NQLENPGR**MLFGL

Matched Peptide: AAANALQR, ASGARPEER, EDIVNFAK, FSAITQGVDPSQVEGTGR, GAVTKEDIVNFAK, GLVTPVLR, KELQDEFQK, LGFMSFFVK (6X), NQLENPGR, NSTAMLTTFNEVNLAK (3X), QAAAPAATAAAPAAAPAPAAAAAPAAASK (2X), QSFADVEQGIADYAAK, SAADSLPPGAR

Spot22

XAUB_15890. Dihydrolipoamide acetyltransferase

MATEVKVPVLPESVSDATIASWHKKAGEAVKRDENLVDLETDKVVLEVPSPVDGVLKEIKF
DTGSTVTSNQILAIIEEGAVAAAAPAEEK**QAAAPAATAAAPAAAPAPAAAAAPAAASK**S**AA**
DSLPPGARFSAITQGVDPSQVEGTGRR**GAVTKEDIVNFAK**AGGVGK**ASGARPEER**VPMTRV
RKTIAKRLMESK**NSTAMLTTFNEVNLAK**VSAAR**KELQDEFQK**AHGIK**LGFMSFFVK**A**AANA**
LQRFPLVNASIDGDDIIYHGYSDISIAVSTDK**GLVTPVLR**NVER**QSFADVEQGIADYAAK**A
RAGKLGLDDLQGGTFTITNGGTFGSLLSTPIINPPQSAILGMHAIKERPIAENGQVVIAPM
MYLALSYDHRIIDGKDSVQFLVDIK**NQLENPGR**MLFGL

Matched Peptide: AAANALQR, ASGARPEER, EDIVNFAK, FSAITQGVDPSQVEGTGR, GAVTKEDIVNFAK, GLVTPVLR, KELQDEFQK, LGFMSFFVK (6X), NQLENPGR, NSTAMLTTFNEVNLAK (3X), QAAAPAATAAAPAAAPAPAAAAAPAAASK (2X), QSFADVEQGIADYAAK, SAADSLPPGAR

Spot23

XAUB_38740. Betaine aldehyde dehydrogenase

MPRFPDQLLYIGGRYVPARGGHTFEVVNPATGEVLANVHNAGADDLDAAVDSAKAGQQR**WAALTTVER**SRILLRAVALLRERNDALAELETLNTGKPLSETRSVDVVTGADVLEYYAGVAQALQGAQVPLR**EGSFFYTR**HEPLGVVGAIGAWNYPIQIALWK**AAPALAAGNAMIFKPSEVTPLTALK**LAEIFTEAGLPDGVFNVLPGDGASVGTALTEHPQIEK**ISFTGGTATGR**KVMASASSSSLKEVTMELGGKSPLIVCADADLDLAADIAMMANFYSSGQVCTNGTRVFVPRALR**HAFEAR**LLARVQRIHIGDPLDER**TTFGPLVSAAHMQR**VLEHIEQGKAEGARLLCGGERLQDGALAQGYYVAPTIFSDCTDVMTIVREEIFGPVLSLLTYDDEDEAVTR**ANATTYGLAAGVVTPDLAR**AHRLIHRLEAGICWVNTWGESPAPMPVGGYKQSGVGRENGLATLQAYTRTK**SVQIELER**YASVF

Matched Peptide: AAPALAAGNAMIFKPSEVTPLTALK, ANATTYGLAAGVVTPDLAR, EGSFFYTR, HAFEAR (2X), ISFTGGTATGR, SVQIELER, TTFGPLVSAAHMQR, WAALTTVER (2X)

XAUB_30070. Virulence protein

MRRLEMWSALCGAAMVTLSGMAAAQAPEPVSHGR**FEQVPVLMPR**GEPQR**VVIWLAGAGNAAK**RQAQAESLRADGAMVAVVDTAHLYAVLRKAGGTCTFSVGDVENFSR**YVQAFYHIPTYR**LPLLVGDGEGAALAYAIAAQAKPHVLAGVLTDGLCPATVSNQAICQPGVRPGTNTLLPVPLQIPWVLAASQDKRCPAAAEEGFLKQVPQARTFRRSAQGDILPGLRAAVRSLGEQKGVALPPPPGGLADLPVVELPAKPDGDSDDDTFVIFVSGDGGWAGLDEEVADALAAQGIPVVGLDSLRYFWTERTPQGFADDLDRIARIYAQRWDRQR**VVLIGFSQGADVLPAAINK**LPAATKQNLRMTALLSVGKLADYEFHVSNWLGSDDEGLPIAPEVQRLPAGTTVCIYGQDDDDALCPSLPANVARRVALPGDHHFKGDYATLAKTIMDQLHALSKP

Matched Peptide: FEQVPVLMPR, VVIWLAGAGNAAK (2X), VVLIGFSQGADVLPAAINK (3X), YVQAFYHIPTYR

Spot24

XAUB_20360. Pyruvate kinase

MPSSVRDVCVKFRGFYREAIMKERQRRTKILATLGPATDAPGVLDTLFK**AGVNVVRLNFSHGDPSGQAK**RAAEVRAAAAR**VGAEVGILADLPGPK**IRIERFAEGKIKLTMGDRFDLVADANAPAGDQTQVGVSYLGLPQDVTAGDVLLLDDGLMQLQVTDVQGARIVTK**VLNDGVLSDR**KGLN

K**QGGGLSLGALTERDKELIGIVAK**IGVDFIAVSFCRNAQDMHDARQIAQQHGCDAQLVSKI
ERTEAIENLVEIVEASDVVMVAR**GDLGVEIGDAELPGLQK**KIIR**ESLAQNK**VVITATQMLQ
SMVESPIPTRAEVLDVANAVIDGTDAVMLSAETAAGAYPVR**AVEAMAR**ICLGAEHQFEFDT
DFEAAQRNLQRADQAIAMATMFLSEHIGLGGVVALTESGGTPRFLSRFR**SNMPIYAFTR**HD
GARRQMAMMR**GVFPISFDSR**GLTPREAARAAIRLLVENERMGPGDRVVFTSGEHMETHGAT
NTLR**LLEVGEDGR**ATGLGEL

Matched Peptide: AGVNVVR, AVEAMAR, DKELIGIVAK (6X), ESLAQNK (3X), GDLGVEIGDAELPGLQK (4X), GVFPISFDSR (5X), LLEVGEDGR, LNFSHGDPSGQAK, QGGGLSLGALTER (6X), SNMPIYAFTR (3X), VGAEVGILADLPGPK (6X), VLNDGVLSDR (2X)

XAUB_14750. Chaperonin GroEL

MAAKDIRFGEDARTRMVRGVNVLANAVKATLGPKGRNVVLEKSFGAPTITKDGVSVAKEIELAD
KFENMGAQMVKEVASKTNDNAGDGTTTATVLAQALIREGAKAVAAGMNPMDLKRGIDQAVKAAV
VELK**NISKPTTDDK**AIAQVGTISANSDESIGNIIAEAMQKVGKEGVITVEEGSGLENELDVVEG
MQFDRGYLSPYFINNQQSQSADLDDPFILLHDKKISNVR**DLLPVLEGVAK**AGKPLLIVAEEVEG
EALATLVVNTIRGIVKVVAVKAPGFGDRRKAMLEDMAVLTGGTVISEEVGLALEKATIKDLGRA
KKVQVSKENTTIIDGAGDTAAIESRVGQIKTQIEDTSSDYDREKLQERVAKLAGGVAVIKVGAS
TEIEMKEKKARVEDALHATR**AAVEEGVVPGGGVALVRALVAVGELKGANEDQTHGIQIALR**AME
APLR**EIVANAGEEPSVILNK**VKEGSGNYGYNAANGEFGDMVQFGILDPTKVTRSALQNAASIAG
LMITTEAMVADAPKKDEPALPAGGGMGGMDF

Matched Peptide: AAVEEGVVPGGGVALVR (2X), DLLPVLEGVAK, EIVANAGEEPSVILNK, GANEDQTHGIQIALR (2X), NISKPTTDDK (2X), ALVAVGELK

XAUB_05850. GDP-mannose pyrophosphorylase

MSDVLPIILSGGSGTRLWPLSRESYPK**QFLPLVGDK**SMLQSTWLRAAPVAGHAPIVVANEE
HRFMAAEQLQQLGVKPSAILLEPKGR**NTAPAIAVAALEATRDGADPLLLVLPSDHVIGNEA**
AFQAAVKVAAEAAAQGKLVTFGIKPTAPETGYGYIKAGAGTAASAVERFVEKPDLATAQSY
LASGEYYWNSGMFLFRASRYLEELRKFHPAIADACQKAWENGKRDADFTRLDKDAFAASPS
DSIDYAVMEKTADAVVVPLDAGWNDVGSWSSLLDVSNQDAQGNAHHGDVIQLDCQNTYAYG
SRLIAMVGLEDVVVVETPDAVLVGHRDRIQEVKDVVSQIKTAGRSEATWHRKVYRPWGAYD

SIDMGQRHQVKRITVKPGAVLSLQMHHHRAEHWIVVSGTAEVTRGEEVLLLTENQSTYIPL GVTHRLRNPGKLPLELIEVQSGSYLGEDDIVR**FEDTYGR**A

Matched Peptide: DGADPLLLVLPSDHVIGNEAAFQAAVK (2X), FEDTYGR, LVTFGIKPTAPETGYGYIK, NTAPAIAVAALEATR (4X), QFLPLVGDK (2X), VAAEAAAQGK (2X)

XAUB_10980. Outer membrane receptor for transport of vitamin B

MSLSSVSPPRAVLAVGLSLCVATVAHAEAVIDLDHVVVTASR**TAQTQDQTLAPVTVIDRAQIER**RQVNSL QDLLRGEAGVSLANNGGPGKATSLFLRGTEADHVVVLIDGVRIGSATAGGAALQDLPIEQIERIEIVRGP FSSLYGSEALGGVIQIFTRRPQGGFVPTLSVAAGSDNARRYGAGIAGRSPGDLSDAGGWYSVNAVHDETD GINAYLDTSSSAYDPDRDGYRNDSLSAQGGWRFNRQWDADVHVLRAQSRNEYDGSAFGGNLSKGVQQAIG GRVRYAPSDALK**LTASVGSSADLSDAYYQGAYLSTYDTRRK**QGALQADLDTGPGLLTVGFDWQRDAIASS DNYDSDSRIDRAAFAQWQQTFGSQSLQASLRRNDNSQFGGKTTGSLLWGWDVAEHLRLTASYGTAFKAPT FNELYYPDYGNPLLGPETSKSAELGLRGRYDWGTWTLSAFQTRIDDLIAYDGSLVDATHPFGQPNNIDRA RIRGVEAGYDTELAGWTLRSALTWLAPQADGEVNHGNWLPRRARQSGRIDADRSIGVFGIGASLFGSGAR YDDLANTDRLAGYGLLDLRVSYAVNADWKVQFAANNVFDR**HYETARWYAQPGRNYLLTFRYQPAH**

Matched Peptide: TAQTQDQTLAPVTVIDRAQIER, LTASVGSSADLSDAYYQGAYLSTYDTRRK (2X), HYETARWYAQPGRNYLLTFRYQPAH

Spot25

XAUB_4147. Acyl-carrier-protein S-malonyltransferase

MTESTLAFVFPGQGSQSLGMLAELSELHPQIRETFAEASEGAGVDLWALSQGGPEEMLNRT EYTQPALLAAGVAVWRLWTAQRGQRPALLAGHSLGEYTALVAAGALSLHDGAHLVRLRGQF MQAAAPAGVGAMAAVLGAEDAVVQEVCAQASGSEVVVPANFNSPGQIVIGGHAAAVDRALG LLAERGVRKAVKLAVSVPSHTPLMREAANQLGEAMAGLTWHAPQIPVVQNVDARVHEGNTA ICQALVEQLYLPVQWTGCVQAFASQGITR**IAECGPGKVLSGLIK**RIDKRLDARPLATPADY AGALEAWAH

Matched Peptide: IAECGPGK (2X), VLSGLIK

Spot26

XAUB_20360. Pyruvate kinase

MPSSVRDVCVK**FRGFYREAIMKE**RQRRTKILATLGPATDAPGVLDTLFK**AGVNVVRLNFSH GDPSGQA**K**RAAEVRAAAAR**VGAEVGILADLPGPK**IRIERFAEGKIKLTMGD**RFDLVADANA PAGDQTQVGVSYLGLPQDVTAGDVLLLDDGLMQLQVTDVQGAR**IVTKVLNDGVLSD**RKGLN KQGGGLSLGALTERDKELIGIVAKIGVDFIAVSFCRNAQDMHDARQIAQQHGCDAQLVSKI

ERTEAIENLVEIVEASDVVMVAR**<u>GDLGVEIGDAELPGLQ</u>**K**<u>KIIRESLAQN</u>**K**<u>VVITATQMLQSMVESPIPT</u>**R**<u>AEVLDVANAVIDGTDAVMLSAETAAGAYPV</u>**RAVEAMARICLGAEHQFEFDTDFEAAQRNLQRADQAIAMATMFLSEHIGLGGVVALTESGGTPR**<u>FLSRFRSNMPIYAFTRHDGA</u>**RRQMAMMRGVFPISFDSRGLTPREAARAAIRLLVENERMGPGDRVVFTSGEHMETHGATNTLR**<u>LLEVGEDGRATGLGEL</u>**

Matched Peptide: FRGFYREAIMKE, AGVNVVRLNFSHGDPSGQA, IRIERFAEGKIKLTMGD, IVTKVLNDGVLSD, LLEVGEDGRATGLGEL, VVITATQMLQSMVESPIPT, KIIRESLAQN (2X), AEVLDVANAVIDGTDAVMLSAETAAGAYPV, SNMPIYAFTRHDGA, RAAEVRAAAAR, GDLGVEIGDAELPGLQ (3X), FLSRFRSNMPIYAFTRHDGA

XAUB_08690. Bifunctional GMP synthase/glutamine amidotransferase protein

MTNIHTDK**ILILDFGAQYTQLIARRI**REIGVYCEIWAWDHDPSEIAGFGAKGIILSGGPESTTLPGAPVAPQEVFDSGLPVFGICYGMQTLAAQLGGATEAADQREFGHAEADVVAADALFAGLTDHAGASRLNVWMSHGDHVSQVPPGFTITATTDRIPVAAMSNEAKRWYGVQFHPEVTHTLQGQTLLRRFVVDVCGCQTLWTAANIIEDQIARVREQVGDDEVILGLSGGVDSSVVAALLHKAIGDKLTCVFVDTGLLRWQEGDQVMAMFAEYMGVK**<u>VIRINAADRYFAKLEGVSDPEAK</u>**RKIIGNLFVEIFDEESNKLANAKWLAQGTIYPDVIESAGSKTGKAHVIKSHHNVGGLPEHMKLGLVEPLRELFKDEVRRLGVELGLPRTMVYRHPFPGPGLGVRILGEVKREYAELLAKADAIFIDELRKADLYDTTSQAFAVFLPVKSVGVVGDARAYEWVIALRAVETIDFMTAHWAHLPYDFLGTVSNR**<u>IINELRGVSRVVYDISGKPPATIEWE</u>**

Matched Peptide: ILILDFGAQYTQLIARRI (2X), IINELRGVSRVVYDISGKPPATIEWE (3x), VIRINAADRYFAKLEGVSDPEAK

Spot27

XAUB_21500. Glucose-6-phosphate isomerase

MTHTNGFDALHAHAQRLR**<u>GAAIPALLAAEPERPTQYA</u>**R**<u>QVGPLYFNFARQKYDRAALDALFAIARA</u>**RDLSGAFQRLFR**<u>GEQVNVTEQRAALHTALRGDLTDAPVASEAYATAAEV</u>**RQRMGALIQQLEATEVTDIVSVGIGGSDLGPRLVADALR**<u>PVAGARLRVHFVSNVDGAAMQ</u>**R**<u>TLATLDPARTAGILIS</u>**K**<u>TFGTQETLLNGSILHAWLGGSE</u>**R**<u>LYAVSANPERAAKAFDIAPG</u>**RVLPMWDWVGGR**<u>YSLWSAVGFPIALAIGFE</u>**RFEQLLEGAAQFDAHVLNTLLEENVAVLHGLTAVWNRNLLGSATHAVMTYDQRLALLPAYLQQLVMESLGKRVKLDGSAVDSDTVSVWWGGAGTDVQHSFFQALHQGTSVVPADFIGTVHNDDPYAENHTALMANVLAQTEALANGQDSSDPHRSYPGGRP

STVILLDALTPQALGALISMYEHSVYVQSVMWGINAFDQFGVELGK**QLASQLLPAL**K**GESADVADPVTRELLNKLRG**

Matched Peptide: GAAIPALLAAEPERPTQYA (3X), GEQVNVTEQRAALHTALRGDLTDAPVASEAYATAAEV, QLASQLLPAL, LYAVSANPERAAKAFDIAPG (2X), TFGTQETLLNGSILHAWLGGSE, GESADVADPVTRELLNKLRG, YSLWSAVGFPIALAIGFE, PVAGARLRVHFVSNVDGAAMQ (2X), TLATLDPARTAGILIS, TLATLDPA

Spot28

XAUB_09300. Adenylosuccinate lyase

M**SDSALLALSPLDGR**YASK**VDALRPIFSEYGLIK**ERVKVEIEWLLALAAEPGIAELAPFSESAAQRLR**ALADGFSVIHAARVKEIER**TTNHDVK**AVEYFIK**EQLTDDAELGPALEFVHFACTSEDINNLSYGLMLEQARREVLLPTLDGIVASLRSLAHAQAGQPMLSRTHGQTASPTTLGK**EIANVVAR**LERQRK**QIAAVELTGK**INGAVGNYNAHLVSYPQLDWAAFAQRFVESLGLVFNPYTTQIEPHDNVAEIGDAAR**RANTILIDLAR**DIWGYISLGYFKQK**LKEGEVGSSTMPH**KVNPIDFENAEGNFGIANALFEHFSAKLPISRWQR**DLTDSTVLRALGTAFGHTQVALDSLAK**GLGKLTVNPERLDADLDAAWEVLAEAVQTVMRR**HGLPNPYEQLK**ALTR**GQGITAASMQAFVESLQLPEDDKQR**LR**ALTPGGYTGLAEQLAR**AI

Matched Peptide: ALADGFSVIHAAR (2X), ALGTAFGHTQVALDSLAK, ALTPGGYTGLAEQLAR, ANTILIDLAR, AVEYFIK, DLTDSTVLR, EIANVVAR, GQGITAASMQAFVESLQLPEDDKQR (2X), HGLPNPYEQLK, LKEGEVGSSTMPHK (2X), QIAAVELTGK (2X), RANTILIDLAR, SDSALLALSPLDGR (2X), VDALRPIFSEYGLIK (5X), VKEIER

Spot29

XAUB_08180. NADH dehydrogenase gamma subunit

MSDSALLALSPLDGRYASK**VDALRPIFSEYGLIKERVK**VEIEWLLALAAEPGIAELAPFSESAAQRLR**ALADGFSVIHAARVKEIERTTNHDVKAVEYFIK**EQLTDDAELGPALEFVHFACTSEDINNLSYGLMLEQAR**REVLLPTLDGIVASLRSLAHAQAGQPMLSR**THGQTASPTTLGKEIANVVARLER**QRKQIAAVELTGK**INGAVGNYNAHLVSYPQLDWAAFAQRFVESLGLVFNPYTTQIEPHDNVAEIGDAARR**ANTILIDLARDIWGYISLGYFK**QK**LKEGEVGSSTMPHK**VNPIDFENAEGNFGIANALFEHFSAK**LPISRWQRDLTDSTVLRALGTAFGHTQVALDSLAK**GLGK

LTVNPERLDADLDAAWEVLAEAVQTVMRRHGLPNPYEQLKALTRGQGITAASMQAFVESLQ LPEDDKQRLR**ALTPGGYTGLAEQLAR**AI

Matched Peptide: VDALRPIFSEYGLIKERVK, VKEIERTTNHDVKAVEYFIK, REVLLPTLDGIVASLRSLAHAQAGQPMLSR(2X), ANTILIDLARDIWGYISLGYFK, VQTVMRRHGLPNPYEQLKALTRGQGITAASMQAFVESLQLPEDDK, QRKQIAAVELTGK, LPISRWQRDLTDSTVLR, ALGTAFGHTQVALDSLAK, LKEGEVGSSTMPHK, LTVNPERLDADLDAAWEVLAEAVQTVMR, ALTPGGYTGLAEQLAR, SLAHAQAGQPMLSR, ALADGFSVIHAAR

Spot30

XAUB_30230. Acetyl-CoA C-acetyltransferase

MSDIVIVAAKRTAIGSFLGQFNAVPAPTLAAAAIQGALAQSGIAPADISEVIVGCVLPANL GQAPAR**QAAIAAGIPTSTGATTINK**VCGSGMK**AIMLGHDLIKAGSASIVVAGGMESMSNAP HLLPNSR**TGNRYGNFQAVDHMAWDGLTNPYDGQAMGVFGEATAEKFGFSR**ADQDAFAIASV ER**AQAAQR**SGAFAEEIVPVTVATRKGEVVVDSDEQPGK**SDVAK**IPTLKPAFK**KDGSVTAAS SSSISDGAAITVLMSADDAQRRGVTPLARIVGHVTHAQEPEWFTTAPVAAIQSLVSKLGWR LDDVDLFEINEAFAVVAMAPIR**QLGIAHDK**VNVHGGACALGHPIGASGAR**LVVTLVNALR**S RGGKRGIATLCIGGGEATAIAIELI

Matched Peptide: ADQDAFAIASVER (3X), AGSASIVVAGGMESMSNAPHLLPNSR, AIMLGHDLIK, IPTLKPAFK, KGEVVVDSDEQPGK, LVVTLVNALR (4X), QAAIAAGIPTSTGATTINK, QLGIAHDK, SGAFAEEIVPVTVATR

Spot31

XAUB_14870. phosphate acetyltransferase

MSTDDFK**QAALDYHRQQPAGKIKVTATKPMLTQ**RDLSLAYSPGVAFACEAIVEDATQASEL TAR**GNLVAVISNGTAVLGLGNIGPLAS**KPVMEGKGVLFQKFAGIDVFDIEINENDPDKLVD IIASLEPTFGGINLEDIKAPECFIVERK**LRERMNIPVFHDDQHGTAIIVGAAVLNALVVTG K**KIEEVKLATTGMGAAGISCVNMLVSLGLKPENILALDRDGVIHTGRTDLDPDKQRYARDT DKRTLAEIVEGADIFLGLSAAGILKPEMVASMARQPVIFALANPNPEITPEAAKAVRPDCI IGTGRSDYPNQINNVLCFPYLFRGALDVGATGINEEMKIACVKAIAAMARREASDLGAAYG GETPSFGPEYLIPRPLDPRLLVELSSAVAQAAMDSGVATRPITDMEAYRDKLGQFVYRTSL MMKPVYDRARADKQRVVYAEGEEEVVLRAVQNVVDEGLAYPILIGRPDVIEARIERMGLRL

TAGVDFEITNILDDPRFNDYWQYYHALTERRGVTVTAAKELMRSRPTLIAAVMVARGEADA
MLSGVVGRFHKKLGYARSVIPLEPRVSSTSAMTGVINQLGVFFFLDTHVQEDPTVEQVVEA
TLQAAYRLKLFGIEPNIALLSHSNFGSHDSRDALKMRQVREALLKRKPELNIDGEMQGDTA
WDEALRKQIMPNSTLKGRANLFVLPNLEAANIAYNLVRVFTDGVAIGPILMGISKPVHILT
TSATSRRVMNMTAIAAVDAQIR**RQRDAEKTAADKGSD**

Matched Peptide: QAALDYHRQQPAGKIKVTATKPMLTQ (2X), GNLVAVISNGTAVLGLGNIGPLAS, LRERMNIPVFHDDQHGTAIIVGAAVLNALVVTGK, RQRDAEKTAADKGSD (3X)

Spot32

XAUB_38250. Tail-specific protease

MTYNVSSCMKAGLLALVLTTPMALLARADTALPAAATPDQATATK**LVYGLLSDSR**YAYRPR
TLDEAMSKDVFKRYLETLDGGKQFFTQADIDSFAPLQAGVGDALRGGNLEPAFQVFSVYKK
RVDQRVKYARDLLKQDFDFSGNDKFEYDRKDVPWAADDKQLDVLWRQSVMNDWLRLKLAGK
KPDDIRKTLDKRYVALADSVKQLKGEDVFQFFVNAYTNAVDPHTDYFTPRTAETFNQQMSL
SLEGIGAQLQKQDDMVVIR**EVIPGGPAAVDGTLKPGDR**IVGVGQAKSGAIEDVIGWRIDDV
VAKIRGSKDTQVR**LEYIPAESGIDGK**HRTVTLTRQKVR**LAEQAAK**GETITLPATGSEPQRR
IGIIK**LPGFYQDFEGR**RRNATDYASATRDVAKLLAGFK**TDKVDGVVLDLR**NNGGGSLDEAI
ELTGLFIEQGPVVQVRESGGRVTVNGDSDPKVAWDGPLAVLINR**GSASASEIFAGAIQDYG**
RGLVIGETSFGKGTVQNIVDLDRWPAAEGQRYGQVK**LTIAQFFR**VSGSSTQHKGVVPDIAF
PASVDATEFGESTYDNALPWTRIAAAPHTQYGNFAALLPKLQALHTARIATDKEFQWWEED
VK**QFRDEK**AKK**YISLNEAER**VAERQKQDQQRKDRQQIRK**QLGLPLDPLAEDSDDGLTGNE**R
DIVKDTAREKAAEKRPDPLLHESAAILADALGLLSQDKPLSAQVLPKSTSPGRWAD

Matched Peptide: EVIPGGPAAVDGTLKPGDR (2X), GLVIGETSFGK, GSASASEIFAGAIQDYGR, GTVQNIVDLDR, LAEQAAK (2X), LEYIPAESGIDGK, LPGFYQDFEGR (2X), LTIAQFFR (3X), LVYGLLSDSR, QFRDEK, QLGLPLDPLAEDSDDGLTGNER, TDKVDGVVLDLR, VDGVVLDLR (2X), YISLNEAER (2X)

Spot33

XAUB_03410. Formate dehydrogenase acessory protein

MTSPSSHSVR**PGTVVRTVRRHRGGRSATVQD**RVAAEMPVAFIYNGVPFAVMVATPEDLEDF
ALGFSLSEGIVDHAQDLRVIAVETFLEGASLQIEIPPER**AAALDQRRRNLDGRSGCGVCGN
ESIEAVL**RVPPVLHSSLQIDVDALAHALDALHARQPIAAQTGAVHAAGWADAQGNVQLVRE
DVGRHNALDK**LIGALARARIDASHGFAVVTSRASYEMAM**KAAQARIPLLAAISAPTALAIS
LAESAGLTLIGFARDHDCVVYSHPQRLDLGVAVGEPA

Matched Peptide: PGTVVRTVRRHRGGRSATVQD (4X), AAALDQRRRNLDGRSGCGVCGNESIEAVL, LIGALARARIDASHGFAVVTSRASYEMAM (2X)
