# Supplementary material for: Periplasm-enriched fractions from Xanthomonas citri subsp. citri type A and X. fuscans subsp. aurantifolii type B present distinct proteomic profiles under in vitro pathogenicity induction
Source: PLoS One. 2020 Dec 18;15(12):e0243867. doi: 10.1371/journal.pone.0243867 (PMC7748154; doi:10.1371/journal.pone.0243867)
Supplement: S3 Table — XAC and XauB were grown in NB medium, and periplasm-enriched fractions, extracted from both bacteria, were resolved on 2-DE (p< 0.05), and proteins from differential spots were identified by ESI-Q-TOF. The cultivation times were for XAC 25 h and for XauB 40 h. a Proteins identified using Mascot with XAC or XauB databases (NCBI); not all XauB proteins identified were included, only those ones that presented the highest Mascot scores and/or molecular weight (MW) and isoelectric point (pI) more compatible with experimental values; b Exclusive peptides count determined for some spots using Scaffold™ software (Proteome Software Inc., Portland, OR) for 100% protein identification probability; c Theoretical MW and pI of the matched protein obtained from the NCBI database; d Experimental molecular weight (MW) and isoelectric point (pI) calculated by Image Master Platinum software (GE Healthcare) based on the position of the spot on 2-DE; e Proteins clustering according to “Xanthomonas axonopodis pv. citri Main Chromosome and Plasmid Gene List” at NCBI [6]: I) Intermediary metabolism, II) Biosynthesis of small molecules, III) Macromolecule metabolism, IV) Cell structure, V) Cellular processes, VI) Mobile genetic elements, VII) Pathogenicity, virulence and adaptation, VIII) Hypothetical, IX) ORFs with undefined category; f Predicted cellular location of proteins by pSortP 3.0 and SecretomeP 2.0. P, M, and C correspond respectively to periplasm, membrane, and cytoplasm location. Signal (+) indicates the presence of signal peptide according to SignalPeptide 2.0. (PDF) [file pone.0243867.s006.pdf]

Table S3. Proteins identified by ESI-Q-TOF in spots exclusively presented by XAC or XauB cells grown in pathogenicity non-inducing medium (NB) for 25 h or 40 h, respectively, from periplasm-enriched fraction resolved on 2-DE ($p < 0.05$).

Spot	Bacterium (unique spot)	NCBI accession number	Homologue protein[a] (Exclusive unique peptides count)[b]	Theoretical[c] MW (kDa) / pI	Experimental[d] MW (kDa) / pI	Mascot Score	Matched Peptide	Sequence Coverage %	Category of the XAC (homologue) protein[e]	Cellular Location[f]
1	XAC	XAC1362	GTN reductase (4)	39.4 / 5.4	40.8 / 6.2	630	30	64	I	C
2	XAC	XAC1129	3-oxoacyl-[ACP] synthase II	43.0 / 5.6	45.2 / 6.4	370	22	43	II	MC
3	XAC	XACb0007	Lytic murein transglycosylase	46.2 / 5.9	43.6 / 6.4	2290	49	65	IV	M+
		XAC3602	Cystathionine γ-lyase-like protein	42.7 / 5.7		51	10	22	II	C
		XAC1129	3-oxoacyl-[ACP] synthase II	43.0 / 5.6		24	3	11	II	MC
4	XAC	XAC3851	Conserved hypothetical protein(14)	50.0 / 5.3	49.9 / 5.8	954	39	56	VIII	-
		XAC3556	Aminopeptidase A/I	51.5 / 5.2		58	3	7	III	C
		XACb0007	Lytic murein transglycosylase	46.2 / 5.9		51	2	4	IV	M+
		XAC1829	Histidinol dehydrogenase (2)	44.8 / 5.3		48	3	5	II	C
		XAC2378	Conserved hypothetical protein	48.8 / 5.2		46	1	3	VIII	C
		XAC1808	Aldehyde dehydrogenase	54.4 / 5.4		37	1	3	I	C
		XAC0536	Virulence regulating protein	37.9 / 8.7		32	1	2	VII	C
5	XAC	XAC3587	Electron transfer flavoprotein α-subunit (9)	31.8 / 4.9	29.8 / 5.2	76	24	54	I	M
		XAC3924	Spermidine synthase	31.9 / 4.9		40	1	9	II	C
		XAC2926	Pyrroline-5-carboxylate reductase	28.9 / 5.1		40	1	4	II	C
6	XAC	XAC2012	3-ketoacyl-CoA thiolase (9)	42.1 / 6.0	33.2 / 6.4	457	20	47	I	C
		XAC2352	Ornithine carbamoyltransferase (4)	38.5 / 5.9		30	2	9	IV	MC+
		XACb0007	Lytic murein transglycosylase	46.2 / 5.9		30	2	9	IV	M+
7	XAC	XAC1006	Malate dehydrogenase (19)	34.9 / 5.4	37.7 / 5.8	1419	46	86	I	M
8	XAC	XAC1788	Glucose-6-phosphate isomerase (14)	54.4 / 5.5	58.2 / 6.3	1487	37	40	I	C
9	XAC	XAC0002	DNA polymerase III β subunit (21)	40.8 / 5.4	46.1 / 6.0	1703	58	70	III	C
		XAC0957	elongation factor Tu	43.3 / 5.5		154	6	21	III	C
		XAC1158	Adenylosuccinate synthetase	46.5 / 5.4		88	2	3	II	C

		XAC1788	Glucose-6-phosphate isomerase (14)	54.4 / 5.5		63	2	7	I	C
10	XAC	XAC1587	Thiosulfate sulfurtransferase (6)	30.7 / 6.1	31.8 / 7.1	188	13	57	I	C
11	XAC	XAC4009	Arginase (2)	33.4	33.4 / 5.1	91	4	13	I	C
12	XAC	XAC2516	L-lysine 6-aminotransferase (6)	53.8 / 6.0	60.0 / 7.0	386	18	32	I	C
13	XAC	XAC0749	GTP cyclohydrolase II/3,4-dihydroxy-2-butanone 4-phosphate synthase (11)	41.3 / 5.5	44.3 / 6.2	657	26	44	II	C
		XACb0007	Lytic murein transglycosylase	46.2 / 5.9		89	5	5	IV	M+
		XAC3602	Cystathionine gamma-lyase-like protein	42.7 / 5.7		39	1	3	II	C
		XAC1858	Valine-pyruvate aminotransferase	46.1 / 6.2		22	1	3	II	P
14	XAC	XAC3341	Cysteine synthase (4)	34.1 / 5.5	37.4 / 5.5	208	19	41	II	MC
		XAC1434	Conserved hypothetical protein	38.7 / 5.9		155	15	42	VIII	M+
15	XAC	XAC1046	Isocitrate dehydrogenase	35.7 / 5.4	40.1 / 5.9	583	19	34	I	C
		XAC1830	Histidinol-phosphate aminotransferase	39.0 / 5.4		35	3	7	III	C
		XAC0784	Cell division protein	41.8 / 4.8		30	2	1	V	C
		XAC0511	Phosphoribosylamine-glycine ligase	45.0 / 5.4		26	2	1	II	C
16	XAC	XAC3456	3-Isopropylmalate dehydrogenase (14)	38.3 / 5.3	41.1 / 5.7	957	24	61	II	C
		XAC1126	Malonyl CoA-ACP transacylase (8)	32.9 / 5.6		716	16	58	II	C
		XAC1550	FKBP-type peptidyl-prolyl cis-trans isomerase (3)	31.7 / 6.4		129	6	29	III	ME
		XAC1046	Isocitrate dehydrogenase (6)	35.7 / 5.4		80	4	15	I	C
17	XAC	XAC1362	GTN reductase (4)	39.4 / 5.4	41.9 / 5.9	1428	45	71	I	C
		XAC1006	Malate dehydrogenase (19)	34.9 / 5.4		119	6	19	I	M
		XAC4040	Δ-aminolevulinic acid dehydratase (2)	36.6 / 5.4		88	4	7	II	C
18	XAC	XAC3443	Response regulator (7)	40.7 / 4.9	42.2 / 5.4	1104	27	44	I	M
		XAC3347	Phosphoglycerate kinase (2)	40.9 / 4.9		151	7	17	I	C
		XAC1362	GTN reductase (4)	30.4 / 5.4		53	3	20	I	C
		XAC1719	Enolase	46.0 / 5.0		37	1	4	I	C
19	XauB	XAUB_15890	Dihydrolipoamide acetyltransferase (10)	42.4 / 5.9	52.7 / 6.4	348	22	34	I (XAC1534)	C
20	XauB	XAUB_39030	3.4-dihydroxy-2-butanone 4-phosphate synthase	38.3 / 6.1	41.7 / 5.7	302	22	20	II (XAC0749)	C

21	XauB	XAUB_20360	Pyruvate kinase (19)	54.8 / 5.6	57.3 / 5.5	429	18	21	I (XAC3345)	C
22	XauB	XAUB_14690	Type III secretion system hopAJ-like protein	46.4 / 6.1	42.5 / 5.8	161	6	7	IV (XAC3225)	M+
		XAUB_07370	Carbamoyl-phosphate synthase small subunit	47.8 / 6.3		128	9	7	II (XAC1861)	C
23	XauB	XAUB_30130	Oxidoreductase (16)	40.1 / 6.0	39.6 / 5.4	503	29	36	I (XAC1362)	-
		XAUB_29780	Oxidoreductase. FAD/FMN binding	46.8 / 6.2		42	4	2	I (XAC1362)	-
24	XauB	XAUB_26290	Secreted protein (20)	40.9 / 6.2	34.7 / 5.9	392	16	34	VIII (XAC1434)	M+
25	XauB	XAUB_20300	Glyceraldehyde-3-phosphate dehydrogenase (4)	36.2 / 6.0	37.9 / 6.3	201	17	33	I (XAC3352)	-
26	XauB	XAUB_06050	TldD protein (10)	48.7 / 6.0	45.7 / 6.3	414	26	40	III (XAC0120)	-
		XAUB_32940	Homogentisate 1.2-dioxygenase	48.3 / 5.8		92	10	21	I (XAC0454)	-
27	XauB	XAUB_22500	Dihydrodipicolinate synthetase (14)	32.0 / 5.7	27.2 / 6.2	787	29	45	II (XAC2547)	C
28	XauB	XAUB_40390	Translation elongation factor (12)	76.0 / 5.1	76.7 / 5.3	551	29	33	III (XAC0969)	C
		XAUB_14310	Phosphoenolpyruvate synthase (7)	86.3 / 5.2		344	19	17	II (XAC2041)	-
		XAUB_17460	Oligopeptidase B (3)	78.9 / 5.2		129	5	6	III (XAC0631)	P
29	XauB	XAUB_14750	Chaperonin GroEL (4)	56.9 / 5.0	54.7 / 5.1	1094	43	44	III (XAC0542)	-
		XAUB_23450	Succinyl-diaminopimelate desuccinylase	54.0 / 5.0		164	8	7	II	C
30	XauB	XAUB_05710	Superoxide dismutase	22.7 / 5.5	24.5 / 6.0	364	18	34	VII (XAC2386)	P
31	XauB	XAUB_23350	Secreted protein (4)	40.9 / 6.2	34.5 / 6.2	3262	59	78	VIII (XAC1434)	-
		XAUB_11090	PhoH-like ATP-binding protein	36.0 / 5.8		71	1	4	VIII (XAC2462)	C
		XAUB_25490	Two-component system sensor protein	126.8 / 9.2		33	2	0	IX (XAC4193)	ME
		XAUB_40720	Malate dehydrogenase	35.0 / 5.4		20	2	5	I (XAC1006)	M
32	XauB	XAUB_31810	Citrate Si –synthase	50.5 / 5.8	40.6 / 5.5	574	32	52	I (XAC3388)	C
		XAUB_28270	Leucine aminopeptidase	49.1 / 6.0		178	10	16	III (XAC3987)	C
		XAUB_20360	Pyruvate kinase (20)	54.8 / 5.6		100	9	11	I (XAC3345)	C
		XAUB_40270	Elongation factor Tu (8)	43.3 / 5.5		28	4	15	III (XAC0957)	C
33	XauB	XAUB_20360	Pyruvate kinase (20)	54.8 / 5.6	56.9 / 5.7	1828	52	57	I (XAC3345)	C
		XAUB_39520	S-adenosyl-L-homocysteinase hydrolase	53.2 / 5.5		332	12	18	I (XAC0804)	C
		XAUB_31330	5-phosphoribosylglycinamide formyltransferase 2	43.0 / 5.6		37	1	2	II (XAC1237)	MC

34	XauB	XAUB_41490	3-oxoacyl-acyl carrier protein synthase	43.1 / 5.5	42.5 / 5.9	642	34	55	II (XAC1129)	MC
		XAUB_31330	5- phosphoribosylglycinamide formyltransferase 2	43.0 / 5.6		254	11	20	II (XAC1237)	MC
		XAUB_14690	Type III secretion system hopAJ-like protein	46.4 / 6.1		64	2	2	IV (XAC3225)	M+
		XAUB_31810	Citrate Si-synthase	50.5 / 5.8		30	2	8	I (XAC3388)	C
35	XauB	XAUB_26520	Fumarate hydratase (10)	54.9 / 5.6	52.1 / 5.8	376	20	32	I (XAC1460)	C
		XAUB_23170	Trehalase	62.3 / 5.9		47	2	5	V (XAC0604)	P
		XAUB_39240	UDP-N-acetylmuramoylalanyl-D-glutamyl-2.6-diaminopimelate-D-alanyl-D-alanyl ligase	51.6 / 5.5		43	3	7	IV (XAC0776)	C
36	XauB	XAUB_38360	6-phosphogluconate dehydrogenase (3)	32.9 / 5.6	30.6 / 5.9	373	13	27	I (XAC0680)	C
		XAUB_27380	2-dehydro-3-deoxyphosphooctonate aldolase (2)	30.0 / 5.6		97	6	11	IV (XAC1717)	C
37	XauB	XAUB_25830	β-glucosidase (3)	77.7 / 5.2	75.3 / 5.3	154	16	27	I (XAC3869)	P
		XAUB_15760	molecular chaperone DnaK	68.8 / 5.0		45	1	1	III (XAC1522)	C
		XAUB_26090	Isocitrate dehydrogenase	80.4 / 5.6		40	7	11	I (XAC3835)	C
38	XauB	XAUB_39280	UDP-N-acetylmuramate-L-alanine ligase	50.7 / 5.6	50.1 / 6.0	694	26	39	IV (XAC0780)	C
		XAUB_07240	Oxidoreductase (16)	51.1 / 5.6		15	2	5	I (XAC1845)	C
39	XauB	XAUB_28090	Tryptophanyl-tRNA synthetase (8)	46.9 / 5.6	44.9 / 6.0	432	17	26	III (XAC4006)	C
		XAUB_40270	Elongation factor Tu (8)	43.3 / 5.5		135	6	19	III (XAC0957)	C
		XAUB_40260	GTP-binding protein	39.1 / 5.0		18	4	10	V (XAC0953)	C
40	XauB	XAUB_15890	Dihydrolipoamide acetyltransferase (10)	42.4 / 5.9	45.8 / 6.1	478	28	50	I (XAC1534)	C
41	XauB	XAUB_09030	Xylose isomerase (10)	49.0 / 5.4	48.4 / 5.9	443	19	43	I (XAC4225)	C
		XAUB_40270	Elongation factor Tu (8)	43.4 / 5.5		442	15	32	III (XAC0957)	C
		XAUB_05050	Adenylosuccinate synthetase (4)	46.5 / 5.4		73	3	9	II (XAC1158)	C
		XAUB_40910	Dihydrofolate synthase (2)	45.2 / 5.3		49	4	10	II (XAC1029)	C
42	XauB	XAUB_15950	Conserved hypothetical protein (10)	44.0 / 5.2	46.5 / 5.5	549	28	50	VIII (XAC0141)	C
		XAUB_18590	Peptidyl-prolyl cis-trans isomerase (14)	50.1 / 5.4		428	25	36	III (XAC0865)	P
		XAUB_14210	UDP-N-acetylglucosamine 1-carboxyvinyltransferase	44.6 / 5.3		32	3	6	IV (XAC2965)	C
43	XauB	XAUB_13340	Leucyl aminopeptidase (21)	51.4 / 5.1	47.8 / 5.2	1817	61	76	III (XAC3556)	C

44	XauB	XAUB_15880	Dihydrolipoamide dehydrogenase (14)	50.7 / 5.8	48.3 / 6.3	940	33	46	I (XAC1533)	C
		XAUB_13340	Leucyl aminopeptidase (21)	51.4 / 5.1		137	5	12	III (XAC3556)	C
45	XauB	XAUB_32290	Adenylate kinase (5)	19.9 / 5.3	19.7 / 5.5	546	17	52	II (XAC3437)	C
		XAUB_13720	Outer-membrane lipoprotein carrier protein precursor (2)	24.7 / 9.3		109	5	17	III (XAC2008)	P+
46	XauB	XAUB_30350	Hypoxanthine-guanine phosphoribosyltransferase (6)	20.3 / 5.1	20.4 / 5.4	639	22	61	II (XAC1335)	C
47	XauB	XAUB_22330	Alkyl hydroperoxide reductase subunit C (9)	20.6 / 6.2	21.5 / 6.9	351	23	69	VII (XAC0907)	C
		XAUB_06900	Ribosome releasing factor (2)	20.3 / 6.8		58	3	14	III (XAC1418)	C
48	XauB	XAUB_30130	Oxidoreductase (16)	46.8 / 6.2	41.0 / 5.4	1335	41	39	I (XAC1362)	C
		XAUB_20360	Pyruvate kinase (19)	54.8 / 5.6		437	10	21	I (XAC3345)	C
49	XauB	XAUB_09270	Peptidase (9)	55.3 / 5.2	55.4 / 5.4	791	25	25	-	-
		XAUB_16080	Aldehyde dehydrogenase	55.8 / 5.3		48	5	10	VII (XAC0129)	C
		XAUB_22340	Alkyl hydroperoxide reductase subunit F	57.2 / 5.3		39	3	6	VII (XAC0906)	MC
50	XauB	XAUB_22500	Dihydrodipicolinate synthetase (14)	32.0 / 5.7	28.4 / 5.9	1346	33	56	II (XAC2547)	C
51	XauB	XAUB_28680	Pteridine-dependent deoxygenase-like protein	36.7 / 5.5	34.7 / 5.9	133	6	24	VII (XAC4103)	C
		XAUB_26290	Secreted protein (20)	40.9 / 6.2		29	8	25	VIII (XAC1434)	M

XAC and XauB were grown in NB medium, and periplasm-enriched fractions, extracted from both bacteria, were resolved on 2-DE ($p < 0.05$), and proteins from differential spots were identified by ESI-Q-TOF. The cultivation times were for XAC 25 h and for XauB 40 h. [a] Proteins identified using Mascot with XAC or XauB databases (NCBI); not all XauB proteins identified were included, only those ones that presented the highest Mascot scores and/or molecular weight (MW) and isoelectric point (p*I*) more compatible with experimental values; [b] Exclusive peptides count determined for some spots using Scaffold™ software (Proteome Software Inc., Portland, OR) for 100% protein identification probability; [c] Theoretical MW and p*I* of the matched protein obtained from the NCBI database; [d] Experimental molecular weight (MW) and isoelectric point (p*I*) calculated by Image Master Platinum software (GE Healthcare) based on the position of the spot on 2-DE; [e] Proteins clustering according to "*Xanthomonas axonopodis* pv. *citri* Main Chromosome and Plasmid Gene List" at NCBI [6]: I) Intermediary metabolism, II) Biosynthesis of small molecules, III) Macromolecule metabolism, IV) Cell structure, V) Cellular processes, VI) Mobile genetic elements, VII) Pathogenicity, virulence and adaptation, VIII) Hypothetical, IX) ORFs with undefined category; [f] Predicted cellular location of proteins by pSortP 3.0 and SecretomeP 2.0. P, M, and C correspond respectively to periplasm, membrane, and cytoplasm location. Signal (+) indicates the presence of signal peptide according to SignalPeptide 2.0.
