# Supplementary material for: Periplasm-enriched fractions from Xanthomonas citri subsp. citri type A and X. fuscans subsp. aurantifolii type B present distinct proteomic profiles under in vitro pathogenicity induction
Source: PLoS One. 2020 Dec 18;15(12):e0243867. doi: 10.1371/journal.pone.0243867 (PMC7748154; doi:10.1371/journal.pone.0243867)
Supplement: S4 Table — (PDF) [file pone.0243867.s007.pdf]

Table S4. Number of non-redundant proteins identified by MS-MS analysis in differential 2DE spots of the periplasm-enriched fractions of XAC and/or XauB after *in vitro* growth in XAM-M (pathogenicity-inducing) and NB (pathogenicity non- inducing) culture media.

**Culture medium**	**Number of non-redundant proteins identified in differential 2D spots between XAC and XauB for each culture medium**		
	XAC spots	XauB spots	XAC and XauB spots
XAM-M	12	48	0
NB	38	57	8
